# Cloning of Glycerophosphocholine Acyltransferase (GPCAT) from Fungi and Plants: A NOVEL ENZYME IN PHOSPHATIDYLCHOLINE SYNTHESIS*[Fn FN2]

**DOI:** 10.1074/jbc.M116.743062

**Published:** 2016-10-07

**Authors:** Bartosz Głąb, Mirela Beganovic, Sanket Anaokar, Meng-Shu Hao, Allan G. Rasmusson, Jana Patton-Vogt, Antoni Banaś, Sten Stymne, Ida Lager

**Affiliations:** From the ‡ Intercollegiate Faculty of Biotechnology, University of Gdańsk and Medical University of Gdańsk, 80-822 Gdańsk, Poland,; the § Department of Plant Breeding, Swedish University of Agricultural Sciences, 230 53 Alnarp, Sweden,; the ¶ Department of Biological Sciences, Duquesne University, Pittsburgh, Pennsylvania 15282, and; the ‖ Department of Biology, Lund University, Biology Building A, Sölvegatan 35, 223 62 Lund, Sweden

**Keywords:** acyltransferase, glycerophospholipid, lipid metabolism, phosphatidylcholine, plant biochemistry

## Abstract

Glycero-3-phosphocholine (GPC), the product of the complete deacylation of phosphatidylcholine (PC), was long thought to not be a substrate for reacylation. However, it was recently shown that cell-free extracts from yeast and plants could acylate GPC with acyl groups from acyl-CoA. By screening enzyme activities of extracts derived from a yeast knock-out collection, we were able to identify and clone the yeast gene (*GPC1*) encoding the enzyme, named glycerophosphocholine acyltransferase (GPCAT). By homology search, we also identified and cloned GPCAT genes from three plant species. All enzymes utilize acyl-CoA to acylate GPC, forming lyso-PC, and they show broad acyl specificities in both yeast and plants. In addition to acyl-CoA, GPCAT efficiently utilizes LPC and lysophosphatidylethanolamine as acyl donors in the acylation of GPC. GPCAT homologues were found in the major eukaryotic organism groups but not in prokaryotes or chordates. The enzyme forms its own protein family and does not contain any of the acyl binding or lipase motifs that are present in other studied acyltransferases and transacylases. *In vivo* labeling studies confirm a role for Gpc1p in PC biosynthesis in yeast. It is postulated that GPCATs contribute to the maintenance of PC homeostasis and also have specific functions in acyl editing of PC (*e.g.* in transferring acyl groups modified at the *sn-*2 position of PC to the *sn-*1 position of this molecule in plant cells).

## Introduction

**FIGURE 1.**
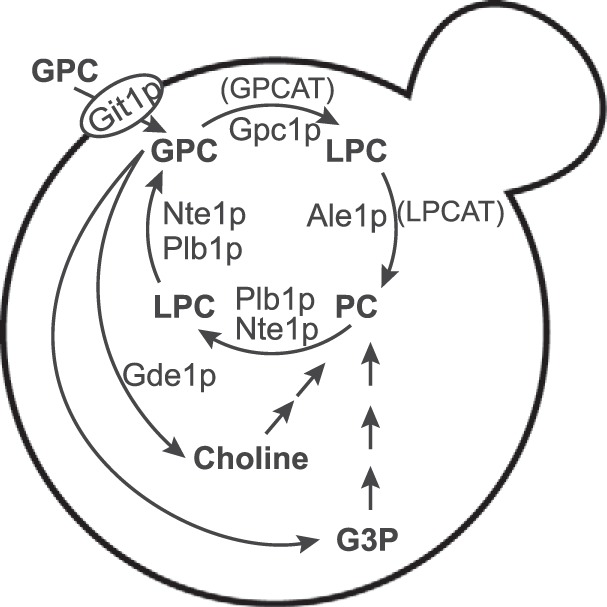
**Schematic outline of GPC metabolism in yeast.** Yeast gene products are indicated. Generic terms for some enzymatic steps that apply to yeast and plants are shown in *parenthesis*. For the sake of simplicity, the PE methylation and Kennedy pathways for PC biosynthesis are not shown.

Phosphatidylcholine (PC)^2^ is the most abundant membrane lipid in all non-photosynthetic eukaryotes and in extraplastidic membranes in photosynthetic eukaryotes. It also has a central role in membrane homeostasis through the remodeling of its acyl groups in response to changing environmental and metabolic conditions (1). In plants, PC acyl chain modifications produce multiple polyunsaturated and unusual fatty acids that are delivered from PC, through various pathways, to other lipids, including the storage lipid triacylglycerol (2). In yeast and other eukaryotic cells, the established pathways for PC biosynthesis are the CDP-choline pathway and the phosphatidylethanolamine (PE) methylation pathways. Once formed, the common pathway for PC turnover is the hydrolysis of acyl groups by phospholipases of the A or B type. Hydrolysis by an A type enzyme results in free fatty acid and lysophosphatidylcholine (LPC). The remaining acyl group of LPC can be hydrolyzed by phospholipases of the A or B type to yield free fatty acids and glycero-3-phosphocholine (GPC). GPC is known to be catabolized to free choline and glycerol 3-phosphate (G3P) (3–5), and the resulting choline is used in the *de novo* synthesis of PC via the CDP-choline pathway (6). Importantly, direct acylation of GPC by an acyl-CoA-dependent activity was recently demonstrated in *Saccharomyces cerevisiae* cell-free extracts and microsomal preparations (7). The putative enzyme carrying out the acylation of GPC was named GPC acyltransferase (GPCAT) (see Fig. 1). Recently, GPCAT activities were also demonstrated in microsomal preparations from developing seeds from different plant species as well as in *Arabidopsis* roots and shoots (8). To a varying extent, the different microsomal preparations incorporated radioactive [^14^C]GPC into both LPC and PC even in the absence of acyl-CoA (8).

In this work, we screened a yeast deletion library to identify the yeast gene encoding GPCAT, which we have termed *GPC1*.^3^ By homology search, we have also identified GPCAT encoding genes from plant species. In addition, we report that not only can GPCAT enzymes utilize acyl-CoA to acylate GPC, but they can also catalyze the transacylation of acyl groups from LPC to GPC. The latter reaction explains the earlier reported incorporation of GPC into lipids in the absence of added acyl-CoA in assays with microsomal preparation from developing oil seeds (8). The GPCAT does not show any significant homology to other known acyl transferases and constitutes an enzyme family on its own with homologues represented in major eukaryotic organism groups but absent in prokaryotes.

## Results

### 

#### 

##### Identification of the Yeast GPCAT-encoding Gene

**FIGURE 2.**
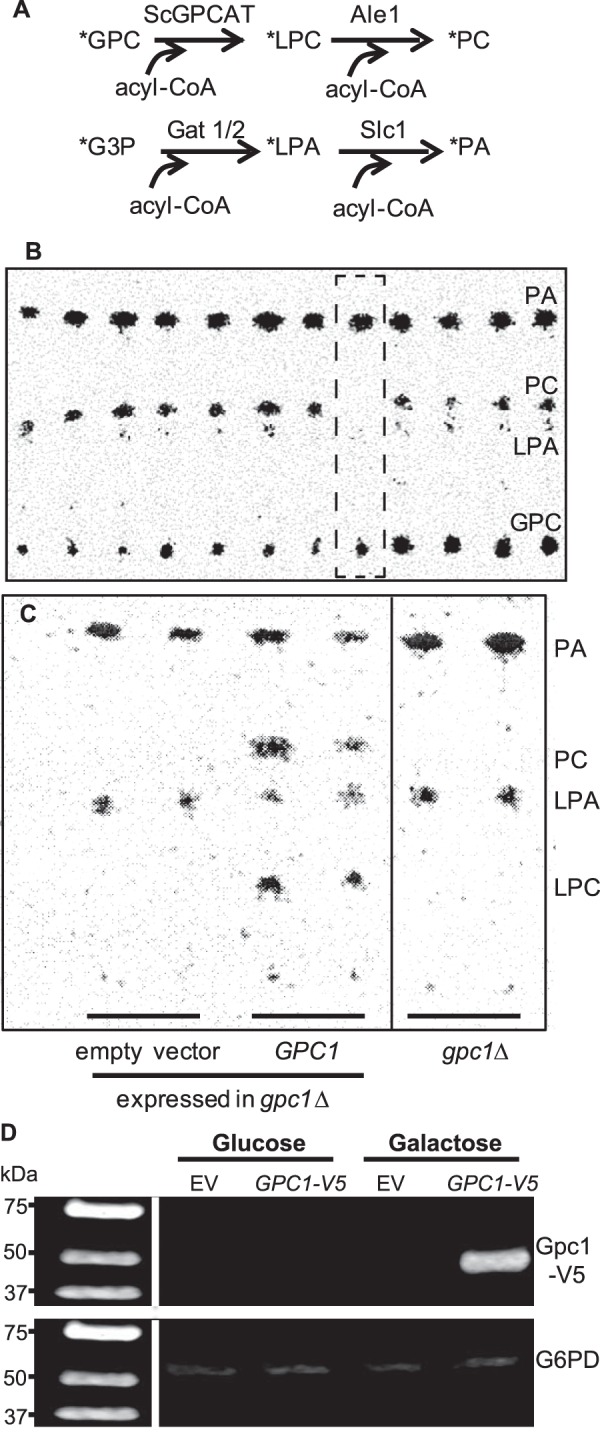
**Identification of ScGPCAT (*GPC1*).** Yeast extracts of strains from the yeast deletion collection were incubated with [^14^C]GPC and 18:1-CoA. [^14^C]G3P was added to the incubation as an internal control of enzymatic activities. *A*, enzyme reactions in yeast from the substrates [^14^C]GPC and [^14^C]G3P. The yeast-acylating enzymes are indicated. *B*, autoradiogram of the screening on thin layer chromatography plates showing the deletion strain lacking GPCAT activity (within the *hatched square*). *C*, complementation of *YGR149W* (*GPC1*) in the *gpc1*Δ deletion strain. Empty vector as well as the deletion strain (*gpc1*Δ) are also included. Irrelevant lanes from the plate have been removed from the picture (marked by a *vertical line*). *D*, Western blot of Gpc1p. Wild type strain bearing EV or a plasmid (*GPC1-V5*) harboring *GPC1* under the control of the galactose-inducible *GAL1* promoter and containing a C-terminal V5 epitope. Cells were grown on either glucose or galactose. Equivalent amounts of protein (75 μg) were loaded onto each lane. Anti-Gpc1p-V5 primary antibody and goat anti-mouse secondary antibody were employed. The blot was visualized using an Odyssey FC imaging system. G6PD was used as the loading control.

A subset of the yeast knock-out collection was used to screen for the GPCAT-encoding gene. Only strains carrying deletions in genes with no known or putative function and with a size of at least 500 bp were included. This group contained ∼600 strains. Yeast extracts from the deletion strains were incubated with [^14^C]choline-labeled GPC, acyl-CoA and, as a control, [^14^C]G3P; the lipids were separated by thin layer chromatography and visualized on an Instant Imager electronic autoradiograph. In the reaction, the putative GPCAT utilized acyl-CoA to convert GPC to LPC, which was further acylated to PC by the endogenous Ale1p. G3P was added to the assay as an internal control to validate the quality of the yeast extract and to provide a ratio of G3P to GPC acylation activity for detecting decreased GPCAT activity. G3P is acylated to lysophosphatidic acid (LPA) by the enzymes Gat1p and Gat2p, and the formed LPA is converted to phosphatidic acid (PA) by Slc1p (Fig. 2*A*). We identified a deletion strain lacking GPCAT activity, but exhibiting G3P acylation after ∼200 screened strains, and the remaining strains were not screened (Fig. 2*B*). The identified strain bears a deletion in ORF *YGR149W*, here named *GPC1*, which is annotated as a putative protein of unknown function in the *Saccharomyces* Genome Database. The gene was amplified, cloned into a pYES-based yeast vector, and expressed in the identified deletion strain. As can be seen in Fig. 2*C*, *GPC1* complemented the deletion strain. *GPC1* contains only one defined domain of no known function (DUF2838) and has no known acyl binding domains. The *S. cerevisiae* GPCAT (ScGPCAT) or Gpc1p is a protein of 52 kDa that is predicted to be an integral membrane protein with eight transmembrane helices by the TMHMM server (9). Western blotting analysis (Fig. 2*D*) of a V5 epitope-tagged version of Gpc1p under the control of the *GAL1* promoter (*GAL1-GPC1-V5*) reveals a protein of the expected molecular weight in cells grown in the presence of galactose.

##### Evolutionary Analysis

**FIGURE 3.**
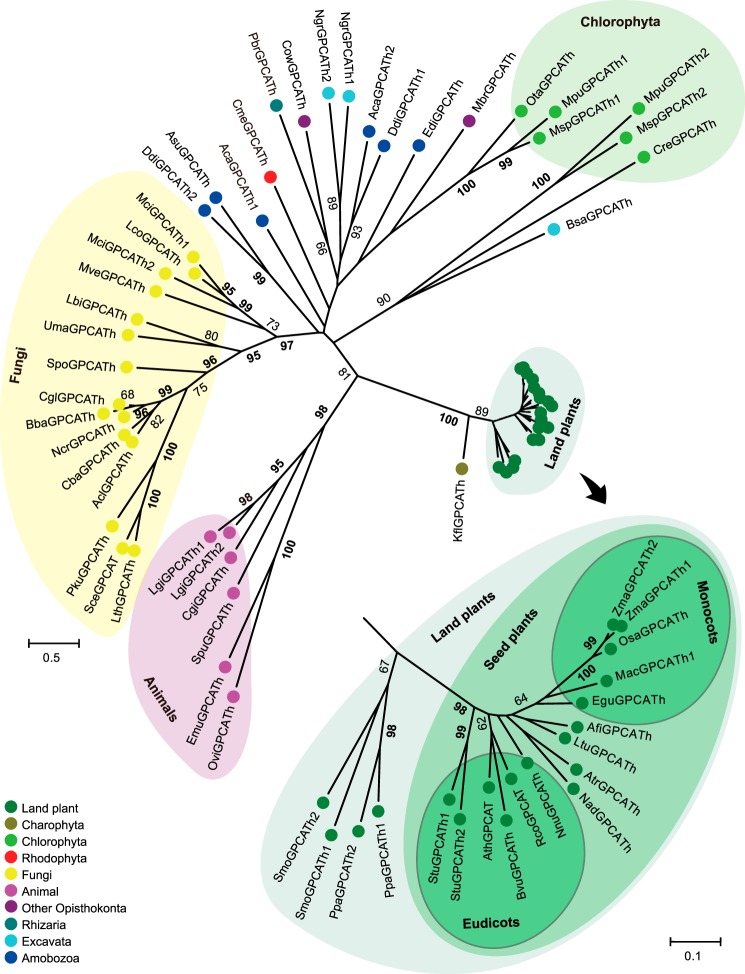
**Evolution of the GPCAT family in eukaryotes.** GPCAT homologues from selected species across the eukaryotic clade were aligned and a radial unrooted tree built by maximum likelihood analysis using an LG+G model. The tree is drawn to scale, and branch lengths are measured in substitutions per site. Bootstrap values above 60 are shown (in percentages). Values of at least 95 are in *boldface type. Inset*, an expanded subtree for the land plant clade is shown. *Aca*, *Acanthamoeba castellanii* str. *Neff*; *Acl*, *Aspergillus clavatus* NRRL 1; *Afi*, *Aristolochia fimbriata*; *Asu, Acytostelium subglobosum LB1*; *Ath*, *Arabidopsis thaliana*; *Atr*, *Amborella trichopoda*; *Bba*, *Beauveria bassiana* D1-5; *Bsa*, *Bodo saltans*; *Bvu*, *Beta vulgaris* subsp. *Vulgaris*; *Cba*, *Cladophialophora bantiana* CBS 173.52; *Cgi*, *Crassostrea gigas*; *Cgl*, *Chaetomium globosum* CBS 148.51; *Cme*, *Cyanidioschyzon merolae* strain 10D; *Cow*, *Capsaspora owczarzaki* ATCC 30864; *Cre*, *Chlamydomonas reinhardtii*; *Ddi*, *Dictyostelium discoideum* AX4; *Edi*, *Entamoeba dispar* SAW760; *Egu*, *Elaeis guineensis*; *Emu*, *Echinococcus multilocularis*; *Kfl*, *Klebsormidium flaccidum*; *Lbi*, *Laccaria bicolor* S238N-H82; *Lco*, *Lichtheimia corymbifera* JMRC:FSU:9682; *Lgi*, *Lottia gigantea*; *Lth*, *Lachancea thermotolerans* CBS 6340; *Ltu*, *Liriodendron tulipifera*; *Mac*, *Musa acuminata* subsp. *Malaccensis*; *Mbr*, *Monosiga brevicollis* MX1; *Mci*, *Mucorcircinelloides f. circinelloides* 1006PhL; *Mpu*, *Micromonas pusilla* CCMP1545; *Msp*, *Micromonas* sp. RCC299; *Mve, Mortierella verticillata* NRRL 6337; *Nad*, *Nuphar advena*; *Ncr*, *Neurospora crassa* OR74A; *Ngr*, *Naegleria gruberi* strain NEG-M; *Nnu*, *Nelumbo nucifera*; *Osa*, *Oryza sativa* Indica group; *Ota*, *Ostreococcus tauri*; *Ovi*, *Opisthorchis viverrini*; *Pbr*, *Plasmodiophora brassicae*; *Pku*, *Pichia kudriavzevii*; *Ppa*, *Physcomitrella patens*; *Rco*, *Ricinus communis*; *Sce*, *Saccharomyces cerevisiae* S288c; *Smo*, *Selaginella moellendorffii*; *Spo*, *Schizosaccharomyces pombe 972h*; *Spu*, *Strongylocentrotus purpuratus*; *Stu*, *Solanum tuberosum*; *Uma*, *Ustilago maydis 521*; *Zma*, *Zea mays*. See supplemental Fig. S1 for protein alignment and sequence IDs.

Database searches revealed a wide distribution of GPCAT homologues in eukaryotes with generally singletons or dual paralogs being present in each species analyzed (Fig. 3). No significant protein hits were found in prokaryotes, but homologues were found in major eukaryotic organism groups like fungi, animals, plants, algae, and several protist clades. However, homologues were not found in, for example, alveolates or heterokonts nor in animal subclades like chordates and arthropods. In the maximum likelihood tree (Fig. 3), proteins from animals, fungi, and Streptophyta (plants and Charophyta) grouped separately, as supported by significant bootstrap values. For other eukaryotes, basal resolution was not observed. Chlorophyta (green algae) separated into two groups, one of which was associated (bootstrap value of 90%) with *Bodo saltans* (a euglenozoa belonging to Excavata). Significant groups were also observed for some Amobozoa proteins.

GPCAT was found present in all analyzed major groups of plants. However, the plant clade was characterized by displaying a very small sequence divergence as compared with other organism groups. The plant group revealed a significant group containing seed plants, separate from the moss *Physcomitrella patens* and the lycopod *Selaginella moellendorffii*. The monocot proteins were weakly associated (bootstrap value of 64%), but the major groups of other plants were not resolved.

##### ScGPCAT (Gpc1p) Catalyzes the Acyl-CoA-dependent Acylation of GPC

**FIGURE 4.**
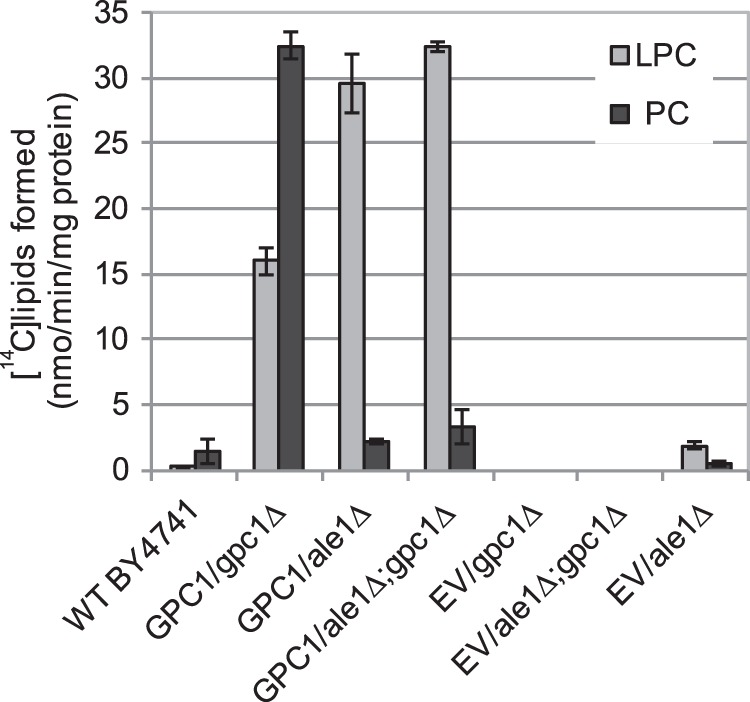
**Radioactive lipids formed by microsomes from the indicated strains.** Microsomes were prepared from the indicated yeast strains bearing EV or expressing *GPC1* under the *GAL1* promoter (*GPC1*). The WT yeast was grown in YPD, and yeast strains carrying a plasmid were grown in YNB medium containing galactose. Microsomes (2 μg of protein) were incubated with [^14^C]choline-labeled GPC (0.24 mm) and 18:1-CoA (0.2 mm) for 30 min at 30 °C. The results shown are from triplicate assays ± S.D. (*error bars*).

**FIGURE 5.**
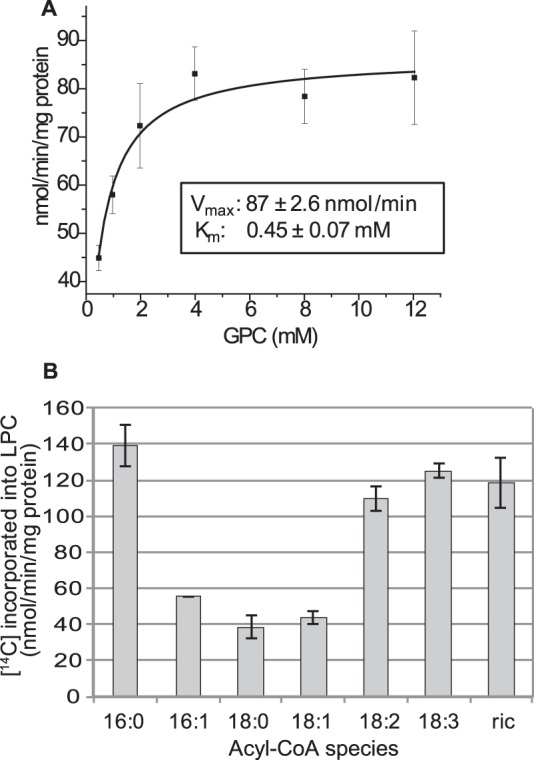
**Biochemical characterization of Gpc1p.** *A*, dependence of Gpc1p activity on GPC concentration in the presence of 18:1-CoA. *B*, the acyl-specificity of Gpc1p at optimal GPC concentration (4 mm). The activity was measured as incorporation of ^14^C activity from [^14^C]choline-labeled GPC into chloroform-soluble lipids in the presence of acyl-CoA (0.2 mm). Microsomal preparations (2 μg of protein) prepared from *ale1*Δ *gpc1*Δ expressing *GAL1-GPC1* were incubated for 4 min at 30 °C. *ric*, ricinoleoyl-CoA. Data are given for triplicate assays ± S.D. (*error bars*).

The ScGPCAT activities were assayed with [^14^C]GPC and non-radioactive 18:1-CoA in microsomal preparations from wild type (WT; BY4741), *gpc1*Δ, *ale1*Δ, and *ale1*Δ *gpc1*Δ yeast strains expressing *GPC1* either under the control of the *GAL1* promoter or transformed with empty vector (Fig. 4). In WT, the main radioactivity in lipids was found in PC (85%), and the remaining radioactivity was found in LPC. Membranes from *ale1*Δ transformed with empty vector incorporated about the same amount of total radioactivity into lipid as WT, but only 21% of the radioactivity was found in PC with the remaining activity in LPC, indicating that Ale1p is primarily responsible for LPC acylation. Membranes from *gpc1*Δ bearing empty vector did not incorporate radioactivity into lipids, whereas membranes from *gpc1*Δ cells expressing *GPC1* incorporated about 30 times more radioactivity into lipids than WT. When GPCAT activity was measured in membranes from *ale1*Δ *gpc1*Δ harboring *GPC1*, the total radioactivity incorporated into lipid was reduced ∼25% as compared with the *gpc1*Δ background, and the activity now accumulated primarily in LPC (93%), demonstrating again that Ale1p is the main enzyme responsible for the formation of radioactive PC from LPC in these assays. The dependence of GPCAT activity on GPC concentration was determined in microsomal membranes prepared from *ale1*Δ *gpc1*Δ transformed with *GAL1-GPC1* using 18:1-CoA as the acyl donor (Fig. 5*A*). The *K_m_* for GPC was found to be 0.45 mm with a *V*_max_ of 87 nmol/min. The linearity of the reaction was tested with 18:1-CoA at optimal GPC concentration and was found to be essentially linear during 16 min of incubation under the incubation condition used (data not shown). The ScGPCAT accepted all of the acyl-CoA species tested with a preference for 16:0-CoA, polyunsaturated acyl-CoA, and the hydroxylated ricinoleoyl-CoA (Fig. 5*B*).

##### Plant Homologues of GPC1 Catalyze Acyl-CoA-dependent Acylation of GPC When Expressed in Yeast

**FIGURE 6.**
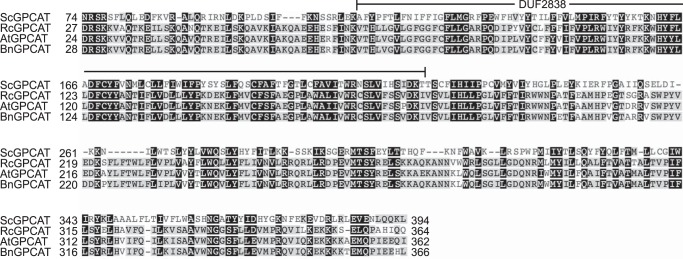
**Partial alignment of ScGPCAT (*S. cerevisiae*; NP_011665.1), RcGPCAT (*Ricinus communis*; XP_002514086.1), AtGPCAT (*A. thaliana*; NP_198396.1), and BnGPCAT (*B. napus*; XP_013682687.1).** Sequence numbering is according to the full-length protein sequences. The conserved DUF2838 domain is marked *above* the sequence (Val^68^–Ile^174^ in the *A. thaliana* sequence). Proteins were aligned using Clustal Omega, and Geneious was used for *shading background* according to conservation.

**FIGURE 7.**
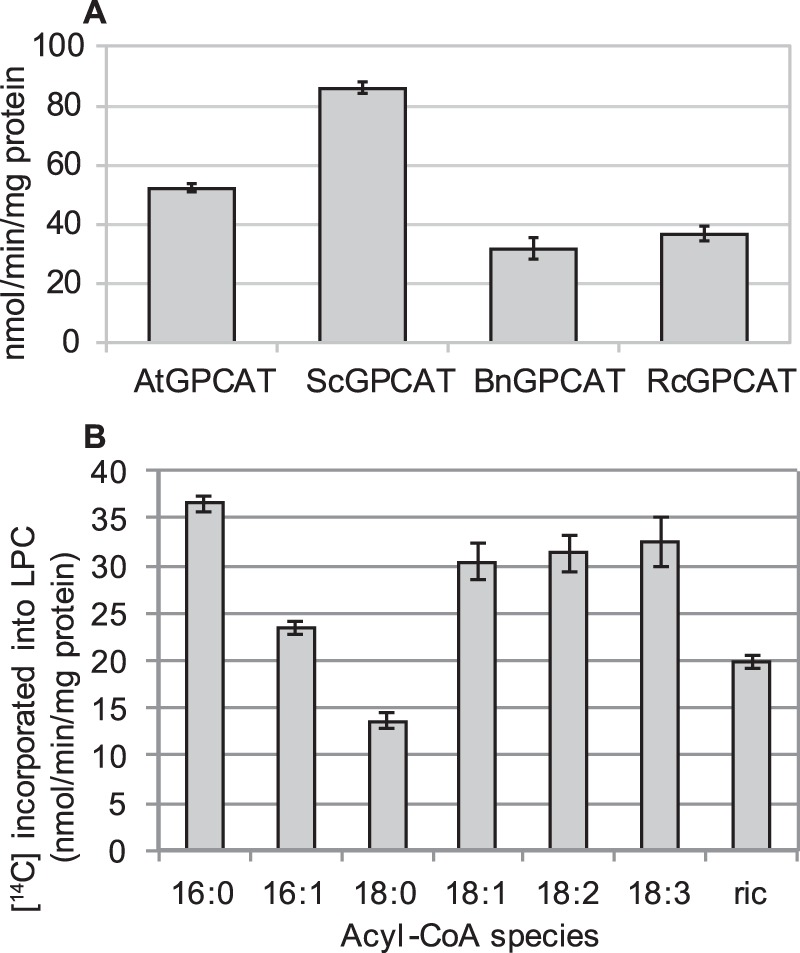
**Enzyme activities of plant GPCATs.** *A*, specific activity of *A. thaliana* GPCAT (*AtGPCAT*), *S. cerevisiae* GPCAT (*ScGPCAT*), *B. napus* GPCAT (*BnGPCAT*), and *R. communis* GPCAT (*RcGPCAT*). *B*, acyl-CoA specificity of the RcGPCAT. The activity was measured as incorporation of ^14^C activity from [^14^C]choline-labeled GPC (4 mm) into chloroform-soluble lipids in the presence of 18:1-CoA (0.2 mm) by microsomal preparations (2 μg (*A*) and 4 μg (*B*) of protein) prepared from *ale1*Δ gpc1Δ yeast cells expressing the different GPCATs. Incubations with microsomes prepared from *gpc1*Δ transformed with empty vector gave no incorporation of radioactivity into the chloroform phase. Incubation time was 8 min (*A*) and 4 min (*B*). Data are given for triplicate assays ± S.D. (*error bars*). Of the ^14^C activity incorporated in lipids, 98–99% was recovered as lysophosphatidylcholine, and 1–2% was recovered as phosphatidylcholine.

Sequence similarity was used to identify homologues in *Arabidopsis*, castor bean (*Ricinus communis*), and oilseed rape (*Brassica napus*). The *Arabidopsis* GPCAT shows 25% protein sequence identity to the yeast GPCAT in a 340-residue conserved part from Asp^24^ to Ile^362^ in the *A. thaliana* sequence (Fig. 6). The three different plant GPCATs were expressed in the yeast *gpc1*Δ*ale1*Δ strain under the *GAL1* promoter, and the results were compared with those obtained for ScGPCAT. The specific activity was highest with ScGPCAT (86 nmol/min/mg protein), whereas the plant GPCAT activities ranged from 32 to 52 nmol/min/mg protein (Fig. 7*A*). Microsomal preparations from castor bean seeds have high GPCAT activity (8), and the plant accumulates seed triacylglycerols with about 90% of the fatty acid being the hydroxy acid, ricinoleic acid. This fatty acid is synthesized by hydroxylation of oleoyl groups esterified to the *sn*-2 position of PC (10). It was therefore of interest to investigate the acyl-CoA specificity of the castor GPCAT. Like yeast GPCAT, the castor GPCAT accepted all of the acyl-CoAs tested, including ricinoleoyl-CoA (Fig. 7*B*), although with somewhat different acyl specificities as compared with the yeast enzyme.

##### Gpc1p Catalyzes Acylation of Glycerophosphoethanolamine (GPE) with Acyl-CoA

**FIGURE 8.**
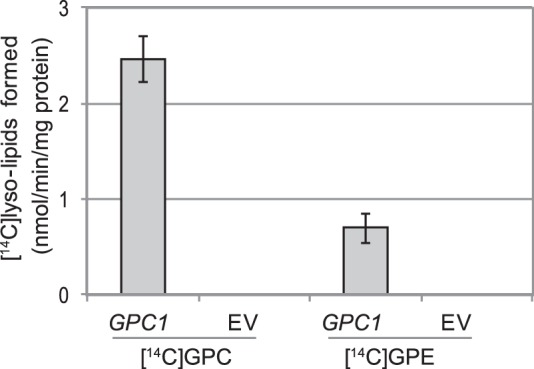
**Acylation of glycerophosphoethanolamine.** Shown is incorporation of ^14^C activity from [^14^C]choline-labeled GPC (0.5 mm) or [^14^C]ethanolamine-labeled GPE (0.5 mm) into chloroform-soluble lipids in the presence of 18:1-CoA (0.4 mm). Shown are microsomal preparations (40 μg of protein) of *ale1*Δ *gpc1*Δ yeast cells with overexpressed *GPC1* or EV. The radioactivity resided in LPC in assays with added [^14^C]GPC and in lysophosphatidylethanolamine in the case of added [^14^C]GPE. Incubation time was 20 min, and the temperature was 30 °C. Data are given for triplicate assays ± S.D. (*error bars*).

Stålberg *et al.* (7) reported that yeast also possessed acyl-CoA:GPE acyltransferase activity. To test whether this activity was catalyzed by ScGPCAT, we performed assays with [^14^C]GPE and 18:1 acyl-CoA in microsomal preparations of *ale1*Δ *gpc1*Δ strain transformed with *GPC1* or empty vector (Fig. 8). No incorporation of radioactivity into lipids was seen with empty vector, but upon expression of *GPC1*, radioactive lysophosphatidylethanolamine (LPE) accumulated, albeit at about 25% of the amount of LPC formed when [^14^C]GPC was provided. Thus, Gpc1p also catalyzes the acylation of GPE with acyl-CoA.

##### GPCATs from Yeast and Plants Catalyze Transacylation Reactions

**FIGURE 9.**
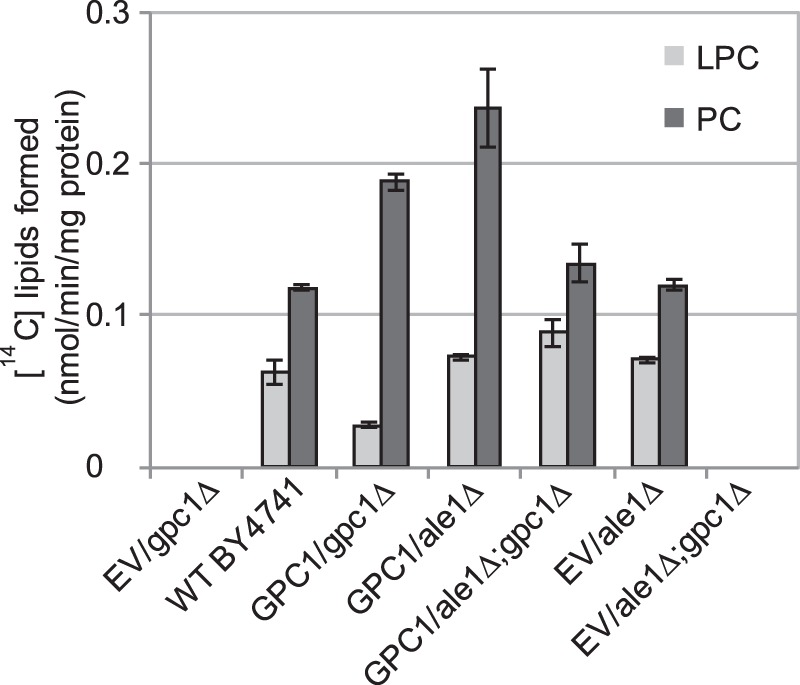
**Transacylation activity of GPCAT.** Radioactive lipids formed in incubations of microsomes (40 μg of protein) prepared from yeast lacking or expressing the indicated genes. Incubations contained [^14^C]glycerophosphocholine (0.24 mm) in the absence of added acyl-CoA and were performed for 90 min at 30 °C. The results shown are from triplicate assays ± S.D. (*error bars*). Strains contained EV or expressed *GPC1* from *GAL1* promoter. Yeast of WT that was used for the microsomal preparation was grown in YPD, and yeast strains containing plasmids were grown in YNB medium containing galactose.

**FIGURE 10.**
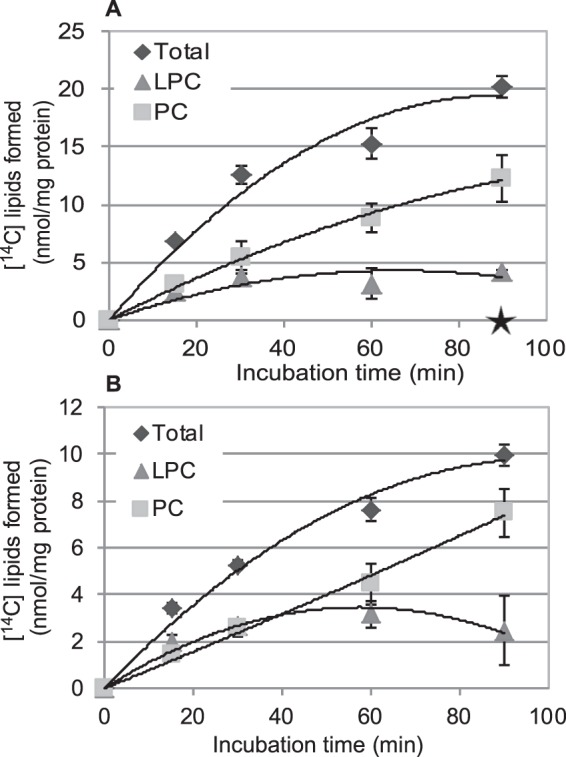
**Transacylation activity of GPCAT over time.** Shown is time course incorporation of ^14^C activity from [^14^C]choline-labeled GPC (4 mm) into LPC in incubations in the absence of added acyl-CoA by microsomal preparations (40 μg of protein) prepared from yeast *ale1*Δ *gpc1*Δ cells overexpressing ScGPCAT (*GPC1*) (*A*) or castor bean GPCAT (RcGPCAT) (*B*). The enzyme rates for ScGPCAT and RcGPCAT in the absence of acyl-CoA were 0.45 and 0.23 nmol/min/mg, respectively, during the first 15 min. Corresponding incubations with microsomes prepared from the *gpc1*Δ*ale1*Δ strain transformed with empty vector gave no incorporation of radioactivity into the chloroform phase, indicated with a *star* at 90 min of incubation in the *graph* (*A*). Data are given for triplicate assays ± S.D. (*error bars*).

The incorporation of [^14^C]choline-labeled GPC into lipid in the absence of exogenous acyl-CoA was assayed in yeast membranes (Fig. 9). Membranes from *gpc1*Δ did not incorporate any radioactivity into lipids, whereas the WT and the *gpc1*Δ transformed with *pGAL-GPC1* incorporated a similar amount of radioactivity into both LPC and PC. The lack of Ale1p had little or no effect on the incorporation of radioactivity into LPC or PC (Fig. 9). These experiments indicate that GPCAT is responsible for the production of radioactive LPC from [^14^C]GPC in the absence of exogenous acyl-CoA and that the second acylation step, the acylation of LPC to PC, is independent of Ale1p activity in the absence of exogenous acyl-CoA. Although GPCAT activity was essential for incorporation of radioactivity from [^14^C]GPC into lipids in the absence of acyl-CoA, its activity did not appear to be a rate-limiting factor in WT because the WT and *GPC1*-overexpressing strain incorporated similar amounts of radioactivity into lipids. A time course of incorporation of [^14^C]GPC into lipids in the absence of added acyl-CoA was performed with membranes from *ale1*Δ *gpc1*Δ cells transformed with ScGPCAT or RcGPCAT. The two enzymes gave similar incorporation patterns (Fig. 10).

**FIGURE 11.**
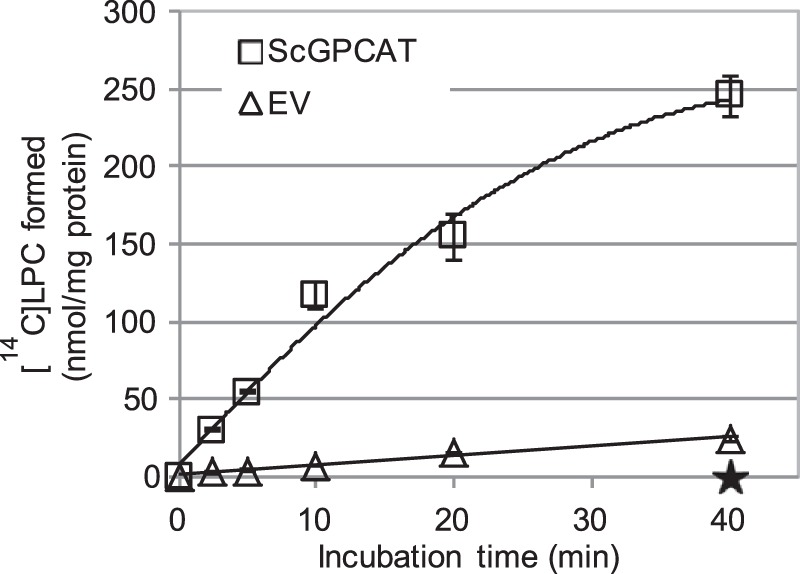
**Time course for transacylation activity of ScGPCAT in the presence of added LPC.** Shown is incorporation of ^14^C activity from [^14^C]choline-labeled GPC (1 mm) into lipids in the presence of non-radioactive 18:1-LPC (0.5 mm) and microsomal fractions (40 μg of protein). Microsomal fractions were from an *ale1*Δ *gpc1*Δ strain overexpressing *GPC1(ScGPCAT*) or from an *ale1*Δ strain expressing EV. LPC constituted 98–99% and PC constituted 1–2% of the radioactive chloroform-soluble lipids after 40 min. The activity of ScGPCAT during the first 10 min, calculated on the formed radiolabeled LPC, was 9.5 nmol/min/mg protein. Corresponding incubations with microsomes prepared from *ale1*Δ *gpc1*Δ transformed with empty vector gave no incorporation of radioactivity into the chloroform phase, indicated with a *star* at 40 min of incubation in the *graph*. Data are given for triplicate assays ± S.D. (*error bars*).

**FIGURE 12.**
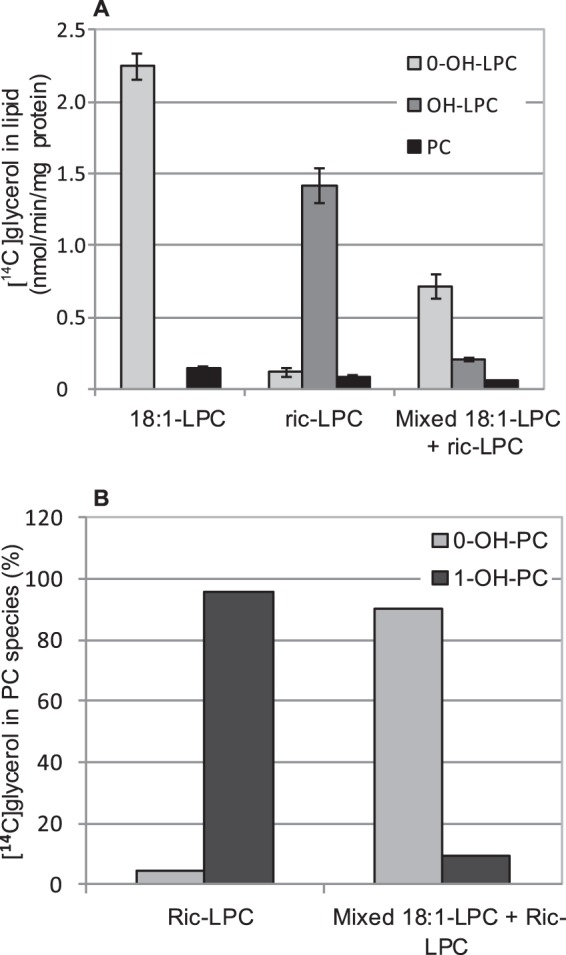
**LPC as donor in transacylation activity.** Radioactive lipids formed in incubations of microsomes (40 μg of protein) prepared from *ale1*Δ *gpc1*Δ expressing ScGPCAT. [^14^C]Glycerol-labeled GPC (0.2 mm) and non-radioactive 18:1-LPC or ricinoleoyl-LPC (*ric-LPC*) (0.1 mm) or an equimolar mixture of both (0.05 + 0.05 mm) for 30 min. Incubations with microsomes prepared from *ale1*Δ *gpc1*Δ strain transformed with empty vector gave no incorporation of radioactivity into the chloroform phase. *A*, results shown are from triplicate assays ± S.D. (*error bars*). *B*, relative distribution of molecular species of PC formed. PC from triplicate assays (*A*) were pooled before phospholipase C treatment to obtain DAG for separation of molecular species (see “Experimental Procedures”). *0-OH-X* and *1-OH-X*, lipids with no ricinoleoyl groups and one ricinoleoyl group, respectively.

Alternative mechanisms could explain the observed incorporation of radioactivity from [^14^C]choline-GPC into LPC and PC. The simplest explanation is transfer of an acyl group from membrane lipids to the added GPC. Among possible acyl donors for such acylation, we tested non-radioactive LPC in combination with [^14^C]choline-labeled GPC and found that membranes derived from *ale1*Δ*gpc1*Δ bearing *GAL1-GPC1* rapidly formed radioactive LPC, whereas membranes from cells transformed with empty vector totally lacked this capacity (Fig. 11). When corresponding incubations were performed with membranes prepared from *ale1*Δ cells expressing EV (containing an endogenous *GPC1* gene), radioactive LPC accumulated at about 10% of the rate that occurs when *GPC1* is overexpressed with the *GAL1-GPC1* plasmid (Fig. 11). This demonstrates that the transacylation rate is a function of the level of GPCAT activity when the LPC concentration is not rate-limiting, as it was in the experiment presented in Figs. 9 and 10. The rate of radioactivity appearing in LPC during the first 10 min with *GAL1-GPC1* was as high as 9.5 nmol/min/mg protein, which is 11% of the acylation rate with 18:1-CoA at optimal GPC concentration with the same membranes. Apart from direct acyl transfer from added LPC, there could be other explanations for the formation of radioactive LPC in assays in which unlabeled LPC but no acyl-CoA had been provided. By adding non-radioactive ricinoleoyl-LPC, which could be separated from LPC with non-hydroxylated acyl groups by TLC, we demonstrated that the added [^14^C]glycerol-labeled GPC received 90% of its acyl groups from the added LPC (Fig. 12*A*). This experiment gave conclusive evidence that GPCAT catalyzed the transfer of acyl groups from added non-radioactive LPC to [^14^C]GPC, thus forming non-radioactive GPC and ^14^C-labeled LPC molecules.

Some radioactive PC was also formed in these assays (Fig. 12). When the radioactivity in the different PC molecular species was analyzed in incubations with added ricinoleoyl-LPC, species with one ricinoleoyl group had 96% of the activity, with the remainder being [^14^C]PC with no ricinoleoyl groups (Fig. 12*B*). These results show that the second acylation step is not using added ricinoleoyl-LPC in an LPC:LPC transacylase reaction but receives acyl groups from some other lipid(s).

##### Gpc1p Catalyzes the Transacylation of Acyl Groups from Lysophospholipids Other than LPC

**FIGURE 13.**
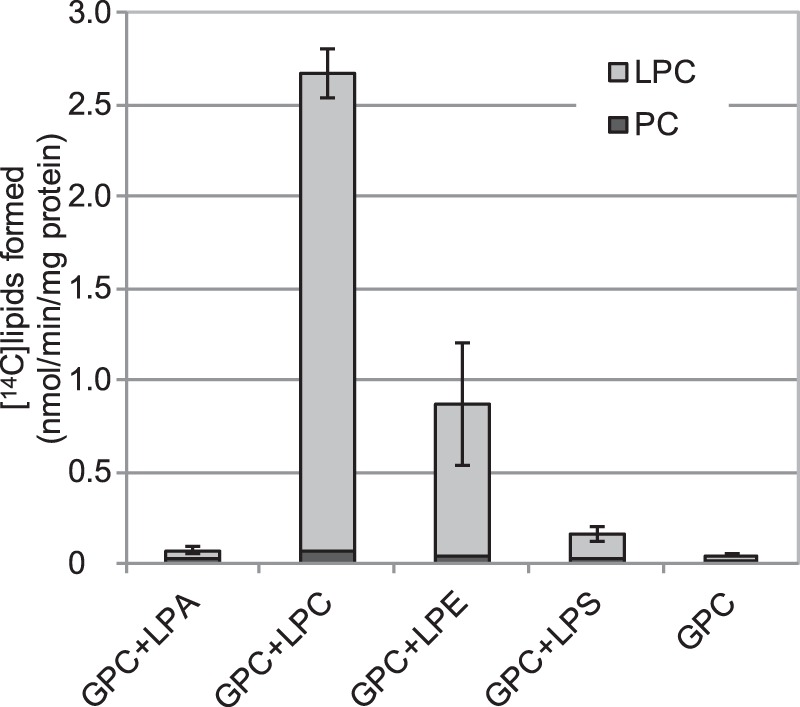
**Lysolipids as acyl donors for ScGPCAT (Gpc1p) activity.** Incorporation of ^14^C activity from [^14^C]choline-labeled GPC (1 mm) into chloroform-soluble lipids in the presence or absence of added (0.4 mm) 18:1-LPA, 18:1-LPC, 18:1-LPE, and 18:1-lysophosphatidylserine (*LPS*) by microsomal preparations (60 μg of protein) prepared from yeast *ale1*Δ *gpc1*Δ cells with overexpressed *GPC1*. Corresponding incubations with empty vector gave no incorporation of radioactivity into the chloroform phase (data not shown). Incubation time was 60 min, and temperature was 30 °C. Data are given for triplicate assays ± S.D. (*error bars*).

We investigated whether lysophospholipids other than LPC could serve as acyl donors for the acylation of GPC by Gpc1p (Fig. 13). LPE could donate acyl groups at about 25% of the efficiency with LPC. Incorporation of radioactivity into LPC from added lysophosphatidylserine was low but significantly higher than in the absence of added lysolipids, whereas no increase was seen with added lysophosphatidic acid (Fig. 13).

##### In Vivo Labeling Indicates a Physiological Role for Gpc1p in PC Biosynthesis

**FIGURE 14.**
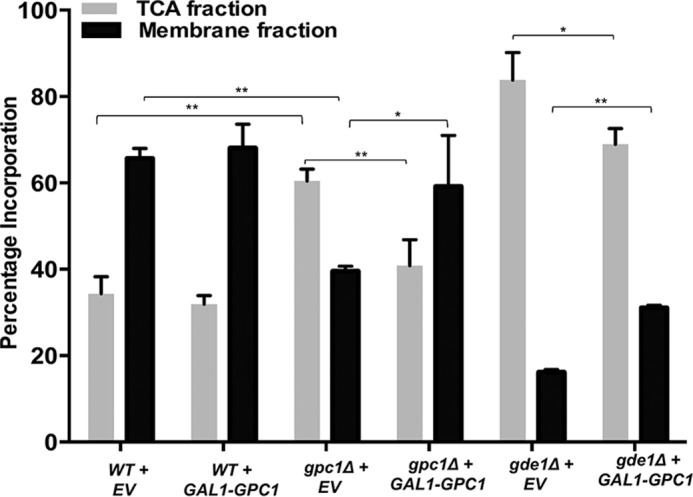
***In vivo* incorporation of [^3^H]GPC.** The indicated strains were grown to logarithmic phase in galactose medium, and the distribution of the radioactivity between the TCA and membrane extracts was determined. Strains contained either EV or *GPC1* under the control of the *GAL1* promoter (*GAL1-GPC1*). Data represent the average of three independent cultures ± S.D. (*error bars*). A *t* test was performed to determine significance as indicated by *brackets* (*, *p* ≤ 0.05*;* **, *p* ≤ 0.0008).

*In vivo* radiolabeling experiments were performed to examine the role of Gpc1p-mediated GPC acylation in cellular PC biosynthesis. For these studies, the medium contained [^3^H]GPC and a low concentration of phosphate to induce transcription of the *GIT1*-encoded permease (11) responsible for the uptake of GPC into the cell (5). Following growth to logarithmic phase, cells were separated into membrane and TCA-extractable fractions, and the percentage of radioactivity in each fraction was determined. As shown in Fig. 14, loss of *GPC1* (compare WT + empty vector (*EV*) with *gpc1*Δ + EV) resulted in less incorporation of [^3^H]GPC radioactivity into the membrane fraction and a concomitant increase in the intracellular fraction. Importantly, the *gpc1*Δ strain containing the *GAL1-GPC1* plasmid displayed a distribution of radioactivity similar to WT. When *GPC1* was overexpressed in the WT strain, there was no change in the distribution of counts as compared with WT containing empty vector. This suggests that expression of *GPC1* from its single genomic copy, as occurs in the WT strain, is not rate-limiting for radioactive flux into the membrane fraction under these conditions. As expected, a *gde1*Δ mutant lacking the glycerophosphodiesterase responsible for GPC hydrolysis (see Fig. 1) (5) exhibited a greater proportion of counts in the internal fraction as labeled GPC (Table 1) as compared with WT (compare WT + EV to *gde1*Δ + EV). In contrast to the WT strain, overexpression of *GPC1* in *gde1*Δ did result in an increase in membrane counts and a decrease in intracellular counts as compared with the empty vector control. This suggests that in the presence of high intracellular levels of substrate (as engendered by loss of Gde1p), *GPC1* expression from the single genomic copy is rate-limiting for incorporation of [^14^]GPC into membrane. In all cases, the radioactivity in the membrane fraction was found only in PC, suggesting that the LPC produced under these conditions is either rapidly converted to PC or deacylated back to GPC. The TCA-extractable fractions consisted of GPC and free choline (Table 1). These results confirm a cellular role for Gpc1p in the synthesis of PC via acylation of GPC.

**TABLE 1:** **Values for intracellular percentages are derived from Fig. 14**

Strain	Choline	GPC	Intracellular percentage
	%	%	%
*WT* + *EV*	14	21	35
*WT* + *GAL1-GPC1*	11	21	32
*gpc1*Δ + *EV*	23 ± 3.2	37 ± 2.1	60
*gpc1*Δ + *GAL1-GPC1*	16 ± 4	25 ± 5.6	41
*gde1*Δ + *EV*	2 ± 0.2	82 ± 8.8	84
*gde1*Δ + *GAL1-GPC1*′	2 ± 0.3	67 ± 9.7	69

## Discussion

Stålberg *et al.* (7) demonstrated that the generally accepted assumption that GPC cannot be acylated and thus could not be a substrate for the synthesis of PC was incorrect. These authors (7) demonstrated acyl-CoA-dependent GPC acylation (GPCAT reaction) in microsomal fractions from yeast, and later Lager *et al.* (8) showed the same enzyme reaction in a microsomal fraction from plants. Here we report on the cloning and characterization of the GPCAT-encoding genes from yeast and plants. The GPCAT genes have not been annotated for any function in yeast or plant databases and do not fall into the MBOAT or acyl-CoA:glycerol-3-phosphate acyltransferase GPAT (PlsB type) families of acyltransferases. GPCAT is also unique in that it has, in addition to acyl-CoA acyltransferase activity, LPC:GPC transacylase activity. Our labeling studies employing intact yeast cells demonstrate the *in vivo* relevance of these enzymatic activities. It is remarkable that despite having acyl-CoA acyl transfer and transacylation activity, the GPCAT enzymes lack any recognizable domain present in other such enzymes where the catalytic activity has been established.

As is the case with any enzyme, the gene/enzyme relationship cannot be unequivocally established until the enzyme is purified to homogeneity. Nonetheless, the preponderance of evidence indicates that Gpc1p harbors catalytic activity and is not a regulatory protein or a non-catalytic subunit of a larger complex. First of all, plant GPCAT proteins are functional in yeast, despite having only 25% amino acid identity (see Fig. 6) with Gpc1p. Second, when *GPC1* is overexpressed, the transacylation and acylation activities are 10–40 times higher as compared with the wild type condition. These findings argue against it having a regulatory function or being a subunit of an enzyme for the gene product.

Homologues of GPCAT are widely distributed among eukaryotic organisms, but no homologues were found in the prokaryote clade. Interestingly, the plant clade showed a very low sequence divergence as compared with other eukaryotic groups. A common reason for low sequence divergence is a low substitution rate as a consequence of protein-protein interactions (12). Therefore, we investigated the Membrane-based Interactome Network Database (M.I.N.D.), which contains high confidence data of protein-protein interactions for *Arabidopsis* membrane proteins, based on the split ubiquitin system in yeast (13). Four proteins, of 151 hits, were related to acyl lipid metabolism and found to interact with AtGPCAT, when excluding interactions tested positive in only one of two assays in the primary screen. Three of these hits are associated with sphingolipids (At2g31360, At3g06470, and At1g69640), whereas the fourth hit belongs to the family of oleosin proteins (At2g25890). Further studies are needed to resolve if plant-specific protein-protein interactions are a reason for the low sequence divergence in plants.

The physiological function of GPCAT in yeast and plants remains to be explored. At face value, GPCAT activity may be part of a recycling pathway to restore PC levels following PC turnover via deacylating phospholipases. On a more complex level, GPCAT has been suggested to be involved in various aspects of acyl editing and phospholipid remodeling in yeast and plants (14–16). Overall, it can be assumed that PC homeostasis is maintained by the concerted action of GPCAT, lysophospholipid lipases, and lysophosphatidylcholine acyltransferases (LPCATs), in addition to the major pathways of PC biosynthesis (the CDP-choline and methylation pathways). It should be noted that GPC is believed to only be formed via deacylation of PC, and if so, GPCAT cannot contribute to any net synthesis of PC if GPC is not provided to the cell from outside. Because plasma membrane GPC transporters are found in yeast (5, 17) and fungi (18), exogenous GPC could, however, under certain environmental conditions contribute to net synthesis of PC via GPCAT in these cells. In all cells, GPCATs would contribute to an acyl editing, whereby acyl groups from the acyl-CoA pool can be inserted into the s*n*-1 position of PC via LPC formation from GPC. Because we show that GPCAT also can acylate GPE with acyl-CoA at about the 25% of the rate of GPC, it could also be involved in acyl editing of PE, similar to what we proposed for PC editing.

Thus, GPCAT-catalyzed acylation of GPC by acyl-CoA can be predicted to have a role in maintaining PC homoeostasis and in acyl editing of this phospholipid. We have now shown that the incorporation of [^14^C]GPC into lipids observed in plant microsomal membranes in the absence of acyl-CoA (8) is due to GPCAT-catalyzed transacylation of acyl groups from LPC to GPC. Because this reaction does not form any new lipid molecular species, it might function as a GPC transporter across intracellular membrane and compartments and/or catalyze the movement of LPC from one leaflet of the membrane to the other. GPCAT can also transfer acyl groups from LPE to GPC in forming GPE and LPC, albeit at a lower rate than if LPC and GPC were substrates. Thus, GPCAT could also be involved in exchanging acyl groups between PC and PE.

We show here that the [^14^C]LPC formed from [^14^C]GPC by transacylation from added LPC was to some extent further converted to [^14^C]PC and that this transfer did not involve LPC:LPC transacylation (LPCT), at least not with LPC containing ricinoleoyl moiety. LPCT reactions were demonstrated in microsomal preparations from developing safflower seeds (8). The enzyme(s) responsible for the formation of PC from LPC in the yeast microsomes remains to be established.

The strategy we used for identifying the GPCAT gene is an example of how the function of a non-annotated gene can be established. Our approach differs from most current methods for identifying the function of genes, which primarily rely on forward and reverse genetics and require an observable phenotype or extrapolation by homology to genes with *a priori* known function. More than 20% of all genes encoding putative proteins in any eukaryote are still not reliably annotated with experimentally defined functions (19), and their functions are not accessible via forward or reverse genetics because silencing or overexpression do not yield obvious phenotypes. We predict that discovery of many new functions and novel enzyme reactions will require renewed emphasis on classical biochemical methods coupled with the increasingly powerful molecular biology and high throughput technologies.

The identification of the GPCAT genes now allows for determining their physiological functions by forward and reverse genetics in the plethora of eukaryotic clades where homologues are present, and such work is now in progress in our laboratories.

## Experimental Procedures

### 

#### 

##### Chemicals

[1-^14^C]Radioactive fatty acids, [^14^C]choline, and [^14^C]glycerol 3-phosphate were purchased from PerkinElmer Life Sciences, and [^14^C]ethanolamine was from American Radiochemicals. Non-radioactive fatty acids and CoA were obtained from Larodan (Malmö, Sweden). Di-ricinoleoyl-PC was kindly provided by ENI/Metapontum Agrobios (Metaponto, Italy). Ricinoleoyl-LPC was produced by phospholipase A_2_ (from *Naja naja*; Sigma) treatment of di-ricinoleoyl-PC. All other non-radioactive fine chemicals were obtained from Sigma. Acyl-CoAs were prepared according to the method described by Sanchez *et al.* (20). [^14^C]Choline-labeled GPC and [^14^C]ethanolamine labeled GPE were obtained by growing WT yeast cells (SCY62) with [^14^C]choline or [^14^C]ethanolamine and purifying the labeled PC or PE and deacylating it according to Lager *et al.* (8). The specific activity of the [^14^C]GPC varied between 40,000 dpm/nmol and 86,000 dpm/nmol, whereas that of [^14^C]GPE was only about 200 dpm/nmol. [^14^C]Glycerol-labeled GPC was prepared by incubating microsomal preparations from developing safflower seeds with 18:1-CoA and [^14^C]G3P according to Guan *et al.* (21). After extracting the lipids from the assay into chloroform (22), the formed [^14^C]glycerol-labeled PC was separated by TLC and deacylated as above. The specific activity of the [^14^C]glycerol-labeled GPC varied between 26,000 and 53,000 dpm/nmol.

##### Yeast Strains, Plasmids, and Microsomal Preparations

The yeast strains used are from the yeast knock-out *Mat a* collection (Thermo Scientific) in the background BY4741 (see Table 2). The double yeast mutant (*ale1*Δ *gpc1*Δ) was obtained by one-step PCR-mediated gene disruption using the *gpc1*Δ strain as background. The *ALE1* gene was replaced by the LEU marker (primers used: 5′-ATGTACAATCCTGTGGACGCTGTTTTAACAAAGATAATTACTGTGCGGTATTTCACACCG-3′ and5′-CTACTCTTCCTTTTTTGAAATAGGCTTTGGTGAGTAACCAGATTGTACTGAGAGTGCAC-3′).

**TABLE 2:** **Yeast strains used in this study**

Strain	Genotype
BY4741	*Mat a, his3*Δ*1, leu2*Δ*0, met15*Δ*0, ura3*Δ*0*
*gpc1*Δ	*Mat a, his3*Δ*1, leu2*Δ*0, met15*Δ*0, ura3*Δ*0, gpc1*::*KANMX*
*ale1*Δ	*Mat a, his3*Δ*1, leu2*Δ*0, met15*Δ*0, ura3*Δ*0, ale1*::*KANMX*
*ale1*Δ *gpc1*Δ	*Mat a, his3*Δ*1, met15*Δ*0, ura3*Δ*0, ale1*::*LEU2, gpc1*::*KANMX*
*gde1*Δ	*Mat a, his3*Δ*1 leu2*Δ*0 met15*Δ*0 ura3*Δ*0, gde1*::*KANMX*

BY4741 was used as WT in GPCAT assays. The genes encoding GPCAT from yeast (*YGR149W*) and *Arabidopsis* (At5g35460) were amplified by PCR, whereas the genes from castor bean (XM_002514040) and oilseed rape (BnaA04g07370D) were ordered synthetically (GenScript) by using known sequence information. The Gateway® system was used to clone into the yeast expression vector pYes-DEST52, which places genes under the control of the *GAL1* promoter (referred to as *GAL1-GPC1* throughout this work). For Western blotting analysis, *GPC1* was cloned into pYES2.1, which contains a C-terminal V5-His_6_ epitope tag (referred to as *GAL1-GPC1-V5* throughout). The vector pYES2 was used as the empty vector control (*EV* in the figures). Microsomal membranes were prepared according to the method described previously (23).

##### Yeast-based Screen for Identifying GPCAT-encoding Gene

The yeast knock-out *Mat a* collection from Thermo Scientific was used for the screen. The deletion strains were transferred from the glycerol stock to a microplate containing solid YPD, grown for 2–3 days at 30 °C, and then plated on YPD plates and grown for an additional 2–3 days. Individual yeast strains were transferred to 2-ml tubes together with 0.5-mm zirconia/silica beads and 0.1 m phosphate buffer, pH 7.2, and disrupted (8 times for 30 s each) using a Mini Beadbeater-8 (Biospec Products). After centrifugation, 20 μl of the yeast extract was incubated with 100 nmol of [^14^C]GPC, 100 nmol of [^14^C]G3P, 1 mg of BSA, 20 mm EDTA, and 20 nmol of 18:1-CoA for 20 min at 30 °C. The reactions were terminated by 110 μl of MeOH/CHCl_3_/HAc (50:50:1, v/v/v), vortexed, and centrifuged. The chloroform phase was applied to a TLC plate (Silica 60, Merck), and the lipids were separated in CHCl_3_/MeOH/HAc/H_2_O (90:20:20:3, v/v/v/v). The radiolabeled lipids were visualized with an Instant Imager (Packard Instrument Co.) electronic autoradiograph.

##### Sequence Alignment and Phylogenetic Tree

Database searches were carried out with the *Arabidopsis* and yeast GPCAT protein as queries using BLASTp and tBLASTn against the GenBank^TM^ database at the National Center for Biotechnology Information. Proteins were selected for inclusion based on phylogenetic distribution of the species. Complementary searches were carried out in genome databases for *Klebsormidium flaccidum* and for *Liriodendron tulipifera*, *Aristolochia fimbriata*, and *Nuphar advena*. All hits used had *e* values of <10^−11^. Back-searches by BLASTp against *A. thaliana* and yeast GPCAT were carried out to verify homology. The selected sequences were also analyzed by BLink (NCBI) to exclude contaminants. Alignments were made using the T-Coffee PSI/TM-Coffee (24) web service, unless otherwise indicated in the figure legends. Alignments were subjected to maximum likelihood analysis in MEGA version 6.0.6 (25), using an 85% limit for partial gaps. Bootstrap testing was conducted with 1000 replicates.

##### Microsomal Enzyme Assay

The enzyme assays were performed with microsomal membranes prepared from recombinant yeast cells and yeast transformed with empty vector, as indicated in the figure legends. Microsomal membranes and radioactive and non-radioactive substrates were incubated in mixtures at concentrations and times stated in the figure legends in 0.1 m phosphate buffer, pH 7.2, with 1% of BSA (essentially fatty acid-free) in a final volume of 50 μl at 30 °C with shaking (250 rpm). LPC was added to the assay mixture dissolved in phosphate buffer, pH 7.2.

##### Lipid Extraction, Separation, and Analysis

The microsomal assays were terminated by addition of 170 μl of 0.15 m acetic acid and 500 μl of CHCl_3_/MeOH (1:1, v/v) and vortexed. After centrifugation, the chloroform phase was removed, and an aliquot was taken for liquid scintillation counting of the radioactivity. The remaining chloroform phase from the extraction of the assays, after removal of an aliquot for liquid scintillation, was applied on a TLC plate (Silica 60, Merck), and the plate was developed in CHCl_3_/MeOH/HAc/H_2_O (85:15:10:3.5, v/v/v/v). Radioactive spots were visualized and identified by *R_f_* values of authentic standards, and the relative amount of radioactivity in each spot was determined by an Instant Imager (Canberra Packard Instrument Co.) electronic autoradiograph. Absolute amounts of radioactivity in each spot were calculated from the total amount of radioactivity in the chloroform phase as determined by liquid scintillation. In the experiments where the molecular species of radioactive LPC and PC were determined, the lipid extracts from the assays were separated on TLC twice with CHCl_3_/MeOH/HAc/H_2_O (85:15:10:3.5, v/v/v/v). This separated ricinoleoyl-LPC from LPC with non-hydroxylated acyl groups, and the relative proportions of radioactivity between these LPC species were determined by autoradiography. The PC species with hydroxylated acyl groups and no hydroxylated acyl groups were not clearly separated, and these PC species were eluted together from the gel and treated with PLC (from *Clostridium perfringens*; Sigma). The resulting diacylglycerol species were then separated on TLC developed with hexane/diethyl ether/acetic acid (70:140:3, v/v/v). The relative radioactivities between diacylglycerols with no, one, and two ricinoleoyl groups were then determined by autoradiography.

##### In Vivo Labeling and Fraction Isolation

*WT* (BY4741), *gpc1*Δ, and *gde1*Δ strains contained either empty vector (pYES 2.1) or *GAL1-GPC1 (*pYES 2.1*-YGR149w).* Cultures were maintained aerobically at 30 °C in yeast nitrogen base (YNB) medium containing 2% galactose and altered to lack inositol and contain a low level of KH_2_PO_4_ (200 μm) to induce expression of the Git1 transporter for GPC uptake. Amino acid concentrations were as described (26), but uracil was removed to maintain the plasmids. For labeling experiments, the medium was supplemented with 5 μm of [^3^H]choline-GPC and grown to log phase before harvesting. A membrane fraction and TCA-extractable intracellular fraction were isolated as described previously (27). The percentages of radioactive counts in each fraction were determined using a liquid scintillation counter.

##### Analysis of Choline-containing Metabolites and Lipids following in Vivo Labeling

The water-soluble choline-containing metabolites were separated using anion exchange chromatography as described previously (27). Standards were used to verify the separation procedure, and label incorporated into each metabolite was quantified using liquid scintillation counting. Lipids were extracted from the membrane fraction as described previously (27) and separated by TLC as described for the *in vitro* assays.

##### Western Blotting

Protein extraction and quantification were performed as described (11). Protein (75 μg) was loaded into each lane of a Mini-PROTEIN 4–15% gel (Bio-Rad). The semidry transfer was performed onto a PVDF membrane using TRANS-BLOT SD (Bio-Rad). Membrane was blocked in blocking buffer (5% milk in Tris-buffered saline, 0.1% Tween 20 solution (TBST) for 60 min at room temperature. Primary antibody was used against V5 epitope (Monoclonal V5 epitope tag antibody (Invitrogen, catalogue no. R960-25) at a dilution of 1:1000 in TBST containing 5% BSA and incubated at 4 °C for overnight. The primary antibody was then removed, and the membrane was washed three times with TBST for 10 min each. The goat anti-mouse secondary antibody (LI-COR Biosciences, catalogue no. 926-322-10) was used at a dilution of 1:10,000 in TBST containing 5% milk. Membranes were incubated in secondary antibody for 60 min at room temperature. Secondary antibody was removed, and the membrane was washed three times with TBST for 10 min each. Finally, the membrane was visualized using the Odyssey FC imaging system. Glucose-6-phosphate dehydrogenase (G6PD) was used as the loading control. The same procedure as above was employed for G6PD; primary antibody was anti-G6PD (Sigma-Aldrich, catalogue no. HPA000-834), and secondary antibody was goat anti-rabbit (LI-COR Biosciences, catalogue no. 926-68071).

## Author Contributions

B. G., J. P.-V., I. L., and S. S. designed research; B. G., J. P.-V., I. L., S. S., M. B., and S. A. carried out the experimental work; M.-S. H. and A. R. did the phylogenetic analyses; S. S. and I. L. wrote the manuscript; and B. G, J. P.-V., S. A., M.-S. H., A. B., and A. R. read and edited the manuscript.

## Supplementary Material

Supplemental Data

## Footnotes

3 The yeast gene name *GPC1* has been approved by the *Saccharomyces* genome database (SGD).

## Footnotes

2 The abbreviations used are:

PC: phosphatidylcholine
GPCAT: glycerophosphocholine acyltransferase
PE: phosphatidylethanolamine
LPC: lysophosphatidylcholine
G3P: glycerol-3-phosphate
LPA: lysophosphatidic acid
PA: phosphatidic acid
GPE: glycerophosphoethanolamine
LPE: lysophosphatidylethanolamine
LPCAT: lysophosphatidylcholine acyltransferase
LPCT: LPC:LPC transacylation
TLC: thin layer chromatography
GPC: glycero-3-phosphocholine
EV: empty vector
G6PD: glucose-6-phosphate dehydrogenase.
