# Supplementary material for: Cloning of Glycerophosphocholine Acyltransferase (GPCAT) from Fungi and Plants: A NOVEL ENZYME IN PHOSPHATIDYLCHOLINE SYNTHESIS
Source: J Biol Chem. 2016 Oct 7;291(48):25066–76. doi: 10.1074/jbc.M116.743062 (PMC5122774; doi:10.1074/jbc.M116.743062)
Supplement: Supplemental Data [file supp_291_48_25066__index.html]

Cloning of glycerophosphocholine acyltransferase (GPCAT) from fungi and plants; a novel enzyme in phosphatidylcholine synthesis — Cloning of Glycerophosphocholine Acyltransferase (GPCAT) from Fungi and Plants — Cloning and Characterization of GPCATs — Supplemental Data 

# Cloning of Glycerophosphocholine Acyltransferase (GPCAT) from Fungi and Plants

## Supplemental Data

- Supplemental figure S1 (.pdf, 646 KB) - Protein alignment used for phylogenetic analysis
