# Supplementary material for: Cloning of Glycerophosphocholine Acyltransferase (GPCAT) from Fungi and Plants: A NOVEL ENZYME IN PHOSPHATIDYLCHOLINE SYNTHESIS
Source: J Biol Chem. 2016 Oct 7;291(48):25066–76. doi: 10.1074/jbc.M116.743062 (PMC5122774; doi:10.1074/jbc.M116.743062)
Supplement: Supplemental Data [file 10.1074_M116.743062_jbc.M116.743062-1.pdf]

```
PpaGPCATh1    1 K-------------------------------------------------------------------------------
PpaGPCATh2    1 K-------------------------------------------------------------------------------
SmoGPCATh1    1 MAPDDDAG------------------------------------------------------------------------
SmoGPCATh2    1 VQ------------------------------------------------------------------------------
ZmaGPCATh1    1 MASEVEDD------------------------------------------------------------------------
ZmaGPCATh2    1 MASEVEDE------------------------------------------------------------------------
OsaGPCATh     1 MASEVEDD------------------------------------------------------------------------
MacGPCATh1    1 MGDGETGS------------------------------------------------------------------------
StuGPCATh1    1 MSSNEESM------------------------------------------------------------------------
StuGPCATh2    1 MEEYYNGD------------------------------------------------------------------------
RcoGPCAT      1 MSNNEDPI------------------------------------------------------------------------
AthGPCAT      1 MANNEDSN------------------------------------------------------------------------
BvuGPCATh     1 MNNDSSEFHINILHHQLPF-------------------------------------------------------------
NnuGPCATh     1 MSNKEDAE------------------------------------------------------------------------
EguGPCATh     1 MSEGEAGL------------------------------------------------------------------------
NadGPCATh     1 GWFTLKKRT-----------------------------------------------------------------------
AtrGPCATh     1 MESYESP-------------------------------------------------------------------------
LtuGPCATh     1 GEKKKGRRK--LLKNRP---------------------------------------------------------------
AfiGPCATh     1 NPARIQHSE-----------------------------------------------------------------------
KflGPCATh     1 MGLILEAA------------------------------------------------------------------------
EmuGPCATh     1 MVLAPLQNKS----------------------------------------------------------------------
OviGPCATh     1 MAPSIANII-----------------------------------------------------------------------
SpuGPCATh     1 MSLQNGLNGCSLKQ------------------------------------------------------------------
CgiGPCATh     1 MRDTLKENTKQFATA-----------------------------------------------------------------
LgiGPCATh1    1 MMVDKEDAKRKTQN------------------------------------------------------------------
LgiGPCATh2    1 MTSQSMNYNQNILIV-----------------------------------------------------------------
LcoGPCATh     1 MAASMTKPPPELADSH-----S-MSS------------------------------------------------------
MciGPCATh1    1 MTPKEDTPGDSDLQDT-----T-VAAAAA-E-------------------------------------------------
MciGPCATh2    1 MLTEERLEHLSSHESD-----A----------------------------------------------------------
MveGPCATh     1 MVTSSIDGPTHNPSST-----M-SS-SDS---DSD--VPLTRSISAPDPD-EHTYMDANATD------------PAWISE
UmaGPCATh     1 MRRASSTSSTYSHVSD-----V-SS-SGD---ALR--PTLSRASSTACSD-----DGSLSTPSFRTHHSSASASME----
LbiGPCATh     1 MWHSTLVFADT---------------------------------------------------------------------
SpoGPCATh     1 MDHLEFDENTDSEYSI-----F-E--------------------------------------------------------
SceGPCAT      1 MYKLDNNDIDDETN------------------------------------------------------------------
LthGPCATh     1 MSDIPDTLDTDSDEGS----------------------------------------------------------------
PkuGPCATh     1 MDIDNMEKDNGCSCTP-----T-SDES-----------------------------------------------------
NcrGPCATh     1 MADLAEPEISLPVSGL-----S-HKSEGAPSTDGE--AILTPDTPSTPPLNSSPGEHDPNTPT------------LDVTT
CbaGPCATh     1 MSEPIRIPLSKTDTTL-----D-EM--AS-----H--DIAGSASTDTAGS-LTGALTPGYQSG------------YQSSS
AclGPCATh     1 MEGAGLDDHPGGGRTA-----Q-VTGAGN-----S--PLLPPAAVDDMDA-VYDTDEYAPSST------------RSYSP
CglGPCATh     1 MADSEDRSKPISIPGN-----N-NRWEGA-STDSE--AILTPELSGSPPD-DDSHEHAVVTPT------------LGPTV
BbaGPCATh     1 MDDEQPAAVSHPNPNA-----T-FQDSIS-D---E--HHTEPGLTSPLSS-SPPDPTILDTPS------------LIATT
DdiGPCATh1    1 MEVLASEISSNEKSAF----------------------------------------------------------------
DdiGPCATh2    1 MEKKDKNEKIISSSPI-----N-IFRRYS-I-------------------------------------------------
AsuGPCATh     1 MTMTMMVIKVEKHSKV-----G----------------------------------------------------------
EdiGPCATh     1 MSTSSEEKYQREFQ------------------------------------------------------------------
AcaGPCATh1    1 MDHKKVDKHEHNDSRA-----G----------------------------------------------------------
AcaGPCATh2    1 MADAVHNEPEADIRAY----------------------------------------------------------------
PbrGPCATh     1 LQQTSADLAYVGTTRL--RRVYRVASADC---DPV--V---------------CINKVT-------------------IY
NgrGPCATh1    1 MEKQEEIEITNNNFDS------E---------------------------------------------------------
NgrGPCATh2    1 MSQQQTKKKKESKQEK------K---------------------------------------------------------
MbrGPCATh     1 MASESTVPADPAESPL-----G-LSQ------------------------------------------------------
BsaGPCATh     1 MWWEIFGVFGVC--------------------------------------------------------------------
OtaGPCATh     1 MRRTRSVRSDD---------------------------------------------------------------------
MpuGPCATh1    1 MASPIAAAAAT---------------------------------------------------------------------
MpuGPCATh2    1 MSPSFAEP------------------------------------------------------------------------
MspGPCATh1    1 MAEAVMRRRRR---------------------------------------------------------------------
MspGPCATh2    1 MNLEPPTL------------------------------------------------------------------------
CreGPCATh     1 MESFGVAAAEAA--------------------------------------------------------------------
CowGPCATh     1 MPANEAPLSPSINATT------ATSPATT---ETTTAAATNTNPSGAAAN-GVQDGGKAQ-------------------T
CmeGPCATh     1 MDDGGRALGSASVDHF-----D-ET-------------------------------------------------------
```

```
PpaGPCATh1    2 --------------------------------------------------------------------------------
PpaGPCATh2    2 --------------------------------------------------------------------------------
SmoGPCATh1    9 --------------------------------------------------------------------------------
SmoGPCATh2    3 --------------------------------------------------------------------------------
ZmaGPCATh1    9 --------------------------------------------------------------------------------
ZmaGPCATh2    9 --------------------------------------------------------------------------------
OsaGPCATh     9 --------------------------------------------------------------------------------
MacGPCATh1    9 --------------------------------------------------------------------------------
StuGPCATh1    9 --------------------------------------------------------------------------------
StuGPCATh2    9 --------------------------------------------------------------------------------
RcoGPCAT      9 --------------------------------------------------------------------------------
AthGPCAT      9 --------------------------------------------------------------------------------
BvuGPCATh    20 ----------------------------------------------------------IE--------------------
NnuGPCATh     9 --------------------------------------------------------------------------------
EguGPCATh     9 --------------------------------------------------------------------------------
NadGPCATh    10 --------------------------------------------------------------------------------
AtrGPCATh     8 --------------------------------------------------------------------------------
LtuGPCATh    16 --------------------------------------------------------------------------------
AfiGPCATh    10 --------------------------------------------------------------------------------
KflGPCATh     9 --------------------------------------------------------------------------------
EmuGPCATh    11 --------------------------------------------------------------------------------
OviGPCATh    10 --------------------------------------------------------------------------------
SpuGPCATh    15 --------------------------------------------------------------------------------
CgiGPCATh    16 --------------------------------------------------------------------------------
LgiGPCATh1   15 --------------------------------------------------------------------------------
LgiGPCATh2   16 --------------------------------------------------------------------------------
LcoGPCATh    21 ----------------------------------------------------------GS--------------------
MciGPCATh1   25 ----------------------------------------------------------TA--------------------
MciGPCATh2   18 -----------------------------------------------------------S--------------------
MveGPCATh    56 PTTP--------------------------SLGPTS--------------------LVPN--------------------
UmaGPCATh    60 --------------------------------------------------------------------------------
LbiGPCATh    12 --------------------------------------------------------------------------------
SpoGPCATh    19 --------------------------------------------------------------------------------
SceGPCAT     15 --------------------------------------------------------------------------------
LthGPCATh    17 --------------------------------------------------------------------------------
PkuGPCATh    22 --------------------------------------------------------------------------------
NcrGPCATh    61 PKSP-------------------------AVLS------------------------RNS--------------------
CbaGPCATh    53 PGSP-------------------------YLLS------------------------RNP--------------------
AclGPCATh    55 TASP-------------------------PSLS------------------------RNH--------------------
CglGPCATh    59 PTSP--------------------------RLS------------------------RNP--------------------
BbaGPCATh    56 PKSP--------------------------KLS------------------------RHP--------------------
DdiGPCATh1   17 ------------------------------------------------------TEEEDDYYEFSDSETDSL--------
DdiGPCATh2   25 ----------------------------------------------------------AD--------------------
AsuGPCATh    18 --------------------------------------------------------------------------------
EdiGPCATh    15 --------------------------------------------------------------------------------
AcaGPCATh1   18 --------------------------------------------------------------------------------
AcaGPCATh2   17 ------------------------------------------------------EPQAADA-------------------
PbrGPCATh    40 PAS----------------------------AAPKG--------------------IFVW------MPLNRA----RRRG
NgrGPCATh1   18 --------------------------------------------ERMDQRMDQGKEGMADHYGSS---------------
NgrGPCATh2   18 --------------------------------------------QEQDP-------------------------------
MbrGPCATh    21 ----------------------------------------------------------ES--------------------
BsaGPCATh    13 ------------------LVVYAAVTRFTTLAAITQMGSIQ---------------------------------------
OtaGPCATh    12 -------------------------------ATVDE--------------------L------------SRARERAK---
MpuGPCATh1   12 -------------------------------AAVTG--------------------LV-----------RRM----RRR-
MpuGPCATh2    9 --------------------------------------------------------------------------------
MspGPCATh1   12 -------------------------------GE-----------------------------------------------
MspGPCATh2    9 --------------------------------------------------------------------------------
CreGPCATh    13 --------------------------------------------------------------------------------
CowGPCATh    52 ---SSTSENAVQTMERPRSAL---------------LRPGQEEQEQAEREAHQTTHQLHDGYAAA---------------
CmeGPCATh    20 -----------------------------------------------------------D--------------------
```

```
PpaGPCATh1    2 --------------------------------------------------------------------------------
PpaGPCATh2    2 --------------------------------------------------------------------------------
SmoGPCATh1    9 --------------------------------------------------------------------------------
SmoGPCATh2    3 --------------------------------------------------------------------------------
ZmaGPCATh1    9 --------------------------------------------------------------------------------
ZmaGPCATh2    9 --------------------------------------------------------------------------------
OsaGPCATh     9 --------------------------------------------------------------------------------
MacGPCATh1    9 --------------------------------------------------------------------------------
StuGPCATh1    9 --------------------------------------------------------------------------------
StuGPCATh2    9 --------------------------------------------------------------------------------
RcoGPCAT      9 --------------------------------------------------------------------------------
AthGPCAT      9 --------------------------------------------------------------------------------
BvuGPCATh    22 ----------------------------VR--E----------------------------------QI-LKSLLFQSI-
NnuGPCATh     9 --------------------------------------------------------------------------------
EguGPCATh     9 --------------------------------------------------------------------------------
NadGPCATh    10 --------------------------------------------------------------------------------
AtrGPCATh     8 --------------------------------------------------------------------------------
LtuGPCATh    16 --------------------------------------------------------------------------------
AfiGPCATh    10 --------------------------------------------------------------------------------
KflGPCATh     9 --------------------------------------------------------------------------------
EmuGPCATh    11 --------------------------------------------------------------------------------
OviGPCATh    10 --------------------------------------------------------------------------------
SpuGPCATh    15 --------------------------------------------------------------------------------
CgiGPCATh    16 --------------------------------------------------------------------------------
LgiGPCATh1   15 --------------------------------------------------------------------------------
LgiGPCATh2   16 ------------------------------------------------------------------KP------------
LcoGPCATh    23 ---------------SS---NG-DD---LS-DF-----------------------------EHDLDVI-------EGW-
MciGPCATh1   27 ---------------SS---SG------IK-NF-------------------------G---NEDMFSL-------DEW-
MciGPCATh2   19 ---------------TL---LS-ED---ST-EY-------------------------D---TDELTTV-------DSW-
MveGPCATh    70 ----------------------------------------------------------ILTQLSFS--------------
UmaGPCATh    60 ----------------------------------------------------------GIDLLGSSASA-------AAL-
LbiGPCATh    12 --------------------------------------------------------------------------------
SpoGPCATh    19 -----------------------------E-DN-------------------------D---YGLHGLD-------DSV-
SceGPCAT     15 ------------------------------------------------------------------NSV-------SLT-
LthGPCATh    17 ----------------------------------------------------------------EPMKV-------TLA-
PkuGPCATh    22 ---------------SS----------NAYADL-------------------------S---SSDAPFF-------EGV-
NcrGPCATh    72 ---------------SF---SG--SISSTL-HD-------------------------E---WDVFPPL-------DRL-
CbaGPCATh    64 ---------------SH---IG--S--QSL-QE-------------------------D---W--EVPL-------DKL-
AclGPCATh    66 ---------------S-----------FSY-QD-------------------------D---WDTFPPL-------DKL-
CglGPCATh    69 ---------------SF---SG--S--SSY-QE-------------------------D---WDPFPPL-------DRL-
BbaGPCATh    66 ---------------SF---SG--S--SSY-HD-------------------------D---WDSLPPL-------DRL-
DdiGPCATh1   35 --------------DYN---LE----DSSN-D-ENSGDGDGDGGVVESDDNKNLKRRKR---SKRPTTL-------GESN
DdiGPCATh2   27 ---------------VG---SGSSISRLQH-DS-------------------------N---NN------------NSP-
AsuGPCATh    18 -----------------------ELSPSSR-RL-------------------------K---DK------------SKD-
EdiGPCATh    15 --------------------------------------------------------------------------------
AcaGPCATh1   18 -----------------------EQ---VE-EL-------------------------R---DENPSTV-------SED-
AcaGPCATh2   24 ---------------QH---LH----DESE-EQHHKSQNDS---------------------------------------
PbrGPCATh    62 SADERMTE-DEVGSASDVA----T---DSE-ED-------------------------D---ER-----------SASE-
NgrGPCATh1   39 --------------------------------------------------------------------------------
NgrGPCATh2   23 --------------------------------------------------------------------------------
MbrGPCATh    23 ---------------A-------------S-Q-----------------------------------VI-------EDI-
BsaGPCATh    36 ---------------------------------ERAMPWA----------------------------------------
OtaGPCATh    26 --------------------------------------------------------------------------------
MpuGPCATh1   25 ---DALSDDDENGA-TTSSDEG-V---GS---P-------------------------R---GH----VVAHHA------
MpuGPCATh2    9 --------------------------------------------------------------------------------
MspGPCATh1   14 ----------ENGY-MSPTSDG-E---DS---P-------------------------R---EW----RR---RNPLAP-
MspGPCATh2    9 --------------------------------------------------------------------------------
CreGPCATh    13 --------------------------------------------------------------------------------
CowGPCATh    99 ---------------------------------ESEQDHA----------------------------------------
CmeGPCATh    21 ---------------SS---AF-PD---SE-AY-------------------------------YEPDL-------LSL-
```

```
PpaGPCATh1     2 --------------------------------------------------------------------------------
PpaGPCATh2     2 --------------------------------------------------------------------------------
SmoGPCATh1     9 --------------------------------------------------------------------------------
SmoGPCATh2     3 --------------------------------------------------------------------------------
ZmaGPCATh1     9 --------------------------------------------------------------------------------
ZmaGPCATh2     9 --------------------------------------------------------------------------------
OsaGPCATh      9 --------------------------------------------------------------------------------
MacGPCATh1     9 --------------------------------------------------------------------------------
StuGPCATh1     9 --------------------------------------------------------------------------------
StuGPCATh2     9 --------------------------------------------------------------------------------
RcoGPCAT       9 --------------------------------------------------------------------------------
AthGPCAT       9 --------------------------------------------------------------------------------
BvuGPCATh     36 --------------------------------------------------------------------------------
NnuGPCATh      9 --------------------------------------------------------------------------------
EguGPCATh      9 --------------------------------------------------------------------------------
NadGPCATh     10 --------------------------------------------------------------------------------
AtrGPCATh      8 --------------------------------------------------------------------------------
LtuGPCATh     16 --------------------------------------------------------------------------------
AfiGPCATh     10 --------------------------------------------------------------------------------
KflGPCATh      9 --------------------------------------------------------------------------------
EmuGPCATh     11 --------------------------------------------------------------------------------
OviGPCATh     10 --------------------------------------------------------------------------------
SpuGPCATh     15 --------------------------------------------------------------------------------
CgiGPCATh     16 --------------------------------------------------------------------------------
LgiGPCATh1    15 --------------------------------------------------------------------------------
LgiGPCATh2    18 --------------------------------------------------------------------------------
LcoGPCATh     43 -------------------------------------------------TTADFISE-----------------------
MciGPCATh1    46 -------------------------------------------------ETSDFISE-----------------------
MciGPCATh2    40 -------------------------------------------------SSHDFVAK-----------------------
MveGPCATh     78 --------------------------------------------------------------------------------
UmaGPCATh     74 --------------------------------------------------------------------------------
LbiGPCATh     12 --------------------------------------------------------------------------------
SpoGPCATh     33 --------------------------------------------------------------------------------
SceGPCAT      21 --------------------------------------------------------------------------------
LthGPCATh     25 --------------------------------------------------------------------------------
PkuGPCATh     41 --------------------------------------------------------------------------------
NcrGPCATh     95 --------------------------------------------------------------------------------
CbaGPCATh     83 --------------------------------------------------------------------------------
AclGPCATh     83 --------------------------------------------------------------------------------
CglGPCATh     90 --------------------------------------------------------------------------------
BbaGPCATh     87 --------------------------------------------------------------------------------
DdiGPCATh1    82 KDGETANSVSISAGTSKSNISSPLVDGADILSKPNSSPTSSSLSGSNSAN--GFLSDDDDLLLNGNEQLNENHNTNSTNG
DdiGPCATh2    47 --------------------------------------------------LVPTSC------------------------
AsuGPCATh     33 --------------------------------------------------SDKYLD------------------------
EdiGPCATh     15 --------------------------------------------------------------------------------
AcaGPCATh1    35 -------------------------------------------------TNDAFVD------------------------
AcaGPCATh2    42 --------------------NATQPERAEDLA------------------------------------------------
PbrGPCATh     93 --------------------------------------------------------------------------------
NgrGPCATh1    39 ----------------------------EDED------------------------------------------------
NgrGPCATh2    23 ----------------------------VTTA------------------------------------------------
MbrGPCATh     31 -------------------------------------------------R--NFMSDL----------------------
BsaGPCATh     43 -----------------------------SFS------------------------------------------------
OtaGPCATh     26 --------------------------------------------------------------------------------
MpuGPCATh1    56 --------------------------------------------------------------------------------
MpuGPCATh2     9 --------------------------------------------------------------------------------
MspGPCATh1    40 --------------------------------------------------------------------------------
MspGPCATh2     9 --------------------------------------------------------------------------------
CreGPCATh     13 --------------------------------------------------------------------------------
CowGPCATh    106 -----------------------------SCD------------------------------------------------
CmeGPCATh     39 --------------------------------------------------------------------------------
```

```
PpaGPCATh1    2 --------------------------------------------------------------------------------
PpaGPCATh2    2 --------------------------------------------------------------------------------
SmoGPCATh1    9 --------------------------------------------------------------------------------
SmoGPCATh2    3 --------------------------------------------------------------------------------
ZmaGPCATh1    9 --------------------------------------------------------------------------------
ZmaGPCATh2    9 --------------------------------------------------------------------------------
OsaGPCATh     9 --------------------------------------------------------------------------------
MacGPCATh1    9 --------------------------------------------------------------------------------
StuGPCATh1    9 --------------------------------------------------------------------------------
StuGPCATh2    9 --------------------------------------------------------------------------------
RcoGPCAT      9 --------------------------------------------------------------------------------
AthGPCAT      9 --------------------------------------------------------------------------------
BvuGPCATh    36 -------------------------SLCKIPSIS----------------------------------------------
NnuGPCATh     9 --------------------------------------------------------------------------------
EguGPCATh     9 --------------------------------------------------------------------------------
NadGPCATh    10 --------------------------------------------------------------------------------
AtrGPCATh     8 --------------------------------------------------------------------------------
LtuGPCATh    16 --------------------------------------------------------------------------------
AfiGPCATh    10 --------------------------------------------------------------------------------
KflGPCATh     9 --------------------------------------------------------------------------------
EmuGPCATh    11 --------------------------------------------------------------------------------
OviGPCATh    10 --------------------------------------------------------------------------------
SpuGPCATh    15 --------------------------------------------------------------------------------
CgiGPCATh    16 --------------------------------------------------------------------------------
LgiGPCATh1   15 --------------------------------------------------------------------------------
LgiGPCATh2   18 --------------------------------------------------------------------------------
LcoGPCATh    51 -------------------------NDFDMI--E----------------------------------------------
MciGPCATh1   54 -------------------------NDFDMI--D----------------------------------------------
MciGPCATh2   48 -------------------------DDYAMI--G----------------------------------------------
MveGPCATh    78 ------------------------------L--D----------------------------------------------
UmaGPCATh    74 -------------------------PLSDLF--E----------------------------------------------
LbiGPCATh    12 ------------------------------F--E----------------------------------------------
SpoGPCATh    33 -------------------------GFTDLF--D----------------------------------------------
SceGPCAT     21 -------------------------SLLEFL--D----------------------------------------------
LthGPCATh    25 -------------------------NWVEFL--D----------------------------------------------
PkuGPCATh    41 -------------------------TLLDLL--Q----------------------------------------------
NcrGPCATh    95 -------------------------TVLDLL--D----------------------------------------------
CbaGPCATh    83 -------------------------TFFDIF--D----------------------------------------------
AclGPCATh    83 -------------------------TVFDLL--D----------------------------------------------
CglGPCATh    90 -------------------------TVLDIL--D----------------------------------------------
BbaGPCATh    87 -------------------------TVLDLL--D----------------------------------------------
DdiGPCATh1  160 ATNNNNNNNNNSGSNSPSHQQSSEL-------------------------------------------------------
DdiGPCATh2   53 ------------------------------D--S----------------------------------------------
AsuGPCATh    39 ---------------------------------E----------------------------------------------
EdiGPCATh    15 --------------------------------------------------------------------------------
AcaGPCATh1   42 ---------------------------------A----------------------------------------------
AcaGPCATh2   54 -----------------LQIASGKV-------------------------------------------------------
PbrGPCATh    93 -------------------------VFGQIV--D----------------------------------------------
NgrGPCATh1   43 -------------------TLSDQL-------------------------------------------------------
NgrGPCATh2   27 -------------------TITPTT-------------------------------------------------------
MbrGPCATh    38 -------------------------DFATQQ--D----------------------------------------------
BsaGPCATh    46 ----------------------------------------------------------------------KPDA--GGGS
OtaGPCATh    26 --------------------------------------------------------------------------------
MpuGPCATh1   56 --------------------------------------------------------------------------------
MpuGPCATh2    9 --------------------------------------------------------------------------------
MspGPCATh1   40 -------------------------FFK----------------------------------------------------
MspGPCATh2    9 --------------------------------------------------------------------------------
CreGPCATh    13 --------------------------------------------------------------------------------
CowGPCATh   109 -----------------HLLHDDHAALLDAA--DDLLAPGIQLQGSVNDMNDRVESYVMQTLDRIASGLDSDDEDPDGAD
CmeGPCATh    39 -------------------------SLSDIF--T----------------------------------------------
```

```
PpaGPCATh1    2 --------------------------------------------------------------------------------
PpaGPCATh2    2 --------------------------------------------------------------------------------
SmoGPCATh1    9 --------------------------------------------------------------------------------
SmoGPCATh2    3 --------------------------------------------------------------------------------
ZmaGPCATh1    9 --------------------------------------------------------------------------------
ZmaGPCATh2    9 --------------------------------------------------------------------------------
OsaGPCATh     9 --------------------------------------------------------------------------------
MacGPCATh1    9 --------------------------------------------------------------------------------
StuGPCATh1    9 --------------------------------------------------------------------------------
StuGPCATh2    9 --------------------------------------------------------------------------------
RcoGPCAT      9 --------------------------------------------------------------------------------
AthGPCAT      9 --------------------------------------------------------------------------------
BvuGPCATh    45 --------------------------------------------------------------------------------
NnuGPCATh     9 --------------------------------------------------------------------------------
EguGPCATh     9 --------------------------------------------------------------------------------
NadGPCATh    10 --------------------------------------------------------------------------------
AtrGPCATh     8 --------------------------------------------------------------------------------
LtuGPCATh    16 --------------------------------------------------------------------------------
AfiGPCATh    10 --------------------------------------------------------------------------------
KflGPCATh     9 --------------------------------------------------------------------------------
EmuGPCATh    11 --------------------------------------------------------------------------------
OviGPCATh    10 --------------------------------------------------------------------------------
SpuGPCATh    15 --------------------------------------------------------------------------------
CgiGPCATh    16 --------------------------------------------------------------------------------
LgiGPCATh1   15 --------------------------------------------------------------------------------
LgiGPCATh2   18 --------------------------------------------------------------------------------
LcoGPCATh    58 --------------------------------------------------------------------------------
MciGPCATh1   61 --------------------------------------------------------------------------------
MciGPCATh2   55 --------------------------------------------------------------------------------
MveGPCATh    80 --------------------------------------------------------------------------------
UmaGPCATh    81 --------------------------------------------------------------------------------
LbiGPCATh    14 --------------------------------------------------------------------------------
SpoGPCATh    40 --------------------------------------------------------------------------------
SceGPCAT     28 --------------------------------------------------------------------------------
LthGPCATh    32 --------------------------------------------------------------------------------
PkuGPCATh    48 --------------------------------------------------------------------------------
NcrGPCATh   102 --------------------------------------------------------------------------------
CbaGPCATh    90 --------------------------------------------------------------------------------
AclGPCATh    90 --------------------------------------------------------------------------------
CglGPCATh    97 --------------------------------------------------------------------------------
BbaGPCATh    94 --------------------------------------------------------------------------------
DdiGPCATh1  185 ------PLLNLFQKFLNREAI-----------------------------------------------------------
DdiGPCATh2   55 --------------------------------------------------------------------------------
AsuGPCATh    40 --------------------------------------------------------------------------------
EdiGPCATh    15 --------------------------------------------------------------------------------
AcaGPCATh1   43 --------------------------------------------------------------------------------
AcaGPCATh2   62 ------SKKSFFERFTVLWAQ-----------------------------------------------------------
PbrGPCATh   100 --------------------------------------------------------------------------------
NgrGPCATh1   49 ------VVEDEEE-YAYY-DW-----------------------------------------------------------
NgrGPCATh2   33 ------TTT--------T-NN-----------------------------------------------------------
MbrGPCATh    45 --------------------------------------------------------------------------------
BsaGPCATh    54 GHRSTT-------------------------------NTTATSSQQPDKATS------------------------SEAT
OtaGPCATh    26 --------------------------------------------------------------------------------
MpuGPCATh1   56 --------------------------------------------------------------------------------
MpuGPCATh2    9 --------------------------------------------------------------------------------
MspGPCATh1   43 --------------------------------------------------------------------------------
MspGPCATh2    9 --------------------------------------------------------------------------------
CreGPCATh    13 AE------------------------------------------------------------------------------
CowGPCATh   170 AVRDSSSHSDNPDNLQFYQQHDNEQTDRDASSIRRQQEKRASKRSVPSGADSDSEWVMSPVGSDARDLSDMDAGFTPSAS
CmeGPCATh    46 --------------------------------------------------------------------------------
```

```
PpaGPCATh1    2 --------------------------------------------------------------------------------
PpaGPCATh2    2 --------------------------------------------------------------------------------
SmoGPCATh1    9 --------------------------------------------------------------------------------
SmoGPCATh2    3 --------------------------------------------------------------------------------
ZmaGPCATh1    9 --------------------------------------------------------------------------------
ZmaGPCATh2    9 --------------------------------------------------------------------------------
OsaGPCATh     9 --------------------------------------------------------------------------------
MacGPCATh1    9 --------------------------------------------------------------------------------
StuGPCATh1    9 --------------------------------------------------------------------------------
StuGPCATh2    9 --------------------------------------------------------------------------------
RcoGPCAT      9 --------------------------------------------------------------------------------
AthGPCAT      9 --------------------------------------------------------------------------------
BvuGPCATh    45 ------------------------SLSLPHAPLLHEKAIK---MS--------------------NSDD----------E
NnuGPCATh     9 --------------------------------------------------------------------------------
EguGPCATh     9 --------------------------------------------------------------------------------
NadGPCATh    10 --------------------------------------CR---IITKEKEKFG-----------EKMGQ----------E
AtrGPCATh     8 --------------------------------------------------------------------------------
LtuGPCATh    16 --------------------------------------KRRKKK-----------KK--------GMSSESEEEAIGDDE
AfiGPCATh    10 --------------------------------------S---HV--------GKKEKNPENKSREKMSK----------E
KflGPCATh     9 --------------------------------------------------------------------------------
EmuGPCATh    11 --------------------------------------------------------------------------------
OviGPCATh    10 --------------------------------------------------------------------------------
SpuGPCATh    15 --------------------------------------------------------------------------------
CgiGPCATh    16 --------------------------------------------------------------------------------
LgiGPCATh1   15 --------------------------------------------------------------------------------
LgiGPCATh2   18 --------------------------------------------------------------------------------
LcoGPCATh    58 ------------------------ML--S---------------------------------------------------
MciGPCATh1   61 ------------------------ML--S---------------------------------------------------
MciGPCATh2   55 ------------------------VL--S---------------------------------------------------
MveGPCATh    80 ------------------------SF-IA---------------------------------------------------
UmaGPCATh    81 ------------------------TY-FS---------------------------------------------------
LbiGPCATh    14 ------------------------TY-FD---------------------------------------------------
SpoGPCATh    40 ------------------------APNIY---------------------------------------------------
SceGPCAT     28 ------------------------PIASK---------------------------------------------------
LthGPCATh    32 ------------------------PLSFT---------------------------------------------------
PkuGPCATh    48 ------------------------PTTLN---------------------------------------------------
NcrGPCATh   102 ------------------------NFALP---------------------------------------------------
CbaGPCATh    90 ------------------------NLALP---------------------------------------------------
AclGPCATh    90 ------------------------NLSLS---------------------------------------------------
CglGPCATh    97 ------------------------NFALS---------------------------------------------------
BbaGPCATh    94 ------------------------NFALP---------------------------------------------------
DdiGPCATh1  200 --------------------------------------------------------------------------------
DdiGPCATh2   55 --------------------------------------------------------------------------------
AsuGPCATh    40 ------------------------ILSPS---------------------------------------------------
EdiGPCATh    15 --------------------------------------------------------------------------------
AcaGPCATh1   43 ------------------------NLALM---------------------------------------------------
AcaGPCATh2   77 --------------------------------------------------------------------------------
PbrGPCATh   100 ------------------------VFLRE---------------------------------------------------
NgrGPCATh1   62 --------------------------------------------------------------------------------
NgrGPCATh2   39 --------------------------------------------------------------------------------
MbrGPCATh    45 ------------------------NFG-----------------------------------------------------
BsaGPCATh    79 --------------------------------------------------------------------------------
OtaGPCATh    26 --------------------------------------------------------------------------------
MpuGPCATh1   56 --------------------------------------------------------------------------------
MpuGPCATh2    9 --------------------------------------------------------------------------------
MspGPCATh1   43 --------------------------------------------------------------------------------
MspGPCATh2    9 --------------------------------------------------------------------------------
CreGPCATh    15 --------------------------------------------------------------------------------
CowGPCATh   250 SSSEQFDQFVRRVQQGESTVDAFNQSQMP---------------------------------------------------
CmeGPCATh    46 ------------------------LG------------------------------------------------------
```

```
PpaGPCATh1    2 --------------------------------------------------------------------------------
PpaGPCATh2    2 --------------------------------------------------------------------------------
SmoGPCATh1    9 ----SP--------------------------------------------------------------------------
SmoGPCATh2    3 --------------------------------------------------------------------------------
ZmaGPCATh1    9 ----AAVP-----------GA--P-----------------------L---VA---------------------------
ZmaGPCATh2    9 ----AATP-----------L-------------------------------LA---------------------------
OsaGPCATh     9 ----AAAV-----------LT--N---------------------------GG---------------------------
MacGPCATh1    9 ----DEGE-----------AF--QKVRH---RIA-DQS--------KR---VV---------------------------
StuGPCATh1    9 ----EDSN---------GHSF--GKVKQ---RFK-DRS------------------------------------------
StuGPCATh2    9 ----S---------------F--GKVKE---RFK-DRS------------------------------------------
RcoGPCAT      9 ----NEFV-------SNGDSF--EKVKQ---RLK-DRS--------KK---VA---------------------------
AthGPCAT      9 ----SNGL----------DSF--DAVKQ---RFK-DRS--------KK---VV---------------------------
BvuGPCATh    68 KVEEVN-----------GHSF--QKVKQ---RLK-DRT------------------------------------------
NnuGPCATh     9 ----DGGD-----------PF--PTVKQ---RFS-DRT--------KK---V----------------------------
EguGPCATh     9 ----DD-----------GEAF--RKVRQ---RIA-DQS--------KR---VV---------------------------
NadGPCATh    28 EGEDCD-----------AKPF--RKVRQ---KLV----------------------------------------------
AtrGPCATh     8 ---E---------------------AQE---AFR----------------------------------------------
LtuGPCATh    39 IVGGGE-----------KESFR-KAVRK---RLS-DRT--------KK---VA---------------------------
AfiGPCATh    31 EVDGDR-----------GQPF--RRVKQ---RFS-DRT--------KK---VA---------------------------
KflGPCATh     9 ----AEEATARSDASTSDSET--EGLRQ---RRP-GDA--------EV---LA----QEPL------------WV---P-
EmuGPCATh    11 --------------------------------------------------------------------------------
OviGPCATh    10 --------------------------------------------------------------------------------
SpuGPCATh    15 -------------------------LTEVAE---QDDG--------TK--WAQ----PAKD-------------------
CgiGPCATh    16 --------------------------------------------------------------EEE-VTY-----E-----
LgiGPCATh1   15 -------------------------------------------------------------YNTG-TSR-----E-----
LgiGPCATh2   18 -------------------------VDTVVTCT-NDDG--------NTKPVAS----VTINGFIP-RSS-----S-----
LcoGPCATh    61 ------------------AA-L-EQVTH---QLN-DKQ--------RE---IR----AR-----------STEWTTKTK-
MciGPCATh1   64 ------------------AA-L-DQVTN---QLE-DKQ--------RE---FI----SK-----------SSEWTNKTR-
MciGPCATh2   58 ------------------AA-L-EQVTL---QIR-DVK--------NE---IK----AL----------LSTEWTSMTQ-
MveGPCATh    84 ------------------QN-L-EEELT---DLP-VLANQIYKTLRQD---LK----KNKQ---K-LKHQM-------K-
UmaGPCATh    85 ------------------PR-I-DLAGR---KWS-AFS----GDMRSR---AR----RRRE---E-LVRKA---------
LbiGPCATh    18 ------------------SR-V-DLLQR---KLQ-KHS--------DR---LK----MKAE---E-TFKIK-------E-
SpoGPCATh    45 ------------------RV-Y-SWLHK---HYN-QKK--------GQ---LKHGVSRQKN---K-L-------------
SceGPCAT     33 ------------------VV-S-KYYHG---SHLSKAE--------QK---LR----NFEG---F-RRRKPHHEHD-SH-
LthGPCATh    37 ------------------AQ-S-QYLPR---KYLTRAR--------RR---LK----S-------------ARPRQ-KL-
PkuGPCATh    53 ------------------LE-L-RKKKD---DTYKWVR--------KV---YN----DK---------------------
NcrGPCATh   107 ------------------QQ-L-EKLQR---GIH-ART--------EK---MR----KSGE---A-LKEKSAIARERMV-
CbaGPCATh    95 ------------------AK-L-EKWQA---TLQ-AQK--------EK---LA----RQQE---R-IRSSGNNAKDRAM-
AclGPCATh    95 ------------------QK-L-EKWQH---AIN-VQK--------EK---VR----KQRE---K-LKYTSMHAKDRVV-
CglGPCATh   102 ------------------QQ-L-EKLQK---GIS-AQT--------EK---VR----KSRQ---A-FGNKSRIARERMV-
BbaGPCATh    99 ------------------QQ-L-EKLQK---GIS-AQT--------DK---VR----RSRD---A-LKSKTQTARHRMV-
DdiGPCATh1  200 -----------------------KVLQQ---RHS-AIN--------NR---LQ----R---------------L-KKVVD
DdiGPCATh2   55 -------------------P-T-SNFK-----------------------------------------------------
AsuGPCATh    45 ------------------SP-I-GQFKK---SID-DRR------------------------------------------
EdiGPCATh    15 --------------------------------------------------------------------------------
AcaGPCATh1   48 ------------------RE-L-DALAD---ALQ-SLP--------V-----------------------------GFK-
AcaGPCATh2   77 -----------------------RKMID---KLK-T-S--------PH---VK----R----------------------
PbrGPCATh   105 ------------------VR-L-ENLQT---TLE-TG--------------------R-GA---GLVRD--------AR-
NgrGPCATh1   62 -----------------------ETVGS---SLQ-NQE--------EE---LI----DLWM----RINDAFSDRKKSLVN
NgrGPCATh2   39 -------------------------------------N--------NT---TT----TTSS----SP-----------KQ
MbrGPCATh    48 ------------------AAPLSVNMMR---RLT-IL-----------------------------------------R-
BsaGPCATh    79 --------------------------------------------------------------------------------
OtaGPCATh    26 ------------------------AVAR---AIR-RK----------------------SG---ELLKS--------AK-
MpuGPCATh1   56 -----------------------RGVGQ---FFK-RP----------------------VE---NV--------------
MpuGPCATh2    9 ----PNKV-----------A------------------------------------------------------------
MspGPCATh1   43 -----------------------RPRRP---SLP-EG----------------------AR---AI--------------
MspGPCATh2    9 ----RSSE-----------E------------------------------------------------------------
CreGPCATh    15 --------------------------------------------------------------------------------
CowGPCATh   279 ------------------RS-R-RPSAA---HLD-RES--------EQ---AR----RLQK----SMSTSSI--GDRLID
CmeGPCATh    48 -------------------S-L----------------------------------------------------------
```

```
PpaGPCATh1    2 ----------------------------------------------------------------------------TK-E
PpaGPCATh2    2 ----------------------------------------------------------------------------TK-E
SmoGPCATh1   11 ----------------------------KLAAA-----------RR---------------------------SEVAA-R
SmoGPCATh2    3 ----------------------------------------------------------------------------TK-K
ZmaGPCATh1   19 --------------------------NGAADVR-----------RR---------------------------RDQAK-A
ZmaGPCATh2   16 --------------------------NGAADVR-----------RR---------------------------RDQAK-A
OsaGPCATh    18 --------------------------AGAAEVR-----------RR---------------------------RDQAK-E
MacGPCATh1   30 --------------------------QTKEILS-----------KQ---------------------------AAQTK-E
StuGPCATh1   28 --------------------------------Q-----------KT---------------------------KEKTK-Q
StuGPCATh2   22 --------------------------------K-----------KT---------------------------KERTK-Q
RcoGPCAT     34 --------------------------QTKEILS-----------KQ---------------------------ANQTK-E
AthGPCAT     31 --------------------------QTRELLS-----------KQ---------------------------AVQTR-E
BvuGPCATh    89 -----------------------------KRVS-----------ET---------------------------KEKTK-K
NnuGPCATh    29 ---------------------------TKEIIS-----------KQ---------------------------AVQTK-E
EguGPCATh    30 --------------------------QTKEILSKQAV-------QT---------------------------KEKTK-E
NadGPCATh    46 --------------------------FHRVKGL-----------KT---------------------------VVQTK-E
AtrGPCATh    15 --------------------------RARIRLM-----------AK---------------------------KAKTK-E
LtuGPCATh    65 --------------------------QTKEILS-----------KQ---------------------------AVQTK-E
AfiGPCATh    56 --------------------------QTRELLSKQAVQTKEILSKQ---------------------------AVQTK-E
KflGPCATh    48 -PRR---------------------VNSRELVA-----------KH---------------------------VKKIR-M
EmuGPCATh    11 -------------I-----------Y-------------------------------------------------RST-L
OviGPCATh    10 --------------------------------------------------------------------------------
SpuGPCATh    34 --V----------I-----------R-------------------------------------------------DSG-S
CgiGPCATh    23 -SSGPQVKPHQWVE-----------ARN---TN-----------G-------------------------K--LHKLK-S
LgiGPCATh1   23 -SG----------V-----------EKHIKKMS-----------D-------------------------R--MKQVK-A
LgiGPCATh2   49 -IG----------I------------EPTKSTL-----------S-------------------------K--LEITE-S
LcoGPCATh    90 -EK----------L-----------RRQTARLE-----------D-------------------------Q--KTRLQ-A
MciGPCATh1   93 -DS----------I-----------KTQTKKLE-----------D-------------------------Q--RSRIQ-E
MciGPCATh2   88 -GR----------L-----------RKQKILLE-----------A-------------------------R--KAMIQ-D
MveGPCATh   121 -HQ----------M-----------GSTKAQFD-----------K-------------------------Q--RGHLK-S
UmaGPCATh   117 --R----------S-----------KRELQQMD-----------D-------------------------E--LTKMR-L
LbiGPCATh    47 -LS----------G-----------DALAENLE-----------R-------------------------E--IKNLK-L
SpoGPCATh    73 -------------------------QPIHKQIN-----------Y-------------------------E--TDKLK-E
SceGPCAT     69 -HP----------H-----------HLNRSRSF-----------L-------------------------Q--LEDFK-V
LthGPCATh    64 -NQ----------L-----------KTTAQDLT-----------P-------------------------Q--LEDFK-R
PkuGPCATh    74 --K----------K-----------SANINLSK-----------E-------------------------D--LKEIK-R
NcrGPCATh   143 -DE----------W-----------RKRVPTAE-----------E-------------------------Q--LERYK-K
CbaGPCATh   131 -AE----------W-----------RKRVPPAD-----------E-------------------------Q--LAKYR-S
AclGPCATh   131 -GE----------W-----------KRRVPTAD-----------E-------------------------Q--LDKYR-H
CglGPCATh   138 -DE----------W-----------RRRVPSAE-----------E-------------------------Q--LDRYR-K
BbaGPCATh   135 -EE----------W-----------RRRVPSAD-----------E-------------------------Q--FDRYR-K
DdiGPCATh1  222 PKK----------L-----------RERGKILK-----------NEKEKI---------------------------K-S
DdiGPCATh2   61 -RR----------V-----------AESK-IVK-----------K-------------------------LKVLEFVN-E
AsuGPCATh    59 -HR----------F-----------VDQK-MLA-----------K-------------------------LKVLEFVN-S
EdiGPCATh    15 ----------------------------RQYIS-----------RLMDKH--------------------------GL-S
AcaGPCATh1   66 -EK----------M-----------DSH---------------------------------------------REKLA-H
AcaGPCATh2   92 ---------------------------ATEFVG-----------SGTNKLRERLI---------------------NQ-Q
PbrGPCATh   128 -KH----------I--------------QDQIR-----------R-------------------------Q--RHKIR-D
NgrGPCATh1   96 --K----------F-----------QGISNNLK-----------RELENQ---------------------------K-L
NgrGPCATh2   52 --G----------P-----------QQATPKPS-----------STLVNV---------------------------S-S
MbrGPCATh    64 -KR----------L-----------ATSQDKMR------QRLSSN-------------------------N--LNSLRRK
BsaGPCATh    79 -------------------------------------------------------SSSSK--------------------
OtaGPCATh    44 -----------------------------EETS-----------R-------------------------R-GKSAVR-R
MpuGPCATh1   70 --------------------------------G-----------R-------------------------A-AKRRLR-A
MpuGPCATh2   14 --------------------------RTLCA--------------P---------------------------AEV---A
MspGPCATh1   57 --------------------------------P-----------E-------------------------G-ARNFVR-A
MspGPCATh2   14 --------------------------E----------------------------------------------------N
CreGPCATh    15 --------------------------------------------------------------------------------
CowGPCATh   314 --K----------FSEIRDKGGDVRSAVRHLVA-----------HEKERLQHLISKHRDRIRERQLELAHH--QAKVK-A
CmeGPCATh    50 -----------------------------ANLD-----------D-------------------------K--IRNTK-H
```

```
PpaGPCATh1    5 LI---TK--Q-A-------TV---------IA--KR-A--DEHEDFLTKVVT----------------------------
PpaGPCATh2    5 II---KK--Q-A-------TV---------IA--KS-A--DEHEDFIAK-IM----------------------------
SmoGPCATh1   24 FL---SE--R-A-------SI---------IS--RR-A--DEHEQLLNK-VT----------------------------
SmoGPCATh2    6 IL---SK--H-A-------GK---------LA--TR-A--DKHEKFINK-VK----------------------------
ZmaGPCATh1   34 IL---SK--Q-A-------VK---------IA--TK-A--EQHERFIFK-VT----------------------------
ZmaGPCATh2   31 IL---ST--Q-A-------VR---------IA--TK-A--EQHERFIFK-VT----------------------------
OsaGPCATh    33 IL---SK--Q-A-------VK---------IA--TK-A--EEHERFIFK-VT----------------------------
MacGPCATh1   45 IL---SK--Q-A-------VK---------IA--KQ-A--EEHERIIFK-VT----------------------------
StuGPCATh1   37 IL---SK--Q-A-------AQ---------IA--KR-A--EEHERFINK-VT----------------------------
StuGPCATh2   31 ML---SK--Q-A-------VK---------IA--QR-A--EEHEPFINK-VT----------------------------
RcoGPCAT     49 IL---SK--Q-A-------VK---------IA--KQ-A--EEHESFINK-VT----------------------------
AthGPCAT     46 IL---SK--Q-A-------VK---------IA--KQ-A--EEHERFINK-VT----------------------------
BvuGPCATh   101 II---SE--H-A-------ER---------IA--KQ-A--EEHERFINK-VT----------------------------
NnuGPCATh    43 II---SK--Q-A-------VK---------IA--RQ-A--EEHERFINK-VT----------------------------
EguGPCATh    49 IL---SK--Q-A-------VK---------IA--KQ-A--EEHERILFK-VT----------------------------
NadGPCATh    61 IL---SK--Q-A-------KR---------IA--KQ-A--EEHERFINK-VT----------------------------
AtrGPCATh    30 IL---SK--Q-A-------VK---------LA--KQ-A--EEHERFINK-VT----------------------------
LtuGPCATh    80 IL---SK--Q-A-------VK---------IA--KQ-A--EEHERFINK-VT----------------------------
AfiGPCATh    82 IL---SK--Q-A-------VK---------IA--KQ-A--EEHERFINK-VT----------------------------
KflGPCATh    67 EA---AK--N-V-------EK---------IQ--HH-T--EEHDRFYTK-LT----------------------------
EmuGPCATh    17 KL--M-S--T-T---------------------ERK-R--LIPPVLINK-IT----------------------------
OviGPCATh    10 -----------------------------------------GCRMFSEK-LC----------------------------
SpuGPCATh    41 VA--E-H--R-LNEIEQPYHEVQAVESKPQLG-CKD-R--LNKSNCPEK-MV----------------------------
CgiGPCATh    49 VLLVLIM--EES--V-QSSVPYKQLEAG-SKTNALK-E--WKKSRIIEK-FV----------------------------
LgiGPCATh1   42 HL---SL--E-E-------QD---------LREFLL-V--LKKKRLQEK-LV----------------------------
LgiGPCATh2   67 GL---SQ--EEE-------SE---------LAAFLS-L--LKRKRLQEK-LV----------------------------
LcoGPCATh   109 QL---LK--K-Y-------DA---------IEVRMNRD--AKTVRLRDK-IS----------------------------
MciGPCATh1  112 LL---VR--Q-Y-------HK---------IDKRMNRD--AKTVQLRDK-VS----------------------------
MciGPCATh2  107 RL---LN--Q-Y-------GT---------IRTRMHRD--ANAVRLHDK-VS----------------------------
MveGPCATh   140 QI---DT--S-L-------AV---------WEKNMQ-K--ASVVRFMDK-VA----------------------------
UmaGPCATh   135 RV---SK--R-I-------EN---------LQQKWM-D--AKVVRLRDK-IS----------------------------
LbiGPCATh    66 KV---ST--R-M-------AT---------LSASWQ-S--AKVVRTREK-VS----------------------------
SpoGPCATh    89 RL---GK--S-I-------DK---------FQEQWN-S--GKVVRFRDK-LS----------------------------
SceGPCAT     88 RA---LQ--R-I-------RN---------LDKPLD-SIFFKNSSRLEK-AF----------------------------
LthGPCATh    83 RT---IE--R-L-------HS---------LDRPLE-SLFFHNSSRLEK-WF----------------------------
PkuGPCATh    92 FL---HI--K-L-------DQ---------VYLKVN-E--TEKASSTEK-LF----------------------------
NcrGPCATh   162 RM---RV--N-V-------DK---------LGKRWN-D--TKVITLREK-VA----------------------------
CbaGPCATh   150 RM---KK--S-V-------DD---------LSKRWN-E--VVTISIREK-VS----------------------------
AclGPCATh   150 RM---KD--G-V-------KR---------LEKQWN-A--TATVTLREK-IS----------------------------
CglGPCATh   157 RM---RT--S-V-------EK---------LGSRWN-D--TKAITLREK-IS----------------------------
BbaGPCATh   154 RM---RQ--R-V-------DK---------LGRQWN-D--TKAISLREK-IS----------------------------
DdiGPCATh1  242 LI---KL--K-K-------VQ---------LDDHMN-A--PPFIRLLDK-CA----------------------------
DdiGPCATh2   81 KR---EE--F-Q-------RD---------LYKDGR-A--SQLIRTKDK-FS----------------------------
AsuGPCATh    79 RR---EE--F-E-------Q----------LLLKKG-A--SDLLRTGDK-LA----------------------------
EdiGPCATh    29 FI---DK--Y-Q-------NK---------LNKLME-S--EPFLKTLDK-VS----------------------------
AcaGPCATh1   78 KL---ET--R-L-------KS---------VKDGMQ-R--APFIKTRDK-FS----------------------------
AcaGPCATh2  112 YI---DK--K-K-------KE---------LKEKMN-M--PPFVRTIDK-LG----------------------------
PbrGPCATh   144 KI---LQ--S-R-------LA---------WQKKTL-D--PPFCRFIDK-MS----------------------------
NgrGPCATh1  114 QL---KQ--K-K-------ET---------LVREMN-Q--PENVKTKDK-YA----------------------------
NgrGPCATh2   70 SL---RK--R-Y-------KI---------LKNL---P--NFSVSFSDK-IA----------------------------
MbrGPCATh    89 MI---TG-NP-H-------KR---------VRDFVK-E--EPVVKTIDK-FS----------------------------
BsaGPCATh    84 ------------------------------FDSQGG-K--LQDVRTKDK-VV----------------------------
OtaGPCATh    57 AV---TG--SAE-------TR---------VRDFVK-I--PRVIRQIDK-VS----------------------------
MpuGPCATh1   80 RV---TGDPTGE-------TR---------VREYVK-L--PRTIQLIDK-LS----------------------------
MpuGPCATh2   24 GL---RR--A-L-------DI---------VRATRD-V--YKTQQGLSQ-LT----------------------------
MspGPCATh1   67 KL---TG--DPR-------VR---------VRDYVK-M--PRTIQTIDK-VS----------------------------
MspGPCATh2   16 GL---RR--A-L-------EI---------VKKQGD-F--YKRQQGLSP-LS----------------------------
CreGPCATh    15 -------------------------------------L--EAAEAAEGK-ADGMAAVVGASGGAEPGGAASDGADFGSES
CowGPCATh   368 DF---DR--R-R-------QA---------WEERVS-K--PPFLRLIDK-IC----------------------------
CmeGPCATh    62 RL---VE--R-R-------RA---------IERRVR-T--PEVIRLRDK-LS----------------------------
                                                  |Start of DUF2838
```

```
PpaGPCATh1   30 ------------YCIGVVSFGTFCYL------LGSKPADIPKLYC-LFFITVAPLRWIYYRIKKWHYYL-----------
PpaGPCATh2   29 ------------YCIGVVSFGTFCFL------LGSRPDAIPKLYC-LFFITVAPLRWIYYRMKKWHYYF-----------
SmoGPCATh1   48 ------------YCIGVFCFGTVCYV------MGSRPHEIPYLYC-LFFLGIAPLRWIYYRTRKWHYYL-----------
SmoGPCATh2   30 ------------YCLGVFCFGTFCYL------LGSRPQDIPYLYC-TFFITVAPLRWIYYRIRKWHYYL-----------
ZmaGPCATh1   58 ------------HLIGVLGFGGFCYL------LGARPQDVPYVYC-LFYVIFVPLRWIYYRYKKWHYYL-----------
ZmaGPCATh2   55 ------------HLIGVLGFGGFCYL------LGARPQDVPYVYC-LFYVIFVPLRWIYYRYKKWHYYL-----------
OsaGPCATh    57 ------------HLLGVLGFGTFCYL------LGARPQDVPYVYC-LFYVIFVPLRWIYYRYKKWHYYLLFSYGYYNREE
MacGPCATh1   69 ------------HLIGVLGFGAFCFI------LGARPQDVPYVYC-FFYVTFVPLRWIYYRYKKWHYYL-----------
StuGPCATh1   61 ------------HLMGVLGFGAFCFI------LGARPKDIRYVYC-LFYVIFVPLRWIYYRYKKWHYYL-----------
StuGPCATh2   55 ------------HLIGVLGFGAFCFI------LGARPQDIRYAYS-FFYLIFVPLRWIYYRFKKWHYYL-----------
RcoGPCAT     73 ------------HLLGVLGFGGFCFL------LGARPQDIPYVYC-LFFFIFVPLRWIYYRFKKWHYFL-----------
AthGPCAT     70 ------------HLVGVLGFGGFCFL------LGARPQDIPLVYC-FFYVIFVPLRWIYYRFKKWHYYL-----------
BvuGPCATh   125 ------------YLCGVLGFGGFCFL------LGARPQDVPYVYC-LFYVIFVPLRWIYYKYKKWHYYL-----------
NnuGPCATh    67 ------------HLIGVLGFGAFCFL------LGSRPQDVPYVYC-FFYVIFVPLRWIYYRFKKWHYYL-----------
EguGPCATh    73 ------------HLMGVLGFGAFCFI------LGARPQDVPYVYC-LFYVTFVPLRWIYYRYKKWHYYL-----------
NadGPCATh    85 ------------HLLGVLGFGAFCFL------LGSRPQDIPYVYC-VFYVTFVPLRWIYYRFKKWHYYL-----------
AtrGPCATh    54 ------------HLIGVLGFGAFCFL------LGSRPQDIPYIYC-LFYVTFVPLRWIYYRFMKWHYYL-----------
LtuGPCATh   104 ------------HLIGVLGFGAICFL------LGARPQDIPYVYC-LFFVTFVPLRWIYYRFKKLHYYL-----------
AfiGPCATh   106 ------------HLIGVLGFGSFCFL------LGARPQDIPYVYC-LFFVTFVPLRWIYYRFKKWHYFL-----------
KflGPCATh    91 ------------YSLGVLCFGLFCFL------VGAKPQVIPYFYC-FFLFVALPLRWMYYKAKKWHYFL-----------
EmuGPCATh    38 ------------FVISVTLLTVFVFC------AVFSPPALFLILT-ILFVILMIFRVIAYTRSNNILFM-----------
OviGPCATh    20 ------------FVCATAFIVLSTYF------FVVRPALILYIFS-GFFVVAMVFRVINYWRSNYLLFM-----------
SpuGPCATh    82 ------------FVVSILTVIVVCCS------MMSFQWFLPYLYT-AYLSVLIPIRAYKYTNLKWQFFL-----------
CgiGPCATh    91 ------------YVVAVITIIFLTHA------TVYAQWLLPYYYA-VSTPILILIRVIMYWKNKWQYFL-----------
LgiGPCATh1   68 ------------YVISIGTIILLTHS------LMASQWLLPYYYT-ISTPCLLLIRTIIYWKYKWNYFL-----------
LgiGPCATh2   94 ------------YVISIGTIILLTHS------LMASQWLLPYYYT-ISTPCLLLIRTIIYWKFRWHYFL-----------
LcoGPCATh   136 ------------FVVGVGNACVSPAL------AARLPTWIPLYYT-IQSLYLLSLRYLIYKVKKWHYFI-----------
MciGPCATh1  139 ------------FVVGVGNACVAPAL------ALRYPSLIPSYYS-SQLIVLLILRYVIYRSKRWHYFI-----------
MciGPCATh2  134 ------------FVVGVGNTCITPVI------AARLPVWIPIYYT-VQLCYLITLRFFVYKSKQWHYFF-----------
MveGPCATh   166 ------------FTIGMFECCCTPWL------VAQYPEWIPLVHT-VQVATLIMVRYFLYKKKSWHFFL-----------
UmaGPCATh   161 ------------FVVGVCNLVVSSLV------FALRPELIPTLYS-LLALYFLPLRVWSYTSKKWHYFL-----------
LbiGPCATh    92 ------------FFFGVMSLLLSALL------FGIAPHWVHVAYS-VQGLYLLPLRAYRYKKRAWHYFL-----------
SpoGPCATh   115 ------------FALGVSTCILTALL------VGMAPESMHLWYT-IQLFVYLPLRYYTYQRKGYEYFI-----------
SceGPCAT    116 ------------YPFTLFNIFFIGFL------MGRFPEWFHVYYT-ILFFVLMPIRFYTYYKTKNHYFL-----------
LthGPCATh   111 ------------YPFTLFHIFAIGFI------IGKYPSWFHVYYT-GMLVLLMPVRFYTYYKTNNHYYM-----------
PkuGPCATh   118 ------------FSFSVYMIFFIGYL------IGHSPEYIHILYS-VMFAILMPIRLLTYYKRDYGYFL-----------
NcrGPCATh   188 ------------FIFGVMNIFISGYL------IGAFPEWFHIWYT-VQILYFMPIRFYRYHKQGYHYFL-----------
CbaGPCATh   176 ------------FISAVLNVFLSGYL------VGAVPQYFYWWYT-IQLIYFMPIRAYTYHRKGYHYFL-----------
AclGPCATh   176 ------------FIAGVLNIFISGYL------IGAYPEYFYIWFS-VQLAYFMPIRYYGYHKKGYHYFL-----------
CglGPCATh   183 ------------FICGVMNIFVSGYL------IGGYPEYFHLWYT-IQVLYFMPIRFYTYHKRGYHYFL-----------
BbaGPCATh   180 ------------FICGVMNIFISGYL------IGGYPEYFHLWYT-VQLVYFMPIRMFTYHRRGYHYFL-----------
DdiGPCATh1  268 ------------FTMGLIILMVSEFV------LLKSPQLFYLWYT-VLIFPLMAIRFVMYHREKYHYFM-----------
DdiGPCATh2  107 ------------FVLGVLNLCVISFI------CGKYPLYLQYFFS-GEYLVLFATRIILYKRKKLHYFL-----------
AsuGPCATh   104 ------------FVMGVLNLCIISFV------YGRAPKYIPDVFA-VEFCLGFILRLYSYRKKKQHYFL-----------
EdiGPCATh    55 ------------FVCGVLTLLLTQHI------LSALPQFMPYYYV-ILIFPLLGARYFIYKKNGWQYFM-----------
AcaGPCATh1  104 ------------FVAGVTNVALTFLL------LGRHPEWVPIYYT-LKAFLLLTIRWYVFKGKKYHYFL-----------
AcaGPCATh2  138 ------------FMMGILLMLVTEAL------L-TKPNAMYQFYT-VLMFPLMVFRFASYHRMKWHYFM-----------
PbrGPCATh   170 ------------FTVGILGSLITVCL------LGRSSDLMVKWYT-YLIVLLISLRIYLYRKEGYQYFL-----------
NgrGPCATh1  140 ------------FTIGVLIMVSSTFL------LAKYPYLLPDYYI-VMSVILMVIRFFHYHGMGFHYFM-----------
NgrGPCATh2   94 ------------FTVGVLLLVSTTYL------FGHSPKYLPIWYI-LWMIPLMIYRFIDFHSQHAHYFM-----------
MbrGPCATh   116 ------------FVAGVSTMLALEYF------LLQETRYLPYFYV-ALMFPLLMARWFLYRKAGWQYFL-----------
BsaGPCATh   102 ------------YTLSVINAVLTAFL------FGVVPHYFHWWHS-PKAIMYIAHRWYTFRKQNQHYLL-----------
OtaGPCATh    84 ------------FCVGVFGLMMTEYV------ATRAPEKFWMFYA-FAMPAIVCVRAVYYRMIRWQYFL-----------
MpuGPCATh1  109 ------------FTGGVLGLMLTQHV------ATTRPGLFWLYYL-VVAPFVFANRVATYTAMKYHYFL-----------
MpuGPCATh2   50 ------------FFVGVMNVFLTAFI------LGNTPEYYWVWQL-LKNVAMSTASYVIKYRNHQRLYL-----------
MspGPCATh1   94 ------------FTLGVVGLMLTQFV------ATEHPEYFWFYYL-CSAPLVFIYRIIMYRMIKYHYFL-----------
MspGPCATh2   42 ------------FALGLFNVGWSAFC------LGRFVQYYWVWQV-VKSVMLNYLSYSIKLKNHQRLYL-----------
CreGPCATh    55 GAESEDVVEFLDATDGGPGLVMAEAVLDMHEVFHELPEQLAALQVGLGEAVVTP----------QKRRR-----------
CowGPCATh   394 ------------FTGFVASFLVTEFL------LLRYPQWIWLWYA-VCIIPLVGTRYVLYHREQWHYFL-----------
CmeGPCATh    88 ------------FVLGLLNVLFTEYV------ILRQPARLWQWYL-YQLTPLLILRYYSYRKSAQHYFM-----------
```

```
PpaGPCATh1   80 ---------LDFCYYANAIFVV------ML-LF----------------Y------P-T--ND----KLFLICFSFSEGP
PpaGPCATh2   79 ---------FDFCYYANTIFVV------ML-LY----------------F------P-K--ND----KLFLVCFSFAEGP
SmoGPCATh1   98 ---------LDFCYYANVIFMA------ML-LV----------------F------P-N--NE----KLFMVCFSFSEGP
SmoGPCATh2   80 ---------FDFCYYANAIFMA------ML-LY----------------F------P-K--NE----RLFMVCFAFAEGP
ZmaGPCATh1  108 ---------LDFCYYANTFLLV------MI-LF----------------Y------P-K--DE----KLFMVCFSFAEGP
ZmaGPCATh2  105 ---------LDFCYYANTFLLV------MI-LF----------------Y------P-E--DE----KLFMVCFSFAEGP
OsaGPCATh   118 ALQFQAIDCLDFCYYANTFLLV------MI-LF----------------Y------P-K--DE----KLFMVCFSFAEGP
MacGPCATh1  119 ---------LDFCYYANTFFLV------ML-LF----------------F------P-K--NE----KLFMVCFSFAEGP
StuGPCATh1  111 ---------LDFCYYANTIFLV------TL-LF----------------F------P-T--KE----KLFMVCFSFAEGP
StuGPCATh2  105 ---------LDFCYYANTIFII------ML-LG----------------F------P-T--NE----TFFMVCFSFAEGP
RcoGPCAT    123 ---------LDFCYYANTIFLV------DL-LL----------------Y------P-K--DE----KLFMVCFSFAEGP
AthGPCAT    120 ---------LDFCYYANTIFLV------DL-LL----------------Y------P-K--NE----KLFMVCFSFAEGP
BvuGPCATh   175 ---------LDFCYYANTIFLV------AL-LM----------------Y------P-K--NE----KLFMVCFSFSEGP
NnuGPCATh   117 ---------LDFCYYANTLFLV------ML-IL----------------Y------P-R--NE----KFFMVCFSFAEGP
EguGPCATh   123 ---------LDFCYYANTIFII------VL-LF----------------F------P-K--NE----KLFMVCFSFAEGP
NadGPCATh   135 ---------LDFCYYANTIFLV------ML-LL----------------Y------P-R--NE----KLFMVCFSFAEGP
AtrGPCATh   104 ---------LDFCYYANTIFLF------ML-LL----------------Y------P-K--NE----KLFMVCFSFAEGP
LtuGPCATh   154 ---------LDFCYYANTIFLV------ML-LF----------------Y------P-R--NE----KLFMVCFSFAEGP
AfiGPCATh   156 ---------LDFCYYANTIFLV------ML-LF----------------Y------P-R--NE----KLFMVCFSFAEGP
KflGPCATh   141 ---------LDFCYYANAIFVF------LL-LV----------------Q------P-D--NE----RLFMICFAFSEGP
EmuGPCATh    88 ---------LGPCYFVNVYTLI------FI-WA----------------V------P-D--SR----PVFLVLFGLANST
OviGPCATh    70 ---------LDTCYLINLISLC------LI-WA----------------F------P-T--CH----KLQRIQFGLANAH
SpuGPCATh   132 ---------ADWCYVANAMSIL------FL-WA----------------A------P-Y--KS----NYFMIVFGVTNGP
CgiGPCATh   141 ---------LDFCYYANILWYS------FIWWV----------------V------P-Y--NE----KFFAVVFALSNGP
LgiGPCATh1  118 ---------LDFCYYGNVLWYL------FL-WI----------------W------P-Q--HG----NIFMASFAVANGP
LgiGPCATh2  144 ---------LDFCYYGNILWFA------FI-WI----------------A------P-H--HG----DLFSVAFAIANGP
LcoGPCATh   186 ---------FDLCYFVNAMTLL------FL-WV----------------F------P-G--SR----AMFIATYCLTNGP
MciGPCATh1  189 ---------FDICYFVNAMTML------FL-WA----------------Y------P-T--SK----LLFVASFCMTNGP
MciGPCATh2  184 ---------FDLCYYVNLLTLL------FL-WV----------------F------P-S--ST----LLYTAAFTLTNGP
MveGPCATh   216 ---------LDMCYFVNVAVIL------YL-YV----------------F------P-Q--SQ----ALLGAVWLLSLGP
UmaGPCATh   211 ---------FDFCYFLNVANLL------FI-WV----------------F------P-S--SE----FLFTVCYCAAHGP
LbiGPCATh   142 ---------FDLCYYVTILNFV------YI-WL----------------F------P-S--SP----ALFVACYCLSHGA
SpoGPCATh   165 ---------ADFCYWGNILLLV------YI-WI----------------F------P-E--SR----RLFILSYSISYGT
SceGPCAT    166 ---------ADFCYFVNMLCLL------FI-WI----------------F------P-Y--SY----SLFQSCFAFTFGT
LthGPCATh   161 ---------ADLCYYVNLMVLL------FV-WV----------------W------P-D--SV----HLYQSCFAFTFGT
PkuGPCATh   168 ---------ADLCYYVNYLLLI------YI-WI----------------L------P-D--SQ----SLYIACCSFSWGS
NcrGPCATh   238 ---------ADLCYFVNFLLLL------SL-WV----------------F------P-G--SK----RLFTAVYCLAYGN
CbaGPCATh   226 ---------ADLCYFVNFLLLS------SI-WA----------------F------P-R--SK----RLFISAYCLAYGN
AclGPCATh   226 ---------ADLCYFVNMLCML------SI-WV----------------F------P-R--SK----RLFISTFCLTFGN
CglGPCATh   233 ---------ADLCYFVNFLLML------SI-WA----------------F------P-N--SK----RLFTAVYCLAFGN
BbaGPCATh   230 ---------ADLCYFVNVLLML------SL-WA----------------F------P-S--SK----RLFTAAFCLAFGN
DdiGPCATh1  318 ---------LDFCYLCQILLLF------YL-FG----------------HQYILGEK-V--TP----NLFKLVFALSNGP
DdiGPCATh2  157 ---------FDFCYFANLMLLF------FI-YG----------------L------P-D--RQ----WYFITCFCIANGP
AsuGPCATh   154 ---------FDYCYYANFMLLW------YT-HGPE-------------YV------R-S--ST----VLFKICFSFCNGP
EdiGPCATh   105 ---------IDFCYFCQIITLL------CI-FS----------------L------N-TEYIS----PLFQIAFVFSHGP
AcaGPCATh1  154 ---------FDFCYYANILVLL------YL-HF----------------F------P-K--SE----TMFIICFAFCTGP
AcaGPCATh2  187 ---------LDFCYYCQILLLF------FI-YW----------------H------S-S--NP----IYFQFVFCLSNGP
PbrGPCATh   220 ---------IDFCYFTTGLVYM------YL-YL----------------Y------P-K--SS----TLFVHVFAFANGP
NgrGPCATh1  190 ---------LDFCYLANFILIF------YL-GF----------------Y------T-N--SP----HLFLLNFVNSSGP
NgrGPCATh2  144 ---------IDFCYMGNLALMF------FL-FF----------------F------P-T--SP----HLFVLTFANNCGP
MbrGPCATh   166 ---------ADFCYFLNWLTML------CI-LL----------------P------SVF--GA----TARRISFVMGHGP
BsaGPCATh   152 ---------YDFCYWANGLTLL------YC-WV----------------C------P-W--SD----VMFQILFVCSNGP
OtaGPCATh   134 ---------YDFCYFANVSVLV------FL-WT------------------------R-R--SA----TAFRCIFAFANGP
MpuGPCATh1  159 ---------IDFCYYANAVCFA------QI-LV----------------N------P-G--SA----RLFRTTFAYATGP
MpuGPCATh2  100 ---------LDLCHVLGFFYIV------IA-FAGLLASASPHLHAIFAPF------T-S--SK----LLFLTAFGIANGP
MspGPCATh1  144 ---------IDFCYYANLACFV------HI-LG----------------A------P-T--SG----RLFRSVFAYSNGP
MspGPCATh2   92 ---------MDLCHWLSFAYSIVGSISLAA-WAIP------GLKEGLAPV------T-S--SP----YVFRACFCLANGP
CreGPCATh   114 ---------YDFCYYANLLLVL------QL-WL----------------L------P-R--WA----PLA----------
CowGPCATh   444 ---------LDYCYFAQCLLLF------YL-FF----------------Y------P-T--SG----QLFELVFSSMNGP
CmeGPCATh   138 ---------YDYCYFVQALLVA------FY-FG----------------W------P-R--TQAWRQVMFVALFASANGP
```

```
PpaGPCATh1  115 LAWALIVWRCSLVFSSIDKIISVLIHLLPG----TVFFIIRWWDP-ITFAH-----------------------------
PpaGPCATh2  114 LAWALIVWRCSLVFSSFDKLVSVLIHLLPG----LVFYIIRWWDP-ASFSH-----------------------------
SmoGPCATh1  133 LAWALIVWRCSLVFSSIDKIISVLIHLLPGKFHSTVFFIIRWWDP-ITYPH-----------------------------
SmoGPCATh2  115 LAWALIIWRCSLVFSSIDKIISVLIHLLPG----LVFFIVRWWDP-STFSL-----------------------------
ZmaGPCATh1  143 LAWALIVWRCSLVFGSFDKLVSVLIHLLPG----IVLFTIRWWDP-QTFAA-----------------------------
ZmaGPCATh2  140 LAWALIVWRCSLVFSSFDKLVSVLIHLLPG----IVLFTIRWWNP-QTFAA-----------------------------
OsaGPCATh   162 LAWALIVWRCSLVFSSFDKLVSVLIHLLPG----IVIFTIRWWNP-QTFAA-----------------------------
MacGPCATh1  154 LAWALIVWRNSLVFSSLDKLVSVLIHLFPG----IVFFTIRWWNP-ETFAA-----------------------------
StuGPCATh1  146 LAWALIVWRCSLVFSSVDKIVSVFIHLLPG----LVFFTIRWWDP-EFFGA-----------------------------
StuGPCATh2  140 LAWALIVWRCSLVFSSVDKTVSVFIHLLPG----LVFFTIWWWDP-VFFGA-----------------------------
RcoGPCAT    158 LAWALIVWRCSLVFSSVDKIVSVLIHLLPG----LVFFTIRWWNP-ATFEA-----------------------------
AthGPCAT    155 LAWAIIVWRCSLVFSSPDKIVSVLIHLLPG----LVFFTIRWWNP-ATFAA-----------------------------
BvuGPCATh   210 LAWAIIVWRCSLVFNSIDKLTSVLIHLLPG----LVFFTIRWWNP-STFAA-----------------------------
NnuGPCATh   152 LAWALIVWRCSLVFSSPDKIVSVLIHLLPG----IVFFTIRWWDP-ATFEA-----------------------------
EguGPCATh   158 LAWALIVWRCSLVFGSLDKIVSVLIHLLPG----IVFFTIRWWNP-ETFAA-----------------------------
NadGPCATh   170 LAWALIVWRCSLVFSSVDKIVSVLIHLLPG----LVFFTIRWWDP-STVAD-----------------------------
AtrGPCATh   139 LAWALIVWRCSLVFSSLDKIVSVLIHLLPG----IVFFTIRWWDP-TRLGD-----------------------------
LtuGPCATh   189 LAWALIVWRCSLVFSSVDKIVSVLIHLLPG----IVFFTIRWWDP-VTFAA-----------------------------
AfiGPCATh   191 LAWALIVWRCSLVFSSVDKIVSVLIHLLPG----IVFFTIRWWDP-ATFAA-----------------------------
KflGPCATh   176 LAWAIIVWRCSLVFSSLDKIVSVLIHLLPG----VVLFIIRWWDP-SNFSH-----------------------------
EmuGPCATh   123 VYSAIILFRNSFVFHNYDKMTSCFIHVLPP----LISYCVRWFPK-NSSNP-----------------------------
OviGPCATh   105 AYSGAFLFRNALVLHDFQKLVSSFIHILPV----IFSYLLRWFPS-ETSTL-----------------------------
SpuGPCATh   167 VTWSIVLFRNCLVPHSIERMTCLIIHLMPS----LITYLVRWYPT-ETSQH-----------------------------
CgiGPCATh   177 LIWAMVVYRNSLVFHSTDKVTSAYIHILPA----LLSFGIRWYRE-EVSKY-----------------------------
LgiGPCATh1  153 LLWAAVIFRNSLVLHSVDKVTSVYIHTLPA----FLSFALRWYSE-DTSMK-----------------------------
LgiGPCATh2  179 LLWAMVIYRNSLVLHSIDKVTSVYIHILPA----FLSFVIRWYPE-ESSSK-----------------------------
LcoGPCATh   221 VAWAIVTWRNSLVFHSLDKITSVFIHIFPP----LVMYTIRWLPE-LECQSS-----------------------NYRDT
MciGPCATh1  224 VAWAIITWRNSLVFHSLDKVTSVFIHIFPP----LVMYCIRWMPE-LQRDIYC---------------DNTPFVTSYRNE
MciGPCATh2  219 VLWAIITWRNSLVFHSLDKVTSVFIHIFPA----LVTYTLRWFTV-LHGDPDQ---------------A-----LVYRDQ
MveGPCATh   251 LAFAIITWRNSLVLHSLDKVTSVYIHMSPP----ITLYTVRWLYP-DPE-H----------------------------A
UmaGPCATh   246 LAFSVATWRNSLVFHSLEKMTSLFIHLYPP----FVFTTMVHFMP-HDEAV----------------------------S
LbiGPCATh   177 LASAVITWRNSLVFHDQDKVTSLFIHIYPP----FTFTVIRHFYP-NAE------------------------------A
SpoGPCATh   200 LAWSVVAWRNSLLFHSIDKITSLFIHFFPP----LVLHTIVHLTN-KSYLK----------------------------D
SceGPCAT    201 LCFAVITWRNSLVIHSIDKTTSCFIHIIPP----CVMYVIYHGLP-LEYKI----------------------------E
LthGPCATh   196 LSFAVITWRNSLVIHSVDKITSCFIHIMPP----LTMYTIHHGLG-EDLKR----------------------------A
PkuGPCATh   203 LSFAVITWKNKLVLHSIEKITSTFIHVLPS----VTMYVITHQLS-HEYKL----------------------------E
NcrGPCATh   273 NAIAIIMWRNSLVFHSLDKVTSLFIHIMPC----ATLHCIVHLLD-PAEQR----------------------------K
CbaGPCATh   261 NAVAIVMWRNSLVFHSLDKVTSLFIHVMPP----VALHCIVHLTP-DDYLR----------------------------E
AclGPCATh   261 NAVAIAMWRNSMVFHSMDKVVSLFIHIMPP----VTLHCLVHLTP-SKVLR----------------------------E
CglGPCATh   268 NAVAIIMWRNSLVFHSFDKVTSLFIHIMPC----ATLHCIVHLID-PAAQQ----------------------------A
BbaGPCATh   265 NAVAIIMWRNSLVFHSFDKVTSLFIHIMPC----ATLHCLVHLYP-EDKLQ----------------------------G
DdiGPCATh1  359 LAWGTIVWRNSLVFHDVDKLTSVFIHLCPP----IVTYCLRWFPN-VYPEQ-----------------------------
DdiGPCATh2  192 LLGAVIPYRNSMVFHSLDKATSVIVHIFPS----LVTYSLRWNDE-FLFSK-----------------------------
AsuGPCATh   192 LMWAIVAWRNSLVFHSLDKATSVLIHILPA----IVTASVRWHILPATHPI-----------------------------
EdiGPCATh   142 LLTAIPMWRNSLVFHDLDRLTSVYIHLFPG----LVIYCIRWYVL-TTVPQ-----------------------------
AcaGPCATh1  189 LAWSVLAWRNSLVFHSLDKTTSLFIHLTPN----ILLYALRWLDE-DPDSLA----------------------------
AcaGPCATh2  222 LAGAIVMWKNSLVFHDIDKMISLFIHIYPP----IVTYCLRWWHS-DSFVV-----------------------------
PbrGPCATh   255 LAWAVPLWRNSLVFHDLDKLTSCAIHILPM----LLTFQLRWFPS-DSSLSICGD--------------------------
NgrGPCATh1  225 LAIAIVMWRNSLVFHSIEKVTSVFIHIFPA----IVTFALRWLMF-NDNTQ-----------------------------
NgrGPCATh2  179 ILLAIVFWRNSLVFHDFDKLTSAFIHTFPP----LISFAIRWYPE--QFPE-----------------------------
MbrGPCATh   202 LAIAIVAWRNSMVFHSLDKTTSVYLHVLPA----LVSFSWRWDEL-TECHA-----------------------------
BsaGPCATh   187 LAWAVLAFSQSLIFHSAPHMTSVFIHTSPM----LLTFAIRWLLP-DEHKA-----------------------------
OtaGPCATh   168 VLLAIPLWRNSLVFHSVDKVQSVFVHAMPC----LMTWCGRWYGF-GDDENRLSVA------------------------
MpuGPCATh1  194 ILWAIPLWRNSLVFHSHDKVQSVFIHTAPA----ILTYCARWYGQ-DASPLVAIGAWSERV----------------LGT
MpuGPCATh2  151 LAWAIPTFGNALVLHSPKHAVALIVHISPP----FVTWAMRWHAD-AHERA-----------------------------
MspGPCATh1  179 ILWAIPLWRNSLVFHSHDKVQSVFIHTAPA----ILTWCTRWYGQ-GDLTWSWWGL-GDATGWLFAARE-----GDHGQA
MspGPCATh2  143 LGWAVPALANALILHSVEHTAALFIHISPP----MVMWAMRWHAA-EHEAA-----------------------------
CreGPCATh   139 ---KVGAGAGSAVRRAPHQVTSLFLHLVPA----LVSWSLRWYGDPARFGP-----------------------------
CowGPCATh   479 LAWSVITWHNSFVFHDLDKMTSSAIHLFPA----IVTYSIRWYTP-LEGQA-----------------------------
CmeGPCATh   177 VLWAMVAWRNALVFHSLDKMTSVFIHIFPP----LVTYNARWHSD-LIISRRI---------------G-----ICSHE-
                     End of DUF2838|
```

```
PpaGPCATh1  161 H---------------------AVDDT-G----PWPAWP--LLEN-DRQ-LWTWLFVVP-LIAYSVWQILYLLVVNV---
PpaGPCATh2  160 H---------------------AIDDT-G----PWPAYP--LLEN-NQQ-LWTWLFVVP-LIAYTLWQLLYLLVVNV---
SmoGPCATh1  183 H---------------------SLDAT-G----PWPAWP--LVHD-NRA-LWTWLFFVP-LIAYSVWQALYLLIVNV---
SmoGPCATh2  161 H---------------------STELT-G----PWPAWP--LVQT-RTD-LWTWLFAVP-LAAYTVWQILYFLIVNV---
ZmaGPCATh1  189 M---------------------HPEGR-D----ARVTWP--YVED-KSY-MWTWLFFVP-LAAYTLWQLMYFLIVNV---
ZmaGPCATh2  186 M---------------------HPEGR-D----ARVTWP--YVED-KSY-LWTWLFFVP-LAAYTLWQLMYFLIVNV---
OsaGPCATh   208 M---------------------HPEGR-A----ARVTWP--YVGD-KSY-LWTWLFAVP-LAAYTLWQLMYFLIVNV---
MacGPCATh1  200 M---------------------HPAGR-A----ARVSWP--YVED-KSY-LWTWLFVVP-LIAYTLWQILYFLIVDV---
StuGPCATh1  192 M---------------------HPEGT-P----ERASWP--YVES-QSY-LWTWLFWVP-LAAYFLWQVLYYLIVDV---
StuGPCATh2  186 M---------------------HPEGT-P----ERASWP--YVEN-QSY-LWKWLFFVP-LAAYFFWQVLYYLIVEV---
RcoGPCAT    204 M---------------------HPEGT-S----GRASWP--YVED-KSF-LFTWLFLVP-LVAYFLWQLLYFLIVNV---
AthGPCAT    201 M---------------------HPVGT-D----RRVSWP--YVED-KAY-LFTWLFLVP-LVVYTLWQVLYFLIVNV---
BvuGPCATh   256 M---------------------HPEGT-A----RRVSWP--YIED-TSY-LVTWLFLVP-LFAYTLWQVLYFLIVNV---
NnuGPCATh   198 M---------------------HSDGNAG----RRASWP--YVEN-KSY-LWTWLFVVP-LAAYTLWQVLYFLIVNV---
EguGPCATh   204 M---------------------HPEGR-A----ARVFWP--YVED-KSY-LWMWLFCVP-LVAYTLWQLLYFLIVNV---
NadGPCATh   216 I---------------------HLEQKGG----HRTAWP--HVAD-KTT-LWTWLFFVP-LVAYTLWQVLYFLIVNV---
AtrGPCATh   185 V---------------------DAGQQAG----VKAAWP--SVED-NAE-LWTWLFWVP-LVAYTLWQLLYFLIVNV---
LtuGPCATh   235 M---------------------HPEGKAS----DRASWP--YVED-KSY-LWTWLFVVP-LVAYTLWQLLYFLIVDV---
AfiGPCATh   237 M---------------------HPTGEAG----GRSSWP--YVED-KAY-LWKWLFCVP-LVAYTLWQLLYFVIVNV---
KflGPCATh   222 H---------------------SPDEF-GPWGHGHTPWP--VVED-TRK-VWTWLFTVP-LIVYSVWQALYWIIVEV---
EmuGPCATh   169 WWKGFVN-------------T-------------GGSTNV-SVFN-PMD-DWIYLVVIP-NAFFITHTLLYFIIVHV---
OviGPCATh   151 WYTAFSD-------------T-------------GAKPNI-YLWI-HDL-DWFYVMLIP-LTIFTAREILYYLIIYG---
SpuGPCATh   213 WYQDFAN-------------T-------------T---RM--EMA-TGW-EFLWLVAVP-LACYMCHLVLYLALFVW---
CgiGPCATh   223 WFDEF----------------------------------V--PEM-LEL-SPIWLLVVP-FACFLLHSGIYFLVIHI---
LgiGPCATh1  199 WRKDF----------------------------------V--PDI-NNW-SVQWLIVVP-FLGFVLHSILHSILTSI---
LgiGPCATh2  225 WPCHF----------------------------------I--PLL-SEW-SFIWLVAVP-AASFIGHNILVTLLTCI---
LcoGPCATh   273 RFPGVTN-------------------------------------D-ITM-SFKEAMLLS-TIAYLIWQALYFVFIMV---
MciGPCATh1  284 RFPALDN-------------------------------------L-ELP-SFKDVMIYS-TLAYGIWQSLYYLFIMV---
MciGPCATh2  274 HFPAISK-------------------------------------M-PVM-GWWYTLFVS-TGFYLAWQIFYVCFVMV---
MveGPCATh   297 RYPALKN-------------------------------------M-EVL-PTAQSLTAA-IALYLTWQIAYYLLVVV---
UmaGPCATh   293 RFPALKN-------------------------------------L-ITL-NGYTSFWFN-VTIYLIWQLVYYELIVI---
LbiGPCATh   222 RFPALRE-------------------------------------L-EYL-HPLRALLLS-AGIYVVWQFLYWKFVLV---
SpoGPCATh   247 RFPAVLK-------------------------------------V-KKI-DLLSSVEIA-SFFYALWQIWYYFFIQV---
SceGPCAT    248 RFPGAII-------------------------------------Q-SEL-DIKKNILWT-SLYYLVWQSLYHYFITL---
LthGPCATh   243 RFPAASL-------------A--------------G--------S-KKW-NLKYNIFWT-SLYYLLWQSAYHYFITL---
PkuGPCATh   250 RFSGSVK-------------------------------------L-QQW-DIMYGILQT-SLLYLIWQLGYHYFITI---
NcrGPCATh   320 RFPSIWT-------------I-------------KTSPP--GS-PTAYA-NVISMLAWS-TIPYAIWQLAYYFFITV---
CbaGPCATh   308 RFPAVHT-------------I-------------KYSSK--GS-P-GHY-ELAAMLLWA-TIPYAIWQLSYHFFISV---
AclGPCATh   308 RFPAIYD-------------I-------------KFSKP--GS-P-NHY-SLLSMMLWA-TVPYVIWQLTYHFFITV---
CglGPCATh   315 RFPAIWT-------------I-------------KTSPA--GS-STAYA-NVFSMLAWS-SIPYAAWQLMYYFFITV---
BbaGPCATh   312 RFPAIWA-------------I-------------KTAKP--GS-ATAYA-NIVSMLAWS-TIPYALWQLSYYFFITL---
DdiGPCATh1  405 LA--------------------------------------CGGVE-CVL-SFKEIFFVP-FMVYAFWQVFYYIKTEV---
DdiGPCATh2  238 -Q---NQ-------------------------------------I-EEP-NWKNFYLYP-IIAYSIWQLVYFFWVGK---
AsuGPCATh   239 -D---KE-------------------------------------S-LTA-TWTETFWIP-MFFYLLWQTIYVYVML----
EdiGPCATh   188 ------------------------------------------------L-TIMNGLIIP-SFLYILWQIIYLIITEG---
AcaGPCATh1  236 TYET-LR-------------------------------------G-KDA-TFTQMVFLP-IIPYLIWQVLYLIKVQV---
AcaGPCATh2  268 CR--------------------------------------D--AD-CSI-SWTASFWMP-LMLYSFWQALYIFKTEL---
PbrGPCATh   305 ------------------------------------EWP--S--C-GTV-ALKDIAVYP-LLPYVIWQIAYTLKTQSKFL
NgrGPCATh1  271 TPKIITP-------------I-------------GESYSICVNED-CYV-SLVDIYVPH-ICLFIFWQLSYTIKTHF---
NgrGPCATh2  224 YS-------------------------------------VCIREN-CEV-TWFEVIVPH-LLFFLFWQISYFIKTEH---
MbrGPCATh   248 -NPE-LE-------------------------------------M-CQL-SLMDWLVYP-LAFYAAWQVFYLWYTEV---
BsaGPCATh   233 NFTIASA-------------E-------------AP----IYSDP-LNV-STFDLMWKSVVYFYGWWVVLYYVWVFV---
OtaGPCATh   219 ------------------------------------DLD--SDVT-VLQ-GSLDLMVYP-MLAYLLWQTLYLVKTEY---
MpuGPCATh1  253 RLPGLLG----GNA-NRLGVA-------------ELSTP--M-TS-TRE-MLTEFYLWP-TLGYIAWQVAYLLKTEV---
MpuGPCATh2  197 F-PGVFG-------------VPHANSG-D----AKRGTS--Y--A-ESV-TTMDLLVPA-ATLYFAWAGAHVLWLLL---
MspGPCATh1  248 VAPGLMYADVAAVHRAPNRLT-------------VEQLT--MSYD-VVD-VVREFYVYP-VVGYLIWQVIYLVITEL---
MspGPCATh2  189 F-PGILP-------------MPL-------------DAA--N--G-SGL-GYVELITPA-MTVYAVWWIVHVAWLVS---
CreGPCATh   183 PPEATEQ-------------E-------------RE----RW--H-RA--GGLSNVLLP-MASYVVWAVAYYLKIFV---
CowGPCATh   525 SVCSQDD-------------------------------------C-SIP-SVWSWLSSP-LVIYAIWQLSYFLKTEY---
CmeGPCATh   231 -----AD-------------------------------------C-IALGRRAFTVAVP-LVIFLIWQTLYLAKILV---
```

```
PpaGPCATh1  207 --LRHQRLLKDPEVM-TSF-RELSRKA------SR--A---NN---IWWRLSGLL---------------G----D----
PpaGPCATh2  206 --LRRQRLLNDPEVM-TSFSRELSRKA------QR--S---NN---IWWRLSGVL---------------G----D----
SmoGPCATh1  229 --LRKQRLLRDPEVM-TSF-RELSRKA------AR--A---NN---IWWWLSGLL---------------G----E----
SmoGPCATh2  207 --LRRQRLLNDPEIM-TSY-RELSRKA------SR--A---NN---IWWRLSGIL---------------G----A----
ZmaGPCATh1  235 --LRRQRLLRDPEVM-TSY-RELSKKA------QK--A---NN---IWWRLSGLL---------------G----D----
ZmaGPCATh2  232 --LRRQRLLRDPEVM-TSY-RELSKKA------QK--A---NN---IWWRLSGLL---------------G----D----
OsaGPCATh   254 --LRRQRLLKDPEVM-TSY-RELSKKA------QK--A---NN---IWWRLSGLL---------------G----D----
MacGPCATh1  246 --LRRQRLLQDPEVM-TSY-RELSKKA------QK--A---NN---IWWRLSGLL---------------G----D----
StuGPCATh1  238 --LRRQRFLQDPEVM-TSY-RELSKKA------KK--A---NN---VWWRVSGLL---------------G----D----
StuGPCATh2  232 --LRRQRLLRDPEVM-TSY-RELSKKA------KK--A---NN---MWWRLSGLL---------------G----D----
RcoGPCAT    250 --LRRQRLLRDPEVM-TSY-RELSKKA------QK--A---NN---VWWRLSGLL---------------G----D----
AthGPCAT    247 --LRRQRLLRDPEVM-TSY-RELSKKA------EK--A---NN---KLWQLSGLL---------------G----D----
BvuGPCATh   302 --LRRQRLLRDPEVM-TSY-RELSKKA------QK--A---NN---LWWRLSGLL---------------G----D----
NnuGPCATh   245 --LRRQRLLRDPEVM-TSY-RELSKKA------QK--A---NN---IWWRLSGLL---------------G----D----
EguGPCATh   250 --LRRQRLLRDPEVM-TSY-RELSKKA------QK--A---NN---IWWRLSGLL---------------G----D----
NadGPCATh   263 --LRRQRLLRDPEVM-TSY-RELSKKA------KK--A---NN---VWWRLSGVL---------------G----D----
AtrGPCATh   232 --LRRQRLLRDPEVM-TSY-RELSKKA------QK--A---NN---IWWRLSGVL---------------G----D----
LtuGPCATh   282 --LRRQRLLRDPEVM-TSY-RELSKKA------KK--A---NN---LWWRLSGLL---------------G----D----
AfiGPCATh   284 --LRRQRLLRDPEVM-TSF-RELSKKA------QK--A---NN---VWWRLSGLL---------------G----D----
KflGPCATh   272 --LRKQRLLRDPQVL-TSF-RELSRKA------ER--A---NN---LFWWASGLL---------------G----D----
EmuGPCATh   216 --IIKP---N-EKYN-DNY-RYLRTKY------FS--K---VD---FFKR----I---------------P----K----
OviGPCATh   198 --CVKP---S-EKHL-DSY-RYMHKKK------LL--A---HL---LWNR----F---------------H----A----
SpuGPCATh   256 --ALGSTFSS-SEYL-NNY-TFIAYQS--------------NN---VFSRAIRCK---------------G----G----
CgiGPCATh   261 --VAKP---S-EEYV-TSF-NYLGKKQ--------------DS---CLYKTFNVC---------------G----E----
LgiGPCATh1  237 --FP-P---P-EEYL-TSY-KCLTAQE--------------SS---FMFKAVDLL---------------G----P----
LgiGPCATh2  263 --CK-P---P-EHYQ-TSY-RYLTSKT--------------ST---FIYRAINLC---------------G----P----
LcoGPCATh   310 --RRREKVES-GLRM-TSY-SWLLDDS------HG--R---KG---LIQKAAFVF---------------G----A----
MciGPCATh1  321 --GRRDKVES-GLRL-TSY-SWLLDDT------HG--K---KG---FIQKMAFMF---------------G----P----
MciGPCATh2  311 --AKKDKVES-GSRT-TSY-TTLLNKS------PENKN---KS---FILAITSLF---------------G----E----
MveGPCATh   334 --RWREKVES-GKRV-TSY-TWLLNDP------K-------SG---MIAKTAHTF---------------G----E----
UmaGPCATh   330 --RKKSKIET-GERI-NSY-STMSKGK------G-------------PVANLLGKA---------------P----P----
LbiGPCATh   259 --DRRAKIQS-GQRT-TSF-SWLLNDK------Q--------G---MIGRSLASI---------------P----P----
SpoGPCATh   284 --GKQKQIQE-G-RP-TSF-TWLSKAY------S-------KT---KLGRAVAKL---------------P----Q----
SceGPCAT    285 --KKSSKIKS-GERM-TSF-EYLTTHQ------F-------KN---FW---AVKL---------------R----S----
LthGPCATh   282 --RKSSKIKS-GQRA-TSF-EYLTTHQ------F-------KN---FW---AAKL---------------P----E----
PkuGPCATh   287 --RKADKIKK-G-KV-TSF-EYLRKSM------N-------TH---KFVRFINSL---------------P----D----
NcrGPCATh   366 --RRRDKIAA-G-RP-TSF-TWLRKSY------S-------KN---WLGKAILNL---------------P----D----
CbaGPCATh   353 --RRAEKIAA-G-RP-TSF-TWLRRSY------A-------KT---WIGKLVNSL---------------P----E----
AclGPCATh   353 --RRAEKIAA-G-RP-TSF-TWLRKSY------A-------KT---WIGKLVINL---------------P----E----
CglGPCATh   361 --RRREKIAA-G-RP-TSF-TWLRKSY------S-------KV---WIGKIVLGL---------------P----S----
BbaGPCATh   358 --LRREKIAA-G-RP-TSF-TWLRRSY------A-------RT---WIGKIVLSM---------------P----S----
DdiGPCATh1  441 --VDSDKLANDKEIM-TSS-RWMSVKQ------P-------H----PIYRWCQKN---------------G----Y----
DdiGPCATh2  271 --FKKDTIKE-NNYT-TSL-SFLSTIK------EG-EK---GS---LISRYLNFA---------------K----P----
AsuGPCATh   271 --LRQNTIRE-RNYI-TTL-NFLTNHQ----------S---NS---IVSRILNIL---------------P----T----
EdiGPCATh   215 --FKKETIKK-NHYI-TSL-IWLSQEH------P-------H----PVYLYLLKK---------------G----F----
AcaGPCATh1  272 --ISAKKVEE-QDYQ-TTF-RWFSSMD----------K---GG----IGKLIKKA---------------S----P----
AcaGPCATh2  302 --MDKKKLDSDANIM-TSL-RWFIRED------K-------PH---PIYKALLVK---------------G----I----
PbrGPCATh   342 RLSKPVSVADDDLMQ-TSF-KWLLSAN------E-------GP----VFRILQKF--------------PT----L----
NgrGPCATh1  319 --LDREYLQKQNNIQ-TSY-NYLIKSK------A--------F---IQTLSDKLY---------------G----K----
NgrGPCATh2  261 --IDRNILKERSHVI-TSF-RWITEMD------K-------KS---KSYKLGQKF---------------S----A----
MbrGPCATh   283 --LNADHLRTDQALE-TSL-RWLARSP------GG--M---AAVAKTFCRAVGIM---------------K----PDENF
BsaGPCATh   278 --IMGPRIKRR-GYK-TLF-DRVVSRG------P-------T----KFLA-KISK---------------N----D----
OtaGPCATh   255 --VDKAKLDTDPALM-TSL-RWLASDR------K-------SA----ANKFALKTMRGIGLFGTDEFFDPK----T----
MpuGPCATh1  306 --HDVKRLDEDPELV-TSL-RWLAGDY------K-------LA----VTRSTLRTCRRLGFFKVDELFDAS----S----
MpuGPCATh2  248 --FGRHKGAKKTAVYGTPR-AELVSDTVYHYTMRT--VKPFGD---VCGYEV-------------------ARPSK----
MspGPCATh1  307 --FYVKRLDNDPELV-TSL-RWLANDY------K-------AP----VTRWALRTSRRVGLFAQDELFDHN----S----
MspGPCATh2  232 --HGRYHG-HSNTSG-SSH-DTVFHMT------LR--G---NK---VLSRWVGYD---------------NDRPAD----
CreGPCATh   224 --FSRAKIQQR-GYR-TLF-NYVTSQK------K-------G----TFHAIARRI---------------P----P----
CowGPCATh   562 --VDHAKIAANVRLK-TSY-RWLSSSR------G-------H----ISYRIMNKF---------------D----R----
CmeGPCATh   264 --ISRRKLDRNPELI-TSL-RWLTRHS------G--------S---AVSRMLNIF---------------G----E----
```

```
PpaGPCATh1  246 ----N-NRVAMYAILQAIFTVFTMALTI-PLFKHYKVHITFECLKVAASVWNGGIFFFDVLPRKMDAKKKR--------K
PpaGPCATh2  246 ----N-NRVLMYCILQGIFTILTMGLTV-PLFKSYRLHTVFQCLKVAASVWNGGIFFLDVMPRKIDAKKNK--------K
SmoGPCATh1  268 ----R-NRVVMYAAIQAAFTVATMALTV-PMFKSYRLHVLFELFKAAVAVWNGGKWLFDVMPRQMEKKKTGKV--YS-KA
SmoGPCATh2  246 ----Q-NRLVMYAVLQALLTVATMALTV-LMFKSYRLHCAFELLKVAAAIWNGGNFLFEVMPRQADRKKAKAI--AK-LQ
ZmaGPCATh1  274 ----R-NRQVMYILLQALFTVATMALTV-PIFLSYRMHVVFLILKVCASTWNGGSFILEVMPRQVERKQQKKL--GM-KP
ZmaGPCATh2  271 ----R-NRQVMYILLQALFTVATMALTV-PIFLSYRMHVVFLILKVCASTWNGGSFILEVMPRQVVQKQLKKL--DM-KP
OsaGPCATh   293 ----R-NRPLMYILLQALFTVATMALTV-PIFLSFQMHVVFQILKVCASTWNGGSFILEVMPRQVVQKEKKKL--EM-KP
MacGPCATh1  285 ----K-NRQIMFILLQAVFTVATMALAV-PIFMSYKTHVTFQIFKVSAITWNGGSFILDVIPRQAVLREKKKL--EM-KP
StuGPCATh1  277 ----Q-NRLLMYILLQAVFTIATTALTV-PIFLSYKLHVVFQIFKVSASIWNGGNFMLDVMPRQVILKEEKKK--SKMST
StuGPCATh2  271 ----Q-NRLLMFILLQAIFTVATTALTV-PIFLSYKLHMVYQILKVSASVWNGGDFLLEVMPRQVILKEKKKS--ET-RD
RcoGPCAT    289 ----Q-NRMLMYILLQALFTVATTALTV-PIFLSYELHAVFQILKVSAAVWNGGSFLLDVMPRQVILKEKKKS--EL-QP
AthGPCAT    286 ----Q-NRIWMYILFQAIFTVATMALTV-PIFLSYRLHVIFQILKISAAVWNGGSFLLEVMPRQVIQKEKKKK-AEM-QP
BvuGPCATh   341 ----Q-NRLLMYIAFQALFTVATVALTV-PIFLSYELSVIFQILKVSAAVWNGGSFVLEVMPRQVILKEKKKT--QM-QP
NnuGPCATh   284 ----Q-NRMLMYILLQAVFTVATMALTV-PIFLSYEMHVIFQTLKVSATIWNGASFLLEVMPRQVILKEKRKL--EM-QP
EguGPCATh   289 ----Q-NRPTMYILFQGIFTVATMALTV-PIFLSYEMHVIFQILKVSATIWNGGSFLLEVMPRQVVLKEKKKL--EM-KP
NadGPCATh   302 ----Q-NRLLMYIVLQAIFTVATMALTV-PIFMVYELHATFQILKVSAAVWNGGSFLLDVMPRQVVLKDNKRL--EM-QP
AtrGPCATh   271 ----Q-NRALMYIVLQGAFTVATTALTV-PIFKSYKMHFTFQILKVSASVWNGGSFLLEVMPRQVVLKEKKKL--EM-QP
LtuGPCATh   321 ----Q-NRLFMYILLQAAFTVATMALTV-PIFMSYKMHVTFQILKVSASIWNGGSFLLEVMPRQVVLKEKKKL--EM-KP
AfiGPCATh   323 ----Q-NRNFMYMLLQGAFTVATMALTV-PIFLSYELHCIFQILKVSASVWNGGSFLLEVMPRQVVLKEKKKL--EM-KP
KflGPCATh   311 ----A-NRVVMYAILQGLFTIATIALTL-PMFVNYRFHVAFQIFKIVTSVWNGGGYFFDVMPRQIVTKDEKRK--AL-KS
EmuGPCATh   247 ----I-VQTLIWVGLNIIVNFVLNMISL-LAWCTFLFHTIMIVAMVVVMSWYGASYYLDYFAYLALRKAIENN-E-VPA-
OviGPCATh   229 ----R-WHLLIWIVFCILSSVVVLCLAL-IAWCSYLFHSCLIIIQLLTISWNGACYYVDYYPIEVQRRCPQPL-N-KLP-
SpuGPCATh   291 ----K-FRLLVYLIFQGIYASITIAMTW-LWFNFWIAHIVFLCFVFTIATYNGSTYYLDLFPKKAFESDPYRL-C-GKS-
CgiGPCATh   293 ----R-CRPLMFYVFNWLFCIVSLAGVF-IWYHFYTAHCIFLGVMSTVLIMNGGNYYIDVFSKR----------------
LgiGPCATh1  268 ----N-YRVVLYVVFNWIYCLVSVTLSI-IWYNCFLAHAVFIAFIFFIITYNGATYYLDVFSVRKKSDE-----------
LgiGPCATh2  294 ----K-YRILLYLIFNWLFCLASFTMTI-LWYNYFIAHIVYLSLMFLVITFNGARFYLDVFSVRPGISG-----------
LcoGPCATh   348 ----K-YKLYMFMLLQLVYNVVTTMPTY-FLFQHFWLHTLFLITIFTASIFNGASFYIEVFSRRYALEVEKLV-E-RSK-
MciGPCATh1  359 ----K-WKSEMFMFLQLVYNIVTSIPTF-FLFQYFWLHTTFLIVMFAVSVWNGANYYIEVFSRRYISEIESIK-E-EDE-
MciGPCATh2  351 ----K-YKLHMFIFWQFWYTLGTSALTY-FYYKSFWFHSSCLMTMFAVSVWNGASYYIDVFSKHYLDEVERRL-A-EFK-
MveGPCATh   370 ----K-YSIPTFMGLQLIYTFVTCLFAL-LTYRYFRLNTAFLVGLFLVSVWNGACYYMEVFSKQYEKQLSKLA-E-EVS-
UmaGPCATh   364 ----K-RREPAFMLLQFVYTIITTLPAPLLLYPNRTASAVFFAGIFVISVWNGASYYVEVFGRRFEKELLQLR-S-ELE-
LbiGPCATh   294 ----A-YRELTFMGGQLIYAILTELPAIFVFYRSSFWSAAFLIFIFAVSVWNGGGFYIEVFGRKFERELEALR-R-ELA-
SpoGPCATh   319 ----N-LQPFVFMIIQYLYSITTMLPCS-LWYNNKLYSTAFLALIFGWSVWNGASYYIDVFGRRFQKELEALR-Q-QLA-
SceGPCAT    318 ----P-WPMIIYTLSQYFYQLFTMLLCG-IWIRYKLAAALFLTIVFLWASHNGATYYIDHYGKNFEKEVDRLR-L-EVE-
LthGPCATh   315 ----P-WPMFFYILFQYCYQLGTMMLCG-IWFNYRGAAGAFLTFIYLCAAKNGATYYIDYYGKKFEKEVAKLK-S-EVE-
PkuGPCATh   322 ----P-LPLVAFTLIQYGYQLITMSLCP-LLYKHRHLCSAFVSFIFFYATYNGATYYIDYYGKKFQRQVSELQ-R-EIE-
NcrGPCATh   401 ----S-LQEPAFMLIQYTYAVLTMLPCP-LWFYYRWLSAVFLMGVFTWSVYNGSTYYIDVFGKRFQKELEAMK-A-EVA-
CbaGPCATh   388 ----S-LQEPAFMLIQYLYACLTIIPCP-IWFWYRWPSAFFLLFVFSWSVWNGANYYMDIFGKRFQKELEQMK-R-DVA-
AclGPCATh   388 ----A-LQAPAFMMIQYIYAVLTMIPCP-IWFRYRWASGAFLTALFVWSIHNGATYYIDIFGKRFQKELEELK-K-DVA-
CglGPCATh   396 ----A-LQEPAFMLIQYIYAVLTMLPCP-LWFFSRWASSLFLIVVFAWSVYNGSTYYIDVFGKRFQKELEAMK-A-EVA-
BbaGPCATh   393 ----T-LQEPTFMMIQYVYAVLTMLPCP-LWFISRYASTLFLLVVFAWSIYNGSTYYIDVFGKRFQKELEAMK-T-EVV-
DdiGPCATh1  477 ----NVSPVVILILVQMLYTLATCISLP-FLIQSFWLHTVFMIYIFVTISWNGASYYFEVFSENYSSRLNSEF-K-HKH-
DdiGPCATh2  310 ----N-HRLFFFIFSQFTYTLLTILPCW-LYYKYQVIHSLYLIFVVTVCLWNGANFYIEYFSSNYVAQMKARE-E-RWT-
AsuGPCATh   307 ------KKVYIFMLSQLLYTILTMLPCK-ILYNNMNLHLGYIVCMLLVCIWNGANYYIEFFSTHYVKQLKEKE-Q-RWK-
EdiGPCATh   250 ----KDRPLVILITVQFIYTIITILPTF-IYYHYQYLELLWILFCVTWAISNGANFYFEVFIKKYSKRVDESV-K-ELR-
AcaGPCATh1  307 ----N-RRLAAFVGYQLLYTIVTMLPSV-LFLRYFWAHTILIAGMCILSAWNGANYYFEVFSTRYMESLERGT-E-RIA-
AcaGPCATh2  339 ----RIKPLVLLVLIQFTYTMLTLIPMH-FAYQNFYVHSAVLFFVFCACTWYGATFYFEVFSESYSKRLSTRL-K-SGT-
PbrGPCATh   381 ----A-AKEAAFIVLQFLFTFVTMVPTL-LYFHSFAIHLIFLCLIITLAAWNGANFYVEVWTSRYKWANRAKL-E-AAA-
NgrGPCATh1  355 ----E-HRLLGFISLQFLFHMCTLIPIK-FMFDHFWVHVAVLTSVFLVSVWNGANFYFERFAKVYLHDLRKMA-E-KLE-
NgrGPCATh2  298 ----T-TRLFAFMAFQLFYHSLTVLPVI-FMFRYIYFHAFCLVAIYIVMVHNGSTFYFEKFVKMVAEQQKQAT-Q-QMA-
MbrGPCATh   329 DSESM-KTKLIFVACQFIFTIFTILPCK-LYFDYYLVNVGFLLFIYIACLYNGASYYIHVFSKRYVEQFRREI-E-TAA-
BsaGPCATh   312 ----L-IQKAAYMLTHFMFALFTMLLTT-VYWRSFWIHVAFVLVIISVSAWNASGFYFTVFLAKYEEELRERA-G-LPS-
OtaGPCATh   306 ----W-KTKFVFVMFQLCYTIVTFIPIP-FCYLNYYAHTALMWVCFGTSVYNGAVYYIDIFSTRYVERLALAE-E-FIR-
MpuGPCATh1  357 ----I-KTKMVFVTLQLAYTVATFLPIP-LCYRYKTFHTALVGLTFLSCIWNGACYYIDIFSRRYIKRFEDGS-N-GAN-
MpuGPCATh2  297 ----A-SPILWYMAWHAGGCVGPMVLLG-VWWHHYYAHCALLLMTVASCAWHGAGQYAHAMLNQNERKIRALL--PD-DD
MspGPCATh1  358 ----L-KTKFVFVSLQLVYTMVTFLPIP-MCYASKAVHTTVMLLVFLSCIWNGAEYYIDIFSRRYAKKFDDGT-F-MGS-
MspGPCATh2  274 ----I-GPCVRYMVLHAAACLLVTLVAP-VFWYNFWAHTCFVLMLLASSIWNGSRRYYGMMTSSYERRLKALL--PE-HA
CreGPCATh   259 ----P-LQPPVYLFLHLCFCMTTFLVAL-GCWYSFWAHTALLAAVASASIWHGGSYYFEVFARKYHEHLMPKA-A-AEP-
CowGPCATh   598 ----R-FWPTVYMGIQLLFTVLTILPTI-LFFQSFVMHTIFLLGLLLAAIWNGANYYVTFLFERTRAQHAPPP-A--PI-
CmeGPCATh   300 ----R-YQNLALAFWQLIFTGVTCLPTA-LFWRYKWMHEIVLAFVGLIVVWNGSNYYFEVFASRYTRRMLEYARD-RVQ-
```

```
PpaGPCATh1  312 --------------------------------------------------------------------------------
PpaGPCATh2  312 --------------------------------------------------------------------------------
SmoGPCATh1  339 K-TESSPENPSKNVISIQ-------------------EESSGG--TAPRGLLSTNC-----------NGSVSSVSS--E-
SmoGPCATh2  317 --------------------------------------------------------------------------------
ZmaGPCATh1  345 I-EQGSWTQGAPAD-GTY-------------------IG-----------------------------------------
ZmaGPCATh2  342 M-EQGSSTQGAPGG-DGG-------------------TLGNHH--------------------------QHT-SEE----
OsaGPCATh   364 M-EEANSSQNAEES-QGD-------------------LSANG---------------------------QHS-SEH----
MacGPCATh1  356 I-VEEPDRLSADLS-TSS-------------------HATSG---------------------------EPS-G------
StuGPCATh1  349 V-PDQNDEISLQES-ATE-------------------TNNTSE--------------------------TVH-S------
StuGPCATh2  342 V-TEQQHENPLQEN-SMK-------------------TP-----------------------------------------
RcoGPCAT    360 A-HIQQYHSEPKQD-QSP-------------------NSMEIR--------------------------MKT-IHS----
AthGPCAT    358 I-EEQILHHEAVSH-PTE-------------------NEPKST----------------------------E--------
BvuGPCATh   412 L-EAQPQLDSSSPL-DSP-------------------VSAQYI---------------------------QQS-------
NnuGPCATh   355 L-PIQPNQTVATVE-PSE-------------------DAKEPV----------------------------K-IDH----
EguGPCATh   360 I-LEQPRQPVVDLS-TNH-------------------QHTSDT--------------------------PDE--------
NadGPCATh   373 I-LSCESTQAQKEV-AVP-------------------DGLEK---------------------------SGDTNNA--QK
AtrGPCATh   342 I-TIPIDQPVESVP-MNM-------------------EKNECN--------------------------SLDPPDA--EP
LtuGPCATh   392 I-PMQQQLDLSLAT-DTL-------------------THSD---------------------------------------
AfiGPCATh   394 I-PTADQPVFDSHA-END-------------------YTSVGD--------------------------YPSRVDG----
KflGPCATh   382 K-QGDAAKDTISQP-NSP-------------------GEVCQK--------------------------CGS-LHSSASK
EmuGPCATh   318 P-VNSKSPTEMEDENDED--------------------EE--------------VD-----------E------------
OviGPCATh   300 LEVLNPEDHELLKNIDEG--------------------KN--------------PG-----------G--MCE-------
SpuGPCATh   362 PE------------------------------------------------------------------------------
CgiGPCATh       --------------------------------------------------------------------------------
LgiGPCATh1      --------------------------------------------------------------------------------
LgiGPCATh2      --------------------------------------------------------------------------------
LcoGPCATh   419 S-GKQDMDKSKT--------------------------------------------------------------------
MciGPCATh1  430 K-LKA---------------------------------------------------------------------------
MciGPCATh2  422 E-KNQQNSKILT---------------------KQRSLR------R--------KQ-----------QKHLDD-------
MveGPCATh   441 S-AVAANQLAHDADEDKR---------------------H--------------EE-----------EELHKQ-------
UmaGPCATh   436 L-IRTAEKVVADAGKKHK--------------------SG--------------ME-----------QEGHVE-------
LbiGPCATh   366 E-STARSSGSSTPTTICS--------------------GP--------------SE-----------TDLTAM-------
SpoGPCATh   390 E-TPTNSGSSSALSR-----------------------------------------------------------------
SceGPCAT    389 N-LQQKLQPDSDAVISDA--------------------SV--------------ND-----------KDYLNV-------
LthGPCATh   386 D-LQQQLSQKANSSSNNY--------------------SG--------------VL------------------------
PkuGPCATh   393 A-MQQDIEERDKSGLSKV--------------------AT--------------GI-----------E------------
NcrGPCATh   472 K-WQHSPDGMVHSPTLTP--------------------HA--------------GA-----------QEEENK-------
CbaGPCATh   459 K-WQNSPGAMFSPPNGPV--------------------DS--------------DV-----------N-LNTA-------
AclGPCATh   459 R-WQASPAGTTSPTLLDS--------------------EN--------------AS-----------AAGARI-------
CglGPCATh   467 K-WQHSPDVWPHEDGSGT--------------------AV--------------VS-----------PVASPA-------
BbaGPCATh   464 K-WQTSPELMLTSPLMQP--------------------QT--------------ES-----------VVIDPQ-------
DdiGPCATh1  549 N-KDQPLKITSIK--AFL--------------------KF--------------LA-----------L-FLTS-------
DdiGPCATh2  381 K-IIMSSIGNTTSFNSNCDSPIMEYSKQQQQSNTLNSTHHKKEKPK--------DHYQQLTPPNLKHQKGHET-------
AsuGPCATh   377 K-TIVSSFGAGDMSPMMQ-------------------SSTNQST------------------------------------
EdiGPCATh   322 R-SESNTSIQTPKILDER--------------------ST--------------SP-----------N-ALSE-------
AcaGPCATh1  378 R-LRRGSLALQQAINIGD--------------------DAEKSQ------------------------------------
AcaGPCATh2  411 S-EAKEKAKEKEKAKKGL--------------------PT--------------KN-----------S-FLSF-------
PbrGPCATh   452 R-ELSAVSQPDVYHGS----------------------------------------------------------------
NgrGPCATh1  426 M-ENKEQTSAITQ-------------------------------------------------------------------
NgrGPCATh2  369 H-PPQTNQK---Q-------------------------------------------------------------------
MbrGPCATh   404 V-AQEAKIEAIAELA-------------------EEQAS-KDAQ-E---------------------QR-----------
BsaGPCATh   383 S-QGADGTTPLLS--SPR--------------------GA--------------KK-----------D------------
OtaGPCATh   377 C-QMVNELTESELETMQT--------------------MD--------------FT-----------DSEIE--------
MpuGPCATh1  428 L-RHEDQQDGIIVTEVTG--------------------RK--------------NT-----------PPEGD--------
MpuGPCATh2  368 --EDGGKKK-----------------------------K-----------------------------------------
MspGPCATh1  429 V-RDDDNADAAVAEAVLA--------------------AG--------------SG-----------DLARR--------
MspGPCATh2  345 --RDNAPSEAPKKH-EH--------------------VE-----------------------------------------
CreGPCATh   330 A-PAHVSASATKK--D----------------------------------------------------------------
CowGPCATh   668 V-PSVVSPPA--D--V-----------------------A--------------GK-----------E------------
CmeGPCATh   372 K-IETNASSNPE----------------------KSSM------------------------------------------
```

```
PpaGPCATh1        --------------------------------------------------------------------------------
PpaGPCATh2        --------------------------------------------------------------------------------
SmoGPCATh1    383 E------------------KKA---------------------------------ICS--LCQQL-SSRKFE---LQK--
SmoGPCATh2        --------------------------------------------------------------------------------
ZmaGPCATh1        --------------------------------------------------------------------------------
ZmaGPCATh2    370 --------------------------------------------------------------------------------
OsaGPCATh     391 --------------------------------------------------------------------------------
MacGPCATh1    381 --------------------------------------------------------------------------------
StuGPCATh1    375 --------------------------------------------------------------------------------
StuGPCATh2    360 --------------------------------------------------------------------------------
RcoGPCAT      388 --------------------------------------------------------------------------------
AthGPCAT      381 --------------------------------------------------------------------------------
BvuGPCATh         --------------------------------------------------------------------------------
NnuGPCATh     381 --------------------------------------------------------------------------------
EguGPCATh     385 --------------------------------------------------------------------------------
NadGPCATh     403 A-------------------------------------------------------------------------------
AtrGPCATh     373 L-------------------------------------------------------------------------------
LtuGPCATh     412 --------------------------------------------------------------------------------
AfiGPCATh     423 Q--------------------N---------------------------------GHP--VVDAN-ESKLVA---DAN--
KflGPCATh     414 DSLNGSLSGSGELET---NGDG---------------------------------LKG--QMDAE---------------
EmuGPCATh     340 --------------------------------------------------------------------------------
OviGPCATh     326 --------------S---DADVPCRLV----------------------SCESAHKIT--ELT---CH---------SAH
SpuGPCATh         --------------------------------------------------------------------------------
CgiGPCATh         --------------------------------------------------------------------------------
LgiGPCATh1        --------------------------------------------------------------------------------
LgiGPCATh2        --------------------------------------------------------------------------------
LcoGPCATh     430 --------------------------------------------------------------------------------
MciGPCATh1    434 --------------------------------------------------------------------------------
MciGPCATh2    448 --------------K-----------------------------------------------------------------
MveGPCATh     467 --------------G---REQ-----------------E-------------------------------------EE--
UmaGPCATh     463 --------------E---DRD-----------------EDKE------------------QQAAA---AAA---AAAS--
LbiGPCATh     393 --------------V---SAS-----------------KQPG-------------PAS--DISE----------DSPT--
SpoGPCATh         --------------------------------------------------------------------------------
SceGPCAT      416 --------------N---RDE-----------------DFDD----------------------------------SS--
LthGPCATh     407 --------------------------------------------------------------------------------
PkuGPCATh     415 --------------------------------------------------------------------------------
NcrGPCATh     499 --------------R---LGE------P--TKNNNNSDSNDSSGSSTSSL----DNIP--LLTEEKSELPASATATATG-
CbaGPCATh     485 --------------K---D-------------------DSSKGHDKRQSI----DKIP--LLDETGQPQQN---------
AclGPCATh     486 --------------L---DDS-----------------TSAHKECDKASL----DQIP--PLDAHAATTGS---------
CglGPCATh     494 --------------A---GAS-----TTGSLADGISGKRLDDDMRGVSGV----DNIP--LLNDERPAAVL---ATGAD-
BbaGPCATh     491 --------------P---KKA------SGTPSFEPSAQTARDAMARTTSL----DRIP--LLDDYTTANST---------
DdiGPCATh1    573 -------------------------------------------------------------LIV----------------
DdiGPCATh2    445 --------------QFDSDTS---------------------------YI----G---------------------HE--
AsuGPCATh     401 ----------------TSDNA----------------------------I----G-------------------------
EdiGPCATh     348 --------------T-----------------------------------------------------------------
AcaGPCATh1        --------------------------------------------------------------------------------
AcaGPCATh2    437 --------------C---S-F------------------------------------FVPALTI----------------
PbrGPCATh         --------------------------------------------------------------------------------
NgrGPCATh1        --------------------------------------------------------------------------------
NgrGPCATh2        --------------------------------------------------------------------------------
MbrGPCATh     430 ----------------APEQD----------------------------V----Q-------------------------
BsaGPCATh     403 --------------------------------------------------------------------------------
OtaGPCATh     403 --------------------------------------------------------------------------------
MpuGPCATh1    454 --------------------------------------------------------------------------------
MpuGPCATh2        --------------------------------------------------------------------------------
MspGPCATh1    455 --------------S---G-------------------------------------------------------------
MspGPCATh2        --------------------------------------------------------------------------------
CreGPCATh     343 --------------------------------------------------------------------------------
CowGPCATh     683 --------------------------------------------------------------------------------
CmeGPCATh         --------------------------------------------------------------------------------
```

```
PpaGPCATh1       --------------------------------------------------------------------------------
PpaGPCATh2       --------------------------------------------------------------------------------
SmoGPCATh1  404  ------A-------------V-EEA-----TSSVRDLG--------------LEPALFSRIK------------------
SmoGPCATh2       --------------------------------------------------------------------------------
ZmaGPCATh1       --------------------------------------------------------------------------------
ZmaGPCATh2  370  --------------------------------------------------------------------------------
OsaGPCATh   391  --------------------------------------------------------------------------------
MacGPCATh1  381  --------------------------------------------------------------------------------
StuGPCATh1  375  --------------------------------------------------------------------------------
StuGPCATh2  360  --------------------------------------------------------------------------------
RcoGPCAT    388  --------------------------------------------------------------------------------
AthGPCAT    381  --------------------------------------------------------------------------------
BvuGPCATh        --------------------------------------------------------------------------------
NnuGPCATh   381  --------------------------------------------------------------------------------
EguGPCATh   385  --------------------------------------------------------------------------------
NadGPCATh   404  ----------------------E---------------------------------------------------------
AtrGPCATh   374  ----------------------C---------------------------------------------------------
LtuGPCATh   412  --------------------------------------------------------------------------------
AfiGPCATh   442  ------Q-------------V-D------------------------------------LLS------------------
KflGPCATh   441  ------------------------------------------------------M-KLRLGNGKSEEEAKGEKVLGKNGV
EmuGPCATh   340  SLRHN----LE-------TAL-EED-----RDS--D-------------------I------------------------
OviGPCATh   353  SVDSSTNVMLE----YDSMAS-KER-----NGD--N-------------------I------------------------
SpuGPCATh        --------------------------------------------------------------------------------
CgiGPCATh        --------------------------------------------------------------------------------
LgiGPCATh1       --------------------------------------------------------------------------------
LgiGPCATh2       --------------------------------------------------------------------------------
LcoGPCATh   430  --------------------------------------------------------------------------------
MciGPCATh1  434  --------------------------------------------------------------------------------
MciGPCATh2  449  --------------------------------------------------------------------------------
MveGPCATh   474  ------GV-----------VV-EDK---K--NI-----------------------------------------------
UmaGPCATh   483  ------GL--P--Q-----AG-ENK---K--DQ-----------------------------------------------
LbiGPCATh   412  ------SL--DPTL-----NL-DFK---K--DS-----------------------------------------------
SpoGPCATh        --------------------------------------------------------------------------------
SceGPCAT    426  -------------------SV-SS-------KS--D--------------------------------------------
LthGPCATh   407  -----------------------------------E--------------------------------------------
PkuGPCATh   415  -----------------------------------S--------------------------------------------
NcrGPCATh   547  -----AEV--D--GGAKD-VA-RAR---R-PGA--E-LFG--AG--------AAPS------------------------
CbaGPCATh   514  ------GN--A--NGGPN-SEANAR---A-TGA--E-ILYEAKGNVLDRKNVSAPL------------------------
AclGPCATh   517  ------DK--A--NITTD-TM-RER---R---------------------------------------------------
CglGPCATh   542  -----MDM--D--GGARD-VA-RER---RMGSG--E-S-----------------R------------------------
BbaGPCATh   533  ------GF--D--GGARD-VA-RER---K--ST----------------------E------------------------
DdiGPCATh1  576  -YLK-----------LIL--------------------------------------------------------------
DdiGPCATh2  457  ------DL--A--PDTDD-VH-HHYNYS---S---------------------KPN------------------------
AsuGPCATh   408  -------------------------EVG---S---------------------SPQ------------------------
EdiGPCATh   349  ------------------------------SDA--E--------------------------------------------
AcaGPCATh1       --------------------------------------------------------------------------------
AcaGPCATh2  447  -LYF-----------LIERAL-AFA-----T-------------------------------------------------
PbrGPCATh        --------------------------------------------------------------------------------
NgrGPCATh1       --------------------------------------------------------------------------------
NgrGPCATh2       --------------------------------------------------------------------------------
MbrGPCATh   437  -------------------------ELNE--ST--E-----------------DPF------------------------
BsaGPCATh   403  -----------------------------------Q--------------------------------------------
OtaGPCATh   403  -------------------------------------------------QILMTPV------------------------
MpuGPCATh1  454  -----------------------------------------------------GRR------------------------
MpuGPCATh2       --------------------------------------------------------------------------------
MspGPCATh1  457  ---------------GGD-LA-RRS-----GGG--D-L----------ARRSGGPQ------------------------
MspGPCATh2       --------------------------------------------------------------------------------
CreGPCATh   343  -----------------------------------A--------------------------------------------
CowGPCATh   683  -----------------------------------Q--------------------------------------------
CmeGPCATh        --------------------------------------------------------------------------------
```

```
PpaGPCATh1      ----------------------------------------
PpaGPCATh2      ----------------------------------------
SmoGPCATh1  427 --------------------PT-----D-----AI-SCAS
SmoGPCATh2      ----------------------------------------
ZmaGPCATh1      ----------------------------------------
ZmaGPCATh2  370 --------------------HI-----Q-----E------
OsaGPCATh   391 ---------------------------------S------
MacGPCATh1  381 ---------------------------------M------
StuGPCATh1  375 ----------------------------------E-----
StuGPCATh2  360 ----------------------------------Q-----
RcoGPCAT    388 --------------------AE-----E------Q-----
AthGPCAT    381 ----------------------------------T-----
BvuGPCATh       ----------------------------------------
NnuGPCATh   381 ----------------------------------F-----
EguGPCATh   385 --------------------QN-----N------S-----
NadGPCATh   405 --------------------VN-----N------S-----
AtrGPCATh   375 --------------------RT-----N-----SL-----
LtuGPCATh   412 ----------------------------------Y-----
AfiGPCATh   448 --------------------SG-----D------E-----
KflGPCATh   466 ENGNGLHQRSIARANSCGTESGDKVSVEMNGKGSSLGLAR
EmuGPCATh   358 -------------------------EV-------------
OviGPCATh   378 -------------------------AV-------------
SpuGPCATh       ----------------------------------------
CgiGPCATh       ----------------------------------------
LgiGPCATh1      ----------------------------------------
LgiGPCATh2      ----------------------------------------
LcoGPCATh   430 -------------------------A--------------
MciGPCATh1  434 -------------------------K--------------
MciGPCATh2  449 ------------------------LN--------------
MveGPCATh       ----------------------------------------
UmaGPCATh       ----------------------------------------
LbiGPCATh       ----------------------------------------
SpoGPCATh       ----------------------------------------
SceGPCAT        ----------------------------------------
LthGPCATh       ----------------------------------------
PkuGPCATh       ----------------------------------------
NcrGPCATh       ----------------------------------------
CbaGPCATh   552 ------------------------TGA-----------TM
AclGPCATh       ----------------------------------------
CglGPCATh       ----------------------------------------
BbaGPCATh       ----------------------------------------
DdiGPCATh1      ----------------------------------------
DdiGPCATh2  477 --------------------------Q-------------
AsuGPCATh   415 -----------------------LLSV-------------
EdiGPCATh       ----------------------------------------
AcaGPCATh1      ----------------------------------------
AcaGPCATh2      ----------------------------------------
PbrGPCATh       ----------------------------------------
NgrGPCATh1      ----------------------------------------
NgrGPCATh2      ----------------------------------------
MbrGPCATh   447 -----------------------KL-N-------------
BsaGPCATh       ----------------------------------------
OtaGPCATh   410 --------------------------------------D-
MpuGPCATh1  457 ------------------------E-------------T-
MpuGPCATh2      ----------------------------------------
MspGPCATh1  478 ------------------------KSL-----------R-
MspGPCATh2      ----------------------------------------
CreGPCATh       ----------------------------------------
CowGPCATh       ----------------------------------------
CmeGPCATh       ----------------------------------------
```

Appendix S1. **Sequence alignment analysis of GPCATs.**

The protein sequences of GPCATs were aligned using the T-Coffee PSI/TM-Coffee web-service (http://tcoffee.crg.cat/apps/tcoffee/do:tmcoffee) and BoxShade (http://www.ch.embnet.org) was used for shading background according to conservation. Sequence numbering is according to the full-length protein sequences. See Fig. 3 for species abbreviations. The proteins for which catalytic GPCAT activity was determined (Fig. 7) have names highlighted in green. Sequence IDs: AcaGPCATh1, XP_004338657.1; AcaGPCATh2, XP_004368210.1; AclGPCATh, XP_001270843.1; AfiGPCATh, b3_c2279; AsuGPCATh, XP_012748160.1; AthGPCAT, NP_198396.1; AtrGPCATh, XP_011622896.1; BbaGPCATh, KGQ05851.1; BsaGPCATh, CUE73576.1; BvuGPCATh, XP_010666443.1;

CbaGPCATh, KIW96763.1; CgiGPCATh, EKC38105.1; CglGPCATh, XP_001229114.1; CmeGPCATh, XP_005537575.1; CowGPCATh, XP_004348404.1; CreGPCATh, XP_001693172.1; DdiGPCATh1, XP_636790.1; DdiGPCATh2, XP_643621.1; EdiGPCATh, XP_001738020.1; EguGPCATh, XP_010936661.1; EmuGPCATh, CDS42477.1; KflGPCATh, kfl00299_0090; LbiGPCATh, XP_001876784.1; LcoGPCATh, CDH53915.1; LgiGPCATh1, ESO91084.1; LgiGPCATh2, XP_009058350.1; LthGPCATh, XP_002553792.1; LtuGPCATh, b4_c3038; MacGPCATh, XP_009407293.1; MbrGPCATh, XP_001747618.1; MciGPCATh1, EPB88260.1; MciGPCATh2, EPB92530.1; MpuGPCATh1, XP_003062419.1; MpuGPCATh2, XP_003058481.1; MspGPCATh1, XP_002501336.1; MspGPCATh2, XP_002508227.1; MveGPCATh, KFH63290.1; NadGPCATh, b3_c6890; NcrGPCATh, XP_011394510.1; NgrGPCATh1, XP_002675713.1; NgrGPCATh2, XP_002673223.1; NnuGPCATh, XP_010273972.1; OsaGPCATh, EAY74703.1; OtaGPCATh, XP_003078006.1; OviGPCATh, XP_009166064.1; PbrGPCATh, CEO94537.1; PkuGPCATh, KGK40379.1; PpaGPCATh1, XP_001771632.1; PpaGPCATh2, XP_001756364.1; RcoGPCAT, XP_002514086.1; SceGPCAT, NP_011665.1; SmoGPCATh1, XP_002985957.1; SmoGPCATh2, XP_002979497.1; SpoGPCATh, NP_596320.2; SpuGPCATh, XP_003729899.1; StuGPCATh1, XP_006342345.1; StuGPCATh2, XP_006338125.1; UmaGPCATh, XP_011391556.1; ZmaGPCATh1, XP_008656604.1; ZmaGPCATh2, NP_001130177.1
